# Embryo selection by non-invasive preimplantation genetic testing for aneuploidy in women of advanced maternal age: a randomized, clinical trial

**DOI:** 10.1038/s41467-026-77628-4

**Published:** 2026-09-08

**Authors:** Jin Huang, Rong Li, Lin Zeng, Liang Hu, Liyi Cai, Li Chen, Juanzi Shi, Yan Wang, Yichun Guan, Yanwen Xu, Weiping Qian, Xiuxia Wang, Haitao Xi, Zhiming Zhao, Yuanqing Yao, Sijia Lu, Yaxin Yao, Jing Wang, Ping Liu, Jie Qiao

**Affiliations:** 1 State Key Laboratory of Female Fertility Promotion, Center for Reproductive Medicine, Department of Obstetrics and Gynecology, Peking University Third Hospital, Beijing, China; 2 National Clinical Research Center for Obstetrics and Gynecology (Peking University Third Hospital), Beijing, China; 3 Key Laboratory of Assisted Reproduction (Peking University), Ministry of Education, Beijing, China; 4 Beijing Key Laboratory of Collaborative Innovation in Frontier Technologies for Population Quality, Beijing, China; 5 Research Center of Clinical Epidemiology, Peking University Third Hospital, Beijing, China; 6 Institute of Reproductive and Stem Cell Engineering, NHC Key Laboratory of Human Stem Cell and Reproductive Engineering, School of Basic Medical Science, Central South University, Changsha, China; 7 Clinical Research Center for Reproduction and Genetics in Hunan Province, Reproductive and Genetic Hospital of CITIC-XIANGYA, Changsha, China; 8 Hunan International Scientific and Technological Cooperation Base of Development and Carcinogenesis, Changsha, China; 9 Reproductive Medical Center of Hebei Maternity Hospital, Shijiazhuang, China; 10 ShiJiazhuang Technology Innovation Center of Precision Prevention and Control of Birth Defects, Hebei, China; 11 Department of Reproductive Medicine, Affiliated Jinling Hospital, Medical School of Nanjing University, Nanjing, China; 12 The Assisted Reproduction Center, Northwest Women’s and Children’s Hospital, Xi’an, China; 13 Department of Obstetrics and Gynecology, Sichuan University West China Second University Hospital, Chengdu, China; 14 Key Laboratory of Birth Defects and Related Diseases of Women and Children (Sichuan University), Ministry of Education, Chengdu, China; 15 Reproductive Medicine Center, The Third Affiliated Hospital of Zhengzhou University, Zhengzhou, China; 16 Guangdong Provincial Key Laboratory of Reproductive Medicine, Guangdong Provincial Clinical Research Center for obstetrical and gynecological diseases, Center for Reproductive Medicine and Department of Gynecology & Obstetrics, the First Affiliated Hospital, Sun Yat-Sen University, Guangzhou, PR China; 17 The Reproductive Medicine Center, Peking University Shenzhen Hospital, Shenzhen, China; 18 Center of Reproductive Medicine, Department of Obstetrics and Gynecology, Shengjing Hospital of China Medical University, Shenyang, China; 19 Reproductive Medicine Center, Department of Obstetrics and Gynecology, the Second Affiliated Hospital and Yuying Children’s Hospital of Wenzhou Medical University, Wenzhou, China; 20 Department of Reproductive Medicine, The Second Hospital of Hebei Medical University, Shijiazhuang, Hebei, China; 21 Department of Obstetrics and Gynecology, Chinese People’s Liberation Army General Hospital, Beijing, China; 22 Yikon Genomics Co., Ltd., Shanghai, China; 23 Beijing Advanced Innovation Center for Genomics, Beijing, China; 24 Peking-Tsinghua Center for Life Sciences, Peking University, Beijing, China

**Keywords:** Randomized controlled trials, Genetics research

## Abstract

Non-invasive preimplantation genetic testing for aneuploidy (niPGT-A) can detect embryo chromosomal aneuploidy by analyzing the cell-free DNA in embryo culture media. However, evidence for its clinical efficacy is insufficient. In this investigator-initiated, multicenter, double-blind, randomised controlled trial, women aged 35-42 who agreed to single frozen-thawed blastocyst transfer with at least two blastocysts were enrolled from 13 fertility centers in China. Eligible participants were randomly assigned (1:1) to the niPGT-A(*n* = 594) or morphology group(*n* = 595) using a computer-generated block randomization list, stratified by participating center. In the niPGT-A group, embryos were selected for the first transfer cycle based on niPGT-A results, whereas in the morphology group, embryos were selected according to standard morphological criteria. The primary outcome was the ongoing pregnancy rate (OPR) (pregnancy beyond 12 weeks). Secondary outcomes included clinical pregnancy, miscarriage (pregnancy loss before the 28th week, with those before the 12th week as early miscarriages), and live birth rates. The trial has been completed. The modified intention-to-treat population(mITT) contained 581 couples in the morphology group and 571 in the niPGT-A group. Among 1152 randomised patients, OPR was 38.7% (221/571) in the niPGT-A group and 36.8% (214/581) in the morphology group (adjusted *p* = 0.49). There were no statistically significant between group differences in the rates of clinical pregnancy (47.6% vs 49.9%, adjusted *p* = 0.49), miscarriage (21.0% vs 27.6%, adjusted *p* = 0.06) and live birth (37.0% vs 35.5%, adjusted *p* = 0.59). Early miscarriage was significantly lower in the niPGT-A group compared with that of the morphology group (18.0% vs 25.2%, adjusted *p* = 0.03). Maternal and neonatal outcomes did not differ significantly between groups. No serious adverse events were reported in either group. The results indicated that there was insufficient evidence to establish a statistically significant difference in OPR between the two treatment arms. The results of this trial do not provide a basis for recommending routine use of niPGT-A in this good-prognosis population (NCT04339166).

## Abstract (web summary)

Non-invasive preimplantation genetic testing for aneuploidy (niPGT-A) can detect embryo chromosomal aneuploidy by analyzing the cell-free DNA in embryo culture media, but evidence for clinical efficacy is insufficient. Here the authors report a randomised controlled trial involving 1,152 women aged 35–42 years that shows no difference in the primary outcome of ongoing pregnancy rate between the intervention group with niPGT-A and the control group with morphological selection.

## Introduction

Since the birth of the first “test-tube baby,” the use of in vitro fertilization (IVF) has dramatically increased. In 2020, ~3 million IVF cycles were performed globally^1^. Despite considerable advances in IVF technology, the live birth rate remains 40–50%^2,3^. Embryo assessment is critical in IVF treatment. In addition to the morphological score, preimplantation genetic testing for aneuploidy (PGT-A) has been developed for embryo selection because embryo ploidy affects the success of treatment.

Embryonic aneuploidies, which are more common in advanced maternal age (AMA), may result in implantation failure or spontaneous abortion^4,5^. AMA is therefore recognized as one of the indications for PGT-A, according to both the ESHRE good practice recommendations and the ASRM committee opinion^6,7^. According to an ESHRE report, AMA was the most common indication for PGT-A, accounting for 67% of cases, followed by recurrent miscarriage (8%), repeated implantation failure (6%), and severe male factor (3%)^8^. Based on national data from the Society for Assisted Reproductive Technology (SART), the proportion of IVF cycles using PGT has increased from 14% in 2014 to 44% in 2019^9,10^.

However, the benefits of PGT-A remain controversial. Some trials showed no improvement in outcomes in women aged <37 years with PGT-A^11^. Additionally, owing to embryo mosaicism, a single trophectoderm (TE) biopsy cannot represent the whole embryo^12^. Gleicher et al. proposed that PGT-A lacks clinical validation for accurately categorizing the embryo chromosomal status, and its high false-positive rate may reduce live birth chances in many patients^13^. Furthermore, while some studies suggest that TE biopsy may have minimal impact on embryo viability^14^, other evidence indicates that the biopsied TE cell number in blastocysts could affect clinical outcomes, especially in blastocysts with poor TE quality^15^. A non-invasive approach needs to be pursued to overcome the potential limitations of embryo biopsy.

The discovery of DNA in spent culture media (SCM) has attracted interest for non-invasive PGT (niPGT)^16^. Studies using SCM to analyze chromosomal ploidy have reported positive and negative results. These studies reported relatively high amplification success rates (77.3–100%) using SCM, but consistency with whole embryos or TE varied greatly (33–100%)^17^. Additionally, studies on the clinical predictive value of niPGT-A have shown that it lacks precision and reliability as a diagnostic tool^18,19^.

To date, niPGT-A studies have primarily focused on analytical accuracy. Systematic reviews have suggested that high-quality evidence for the routine use of niPGT-A is insufficient, and well-designed, randomized, controlled trials (RCTs) are required to confirm its predictive capability on clinical outcomes^19^.

In this work, we conducted an investigator-initiated, multicenter, double-blind RCT to assess whether niPGT-A-selected frozen embryo transfers improve clinical outcomes compared with those based on the morphology score in women aged 35–42. Here, we show that niPGT-A does not significantly improve ongoing pregnancy rates, despite a reduction in early miscarriage. Our findings do not support routine niPGT-A use in this population.

## Results

### Study participants

**Fig. 1:**
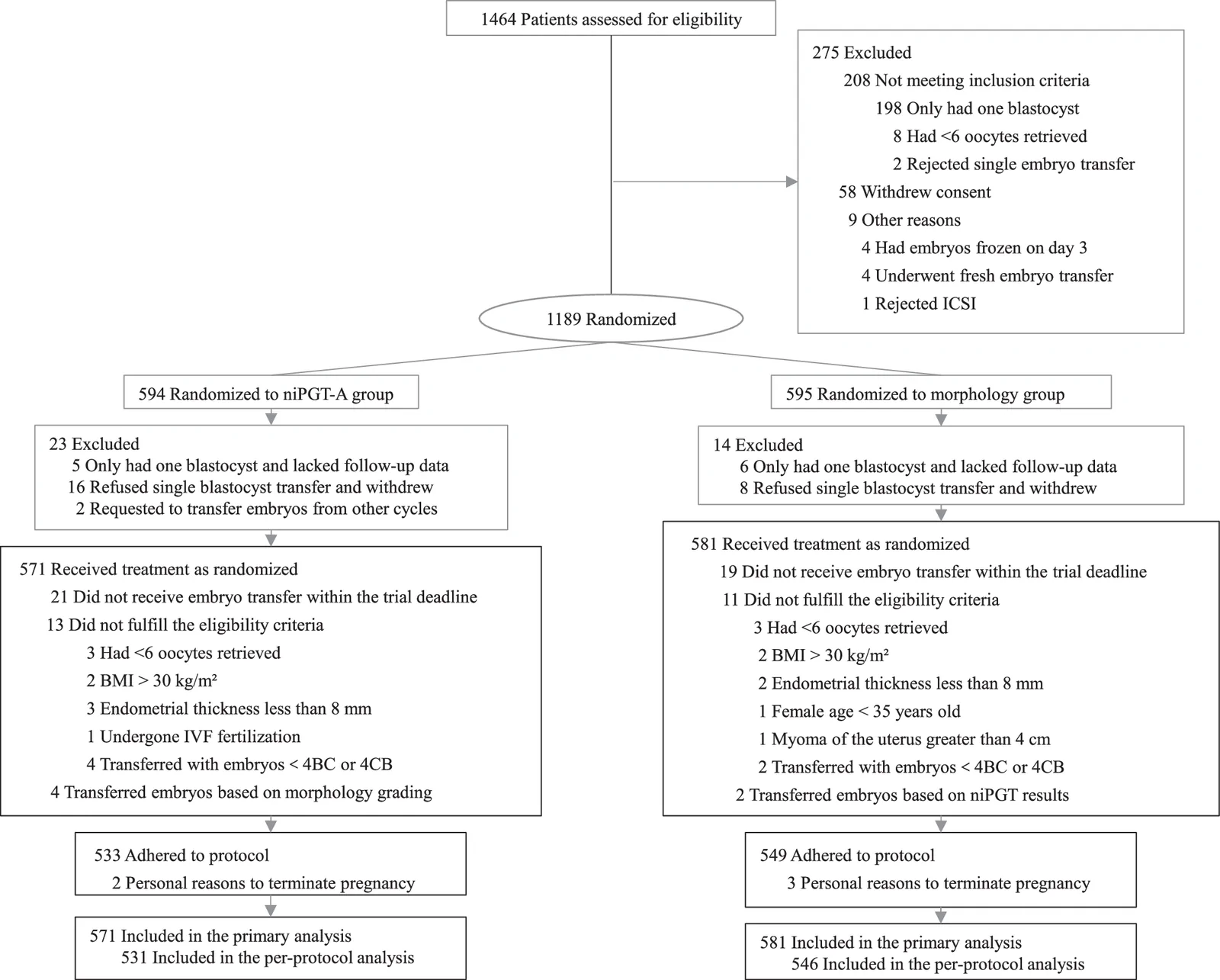
Patient flow in a trial of embryo selection using niPGT-A. *Among the 1189 randomized patients, a total of 37 patients were excluded in the primary analysis due to a lack of follow-up data or because they withdrew. The remaining 1152 patients received treatment as randomized and had follow-up data. After excluding patients who did not adhere to protocol, 40 patients (21 in the niPGT-A group and 19 in the morphology group) had not undergone embryo transfer at the end of the trial, 24 patients did not fulfill the eligibility criteria, 6 patients did not adhere to the principles of random grouping for embryo transfer, 5 patients terminated pregnancy due to personal reasons, and 1077 patients were included in the per-protocol analysis. For the mITT analysis, the 40 patients who had not undergone embryo transfer were coded as not achieving pregnancy for all outcomes.

Figure 1 shows the trial profile. A total of 1464 couples were screened between 16 April 2020 and 25 June 2023. Of these, 1189 women who met the inclusion criteria were randomly assigned to undergo their first embryo transfer according to niPGT-A grading (*n* = 594) or morphology grading (*n* = 595) (Fig. 1). We excluded couples who were found to be ineligible after randomization, including 11 participants who had only one blastocyst, which were grouped by mistake and did not have follow-up data, 24 patients who withdrew consent and requested to transfer 2 blastocysts, and 2 patients transferred embryos from other cycles. Finally, the modified intention-to-treat population(mITT) contained 581 couples in the morphology group and 571 in the niPGT-A group. The baseline characteristics of the participants were similar in the two groups (Table 1). The mean age of the 1152 female patients was 37.1 ± 2.0 years, and the mean BMI was 23.0 ± 3.0 kg/m². The mean blastocyst number was 4.6 in both groups, and 1112 (96.5%) participants underwent embryo transfer.

**Table 1:** Characteristics of the participants at baseline^*^

Characteristics	niPGT-A group (*n* = 571)	Morphology group (*n* = 581)
**Age, years**		
Female partner	37.2 ± 2.0	37.1 ± 1.9
35 no./total no. (%)	152/571 (26.6)	151/581 (26.0)
36–37 no./total no. (%)	205/571 (35.9)	217/581 (37.4)
38–39 no./total no. (%)	123/571 (21.5)	142/581 (24.4)
40–42 no./total no. (%)	91/571 (16.0)	71/581 (12.2)
Male partner	38.1 ± 4.9	38.0 ± 4.5
**Female partners’ BMI, kg/m**^2^	23.2 ± 3.1	22.9 ± 2.9
**Duration of infertility, years (IQR)**	3.0 (2.0, 6.0)	3.0 (2.0, 6.0)
**Number of previous embryo transfers, no./total no. (%)†**		
0–1	470/571 (82.3)	465/580 (80.2)
2	48/571 (8.4)	50/580 (8.6)
≥3	53/571 (9.3)	65/580 (11.2)
**Number of previous miscarriages, no./total no. (%)‡**		
0	442/571 (77.4)	455/580 (78.4)
1	91/571 (15.9)	100/580 (17.2)
≥2	38/571 (6.7)	25/580 (4.3)
**Previous live birth—yes, no. (%)**	202 (35.4)	188 (32.4)
**Primary infertility, no. (%)**	203 (35.6)	227 (39.1)
**Indication for IVF, no. (%)**		
Female factor	248 (43.4)	255 (43.9)
Male factor	144 (25.2)	147 (25.3)
Couples	179 (31.3)	179 (30.8)
**Laboratory testing (female partners)** **(IQR)**§		
Basic follicle-stimulating hormone, IU/L	5.9 (4.0, 7.3)	5.8 (4.2, 7.4)
Basic luteinizing hormone, IU/L	3.3 (1.9, 5.1)	3.4 (1.9, 4.8)
Basic estradiol, pmol/L	128.7 (84.0, 171.9)	129.7 (93.3, 179.4)
Basic progesterone, nmol/L	1.2 (0.8, 1.7)	1.2 (0.7, 1.9)
**Endometrial thickness, mm ¶**	10.9 ± 2.4	10.7 ± 2.2
**COH treatment**		
GnRH-agonist protocol, no. (%)	308 (53.9)	326 (56.1)
GnRH-antagonist protocol, no. (%)	198 (34.7)	183 (31.5)
Other, no. (%)	65 (11.4)	72 (12.4)
**No. of days of ovarian stimulation**	10.7 ± 2.1	10.5 ± 1.9
**The total dose of follicle-stimulating hormone - IU (IQR)**‖	2475.0 (1950.0, 3062.5)	2400.0 (1870.0, 3000.0)
Laboratory testing on the trigger day (IQR)**		
Luteinizing hormone, IU/L	1.6 (0.9, 2.8)	1.7 (0.8, 2.9)
Estradiol, pmol/L	12237.0 (7811.3, 18238.0)	12413.9 (7935.3, 18222.2)
Progesterone, nmol/L	3.0 (1.8, 4.7)	3.1 (1.9, 4.6)
**No. of oocytes retrieved**	15.2 ± 6.7	14.8 ± 6.7
**No. of good-quality embryos on day 3**	5.9 ± 3.6	5.8 ± 3.6
**No. of blastocysts**	4.6 ± 2.4	4.6 ± 2.4
**Undergo embryo transfer, no. (%)**	550 (96.3)	562 (96.7)
**Did not undergo embryo transfer, no. (%)**	21 (3.7)	19 (3.3)

^*^Categorical variables were summarized with frequencies and percentages. Normally distributed variables were presented as means and standard deviation (SD); non-normally distributed variables were presented as medians and interquartile ranges (IQR). In vitro fertilisation (IVF), controlled ovarian hyperstimulation (COH), gonadotropin-releasing hormone (GnRH), interquartile range (IQR)

†The number of previous transfers for one patient is unknown.

‡ Defined as pregnancy loss before 12 weeks. The number of previous miscarriages for one patient is unknown.

§Baseline steroid hormones were measured during the early follicular phase, mostly on days 1–3 of the menstrual cycle. Data were missing regarding follicle-stimulating hormone levels in 28 women in the niPGT-A group and 31 women in the morphology group, luteinizing hormone in 19 and 19 women, respectively, estradiol in 21 and 19 women, respectively, and progesterone levels in 35 women in each group.

¶Data on endometrial thickness were available for 569 women in the niPGT-A group and 578 women in the morphology group.

‖Data on the total dose of follicle-stimulating hormone were available for 570 women in the niPGT-A group and 580 women in the morphology group.

**Steroid hormone levels on human chorionic gonadotropin trigger day: Data on luteinizing hormone, estradiol, and progesterone in the niPGT-A group were available for 570, 569, and 570 women, respectively, and in the morphology group, 576, 575, and 574 women, respectively.

### Primary outcome

The primary outcome ongoing pregnancy (a pregnancy that continues beyond 12 weeks of gestation) rate after the first planned embryo transfer was 38.7% (221/571) in the niPGT-A group and 36.8% (214/581) in the morphology group (absolute difference, 1.9% [95% CI, −3.7% to 7.5%]; adjusted RR, 1.05 [95% CI, 0.91–1.22]; adjusted *P* = 0.49). The result did not demonstrate a significant improvement in ongoing pregnancy compared to morphology-based selection (Table 2). Similar results were observed in the per-protocol analysis (Supplementary Tables 1 and 2). The ongoing pregnancy rate was 41.2% (219/531) in the niPGT-A group and 37.9% (207/546) in the morphology group (absolute difference 3.3% [95% CI, −2.5% to 9.2%]; adjusted RR, 1.10 [95% CI, 0.95–1.27]; adjusted *P* = 0.23) (Supplementary Table 2).

**Table 2:** Primary and secondary outcomes after the planned first embryo transfer cycle (modified intention-to-treat)*

Outcome	NiPGT-A group (*n* = 571)	Morphology group (*n* = 581)	*P* value	RR (95% CI)	Absolute difference % (95% CI)	Adjusted *P* value^‡^	Adjusted RR (95% CI)
**Primary outcome**							
Ongoing pregnancy, no. (%)	221 (38.7)	214 (36.8)	0.51	1.05 (0.91, 1.22)	1.9 (−3.7, 7.5)	0.49	1.05 (0.91, 1.22)
**Secondary outcomes**							
Clinical pregnancy, no. (%)	272 (47.6)	290 (49.9)	0.44	0.95 (0.85, 1.07)	−2.3 (−8.1, 3.5)	0.49	0.96 (0.85, 1.08)
Miscarriage, no./total (%)	57/272 (21.0)	80/290 (27.6)	0.07	0.76 (0.57, 1.02)	−6.6 (−13.6, 0.5)	0.06	0.75 (0.56, 1.01)
Early miscarriages, no./total (%)	49/272 (18.0)	73/290 (25.2)	0.04	0.72 (0.52, 0.99)	−7.2 (−13.9, −0.4)	0.03	0.70 (0.51, 0.97)
Live birth, no. (%)†	211 (37.0)	206 (35.5)	0.60	1.04 (0.89, 1.22)	1.5 (−4.0, 7.0)	0.59	1.04 (0.90, 1.22)

Categorical variables were summarized with frequencies and percentages. Absolute differences in percentages are indicated in percentage points. All *P* values are two-sided. No adjustments were made for multiple comparisons. Chi-square test was used for categorical data.

*In niPGT-A group, there were 4 pregnancy cases that were neither live birth nor miscarriage, including 2 cases of personal reasons to terminate pregnancy before 12 weeks, 1 case of lost to follow-up after 12 weeks without live birth outcome, and 1 case of stillbirth due to umbilical cord torsion at 28 weeks. In morphology group, there were also 4 pregnancy cases that were neither live birth nor miscarriage, including 3 cases of personal reasons to terminate pregnancy before 12 weeks and 1 case of fetal loss due to traffic accident at 19 weeks.

†The numerator was the number of patients who had at least one live birth, and patients with live twins were counted as one.

‡Outcomes were calculated with a negative binomial regression model because the data were overdispersed.

### Secondary outcomes

Regarding the secondary outcomes, we found no statistically significant difference in the rates of clinical pregnancy (ultrasonographic confirmation of intrauterine gestation) (47.6% vs 49.9%; aRR 0.96 [95% CI, 0.85–1.08]; *P* = 0.49) or live birth (37.0% vs 35.5%; aRR 1.04 [95% CI, 0.90–1.22]; *P* = 0.59) between the two groups (Table 2). Miscarriage rate (pregnancy loss before the 28th week of pregnancy) was 21.0% in the niPGT-A group and 27.6% in the morphology group (aRR 0.75 [95% CI, 0.56–1.01]; *P* = 0.06); However, the rate of early miscarriage, referred to the spontaneous loss of an intrauterine pregnancy before 12 weeks of gestational, was lower in the niPGT-A group than in the morphology group (18.0% vs 25.2%; absolute difference, −7.2% [95% CI, −13.9% to −0.4%]; adjusted RR, 0.70 [95% CI, 0.51–0.97]; adjusted *P* = 0.03). The results of the per-protocol analysis were similar. The early miscarriage rate in the per-protocol analysis was significantly lower in the niPGT-A group than in the morphology group (17.7% vs 25.3%; absolute difference, −7.6% [95% CI, −14.5% to −0.7%]; adjusted RR, 0.68 [95% CI, 0.49–0.94]; adjusted *P* = 0.02). There was no statistically significant difference in the clinical pregnancy, miscarriage, or live birth rate between the groups (Supplementary Table 2).

### Safety outcomes

The risk of maternal complications, such as ectopic pregnancy, gestational diabetes, gestational hypertension, severe preeclampsia, premature rupture of membranes, and cervical incompetence, was similar between the niPGT-A and morphology groups (Table 3). Fetal and neonatal complications were also similar between the two groups (Table 3).

**Table 3:** Maternal and fetal/neonatal complications after the first planned embryo transfer cycle (modified intention-to-treat)

Complications	niPGT-A group (*N* = 571)	Morphology group (*N* = 581)	Absolute difference (95% CI)	Relative risk (95% CI)	*P* value
	No. of patients/total no. (%)		Percentage points		
**Maternal**					
Ectopic pregnancy	1/571 (0.2)	2/581 (0.3)	−0.1 (−1.1, 0.7)	0.51 (0.05, 5.60)	1.00
Gestational diabetes^*^	27/272 (9.9)	33/290 (11.4)	−2.2 (−7.3, 2.9)	0.81 (0.49, 1.32)	0.58
Gestational hypertension^*^	15/272 (5.5)	8/290 (2.8)	2.7 (−0.9, 6.0)	1.99 (0.86, 4.64)	0.10
Gestational diabetes mellitus combined with hypertension^*^	3/272 (1.1)	2/290 (0.7)	0.4 (−1.5, 2.6)	1.60 (0.27, 9.50)	0.68
Severe preeclampsia^*^	1/272 (0.4)	0	/	/	/
Premature rupture of membrane^*^	6/272 (2.2)	4/290 (1.4)	0.8 (−1.6, 3.5)	1.60 (0.46, 5.61)	0.67
Cervical incompetence ^*^	2/272 (0.7)	2/290 (0.7)	0.0 (−1.8, 2.0)	1.07 (0.15, 7.52)	1.00
Very preterm birth (28 ~ 34 weeks)^*^	8/272 (2.9)	5/290 (1.7)	1.2 (−1.5, 4.1)	1.71 (0.57, 5.15)	0.34
**Fetal/neonatal**					
Stillborn†	1	0	/	/	/
Congenital anomalies†	5/211 (2.4)	9/206 (4.4)	−2.0 (−5.5, 1.0)	0.54 (0.19, 1.59)	0.26
Very low birth weight( < 1500 g)^‡^	3/214 (1.4)	5/207 (2.4)	−1.0 (−1.9, 4.3)	0.58 (0.14, 2.40)	0.50
Macrosomia ( > 4000 g)^‡^	15/214 (7.0)	16/207 (7.7)	−0.7 (−5.0, 4.4)	0.91 (0.46, 1.79)	0.92

Categorical variables were summarized with frequencies and percentages. Absolute differences in percentages are indicated in percentage points. All *P* values are two-sided. No adjustments were made for multiple comparisons. A two-sample *t* test was used for continuous variables; a chi-square test was used for categorical data.

*Among clinical pregnancies.

†Among patients with live births.

‡Among all live newborns.

### Sensitivity analyzes

As sensitivity analyses, the results of the per-protocol analyses (Supplementary Tables 1–3) were consistent with mITT results. We performed a pre-specified sensitivity analysis based on the best-case (all interventions considered positive and control negative) and worst-case (all interventions considered negative and control positive) hypotheses regarding the distribution of missing primary outcomes. The effects of the interventions on the primary outcome were consistent across the two pre-specified analysis sets (Supplementary Tables 4 and 5). Furthermore, we undertook a completed cases analysis that excluded those who had not yet undergone embryo transfer at the end of the trial. The results were consistent with mITT results (Supplementary Table 6).

The results of additional endpoints for the core outcome set for infertility research, including both intention-to-treat analyses and per-protocol analyses, are presented in the appendix (Supplementary Tables 7 and 8).

### Exploratory subgroup analyses

**Fig. 2:**
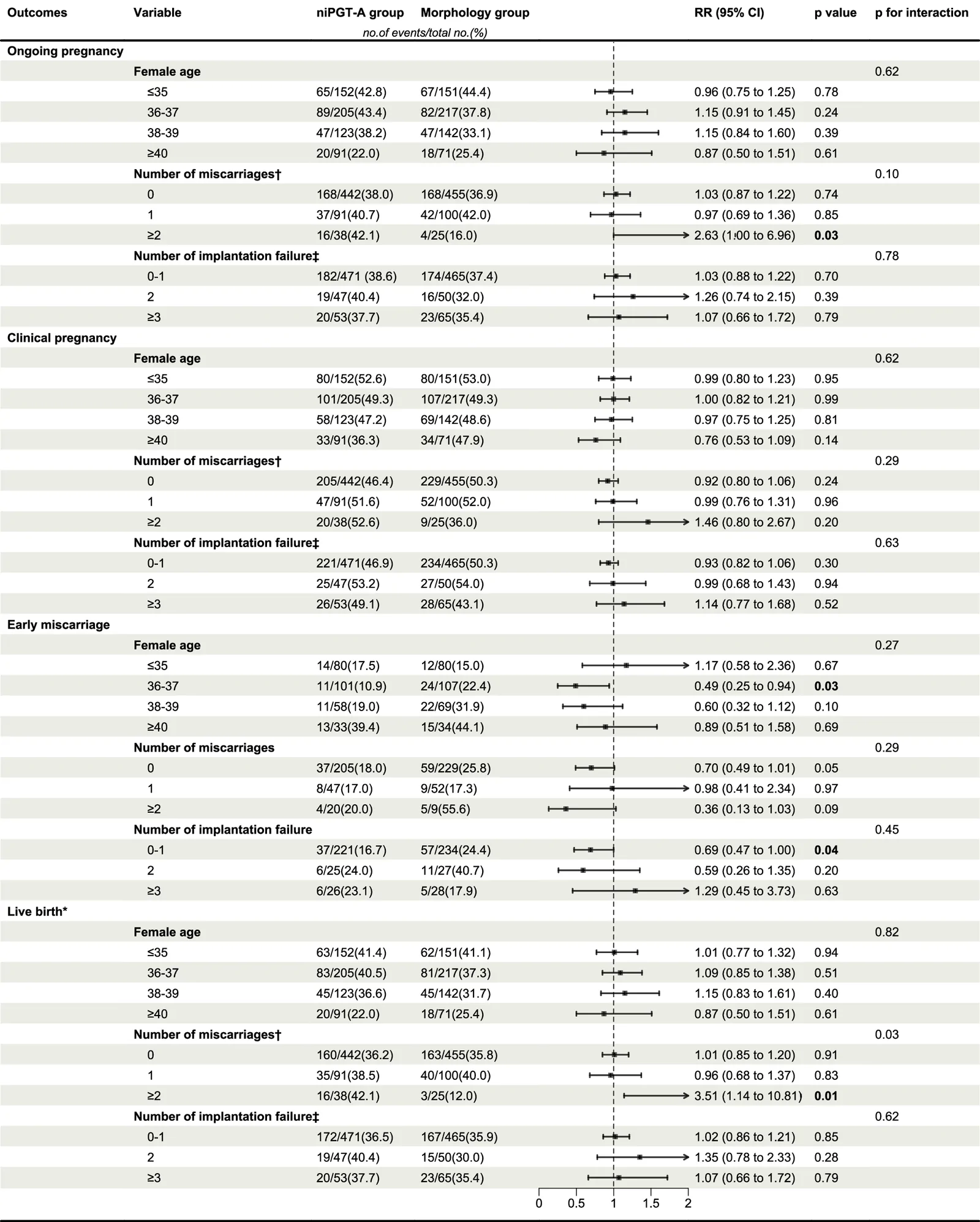
Subgroup analyses. RR relative risk. *The numerator was the number of patients with at least one live birth, and patients with live twins were counted as one. ^†^The number of previous transfers for one patient is unknown. ^‡^The number of miscarriages for one patient is unknown. Bold font indicates outcome categories, stratification variables, and statistically significant *P* values (*P* < 0.05). Italic font in the header indicates the number of events/total number (%). The filled black square represents the risk ratio (RR), and the error bars represent the 95% confidence interval. All *P* values are two-sided. No adjustments were made for multiple comparisons. Chi-square test or Fisher’s Exact Test was used for categorical data.

A subgroup analysis of female patients age showed that only in women aged 36–37, the early miscarriage rate was significantly lower in the niPGT-A group compared with that in the morphology group (10.9% vs 22.4%, *P* = 0.026). In this subgroup analysis, there were no significant differences in any of the other outcomes between the groups (Fig. 2).

When stratified by the number of previous miscarriages, no significant differences in the primary and secondary outcomes were observed in women with 0 or 1 previous miscarriage. When the number of previous miscarriages in a patient was ≥2, the ongoing pregnancy rate (*P* = 0.03) and live birth rate (*P* = 0.01) were significantly increased (Fig. 2).

Subgroup analysis based on the number of previous failed embryo transfers (0–1, 2, or ≥3 cycles) revealed that the benefit of niPGT-A in reducing early miscarriage was confined to women with 0–1 previous failures. Notably, for women with a history of 2 or more failed transfer cycles, no significant differences were observed between the niPGT-A and control groups (Fig. 2).

This analysis also showed no evidence of heterogeneity across the age and number of previously failed transfer subgroups, with all interaction *P* values ≥ 0.05. Notably, the effect of niPGT-A on live birth was heterogeneous across subgroups stratified by the number of previous miscarriages (*P* for interactio*n* = 0.03).

When stratified analysis in the niPGT-A group, a total of 550 blastocysts were transferred, including 92% of grade A, B blastocysts (506/550) and only 8% of grade C blastocysts (44/550). Statistically significant differences were observed between the blastocyst subgroup with NICS grades A + B and that with NICS grade C regarding clinical pregnancy rate, early miscarriage rate, ongoing pregnancy rate, and live birth rate (all *P* < 0.05). (Supplementary Table 9).

## Discussion

We conducted an investigator-initiated, multicenter, large-scale, double-blind RCT on niPGT-A. A novel strategy was introduced for ranking embryos for niPGT-A based on the probability of embryo euploidy and compared it with the traditional morphological score in patients with advanced maternal age. Embryo aneuploidy decreases the implantation rate and increases the miscarriage rate, while niPGT-A can be used to screen for chromosomal euploidy of the blastocyst. Therefore, an ongoing pregnancy was the primary outcome of this study. In this trial, only the clinical results of the first thawing transfer cycle were analyzed. NiPGT-A-based embryo selection did not significantly increase the ongoing pregnancy rate compared with the use of the morphological score. The rate of early miscarriage appeared to be lower in the niPGT-A group than in the morphological group. However, there was no significant difference in the rate of clinical pregnancy, overall miscarriage, or live birth between the two groups.

Over the past decade, studies on niPGT-A have primarily focused on the analytical accuracy and the concordance with the results of TE or the whole embryo^18,20–22^. The accuracy of niPGT-A has gradually improved during the last decade. Vera-Rodriguez et al.^20^ reported that the results of cfDNA in SCM differed from those of a trophectoderm biopsy analysis in 67% of cases in 2018. Rubio et al. conducted a multicenter, prospective, observational study. They analyzed 1301 human blastocysts from 8 reproductive centers and reported a concordance rate of 78.2% between niPGT-A and TE biopsy^23^. Moreover, based on the same study, Sakkas et al. reported that the implementation of an embryo culture protocol to accommodate niPGT-A did not affect blastocyst viability or pregnancy outcomes^24^. However, there is still a lack of robust evidence to determine whether niPGT-A can improve clinical pregnancy outcomes. Therefore, only studying the accuracy of cfDNA detection in SCM is insufficient. Consequently, the aim of our RCT was to determine whether niPGT-A is an effective tool for evaluating embryos.

Conventional PGT treatment cycles involve the disposal of aneuploid embryos. By comparison, none of the blastocysts in the current trial were disposed of. Instead, blastocysts in the niPGT-A group were prioritized for transfer based on their non-invasive chromosome screening (NICS) grade. Blastocysts were classified into 3 grades (A, B, and C) according to NICS results, with euploidy probabilities ≥0.94 for grade A, 0.7 to 0.94 for grade B, and ≤0.7 for grade C^25,26^. Moreover, in embryos with amplification failure or non-informative CNV results, the NICS grading system displayed null (N/A) results, which were assigned a grade of B in this study. Our recent study demonstrated that the embryos with amplification failure or non-informative CNV results achieved significantly higher live birth rates compared to those in the NICS-CNV group (42.3% vs 24.4%)^27^. This finding aligns with a smaller-scale study by Gary Nakhuda et al., where embryos with failed quality control (QC) (*n* = 9) exhibited higher sustained pregnancy rates than euploid embryos (66.7% vs. 57.3%), though the limited sample size warrants cautious interpretation^28^. Collectively, these data suggest that N/A-embryos maintain significant implantation potential, validating the rationale for their classification as grade B blastocysts. However, larger-scale studies are needed to definitively establish their position within embryo grading systems and to refine selection algorithms for optimal outcomes.

While cfDNA analysis in embryo culture media offers a non-invasive approach to assess the ploidy status of the corresponding embryo, two key points should be considered. First, the timing of media collection, to have enough cfDNA for an accurate diagnosis^29,30^. The development speed of blastocysts is not completely consistent. Our research has also demonstrated that the concentration of SCM cfDNA increased with the extension of embryo culture time^27^. Second is the contamination risk from non-embryonic DNA, which can confound results. Chen et al. revealed the origin of cfDNA in the culture medium^31^. The exogenous cfDNA originating from cumulus cells may be the biggest interfering factor in the accuracy of the ni-PGT. Optimized collection protocols and stringent contamination controls are essential to mitigate these challenges^32^.

Before this study began, the embryologists involved in the study were trained in a unified SCM sampling method. In this trial, all participants underwent ICSI. On the 3rd day, the residual cumulus cells were gently removed from the embryos with an appropriate stripper before blastocyst culture. On the 4th day, embryos were individually cultured in a new blastocyst culture medium. SCM was collected from Day 4 to the day the embryo developed to the blastocyst freezing standard. This does not interfere with the embryos’ process of IVF^33^. All 13 centers adhered to the same sampling protocol to minimize potential biases related to sampling procedures.

The SCM cfDNA content is extremely low, and the components are complex^31^. Therefore, at present, niPGT-A has not yet reached an accurate level. Moreover, there is self-correction during embryonic development. NiPGT-A results cannot determine the fate of a blastocyst. Therefore, in this study, we employed niPGT-A as a screening technique to rank embryos based on their probability of euploidy. All embryos were subsequently transferred. However, the embryo selection strategy using the specific niPGT-A protocol described in this study did not demonstrate superior ongoing pregnancy rates compared to conventional morphology-based selection. Future research should focus on 2 points. One point is to improve the accuracy of the SCM cfDNA detection technology, and the other is to further analyze which subgroup of people will benefit from niPGT-A.

Beyond technical limitations of the assay, another explanation for the negative outcomes between the two groups may be that the two methods would have selected embryos of the same quality. In the niPGT-A group, the embryo scheduled for transfer might have been the one with both the optimal NICS result and superior morphological grade. In the morphology group, the embryo planned for transfer was selected based on morphological assessment, which might have been a euploid embryo.

Our exploratory subgroup analysis showed that in patients with 2 or more previous miscarriages, the ongoing pregnancy and live birth rates appeared to be increased, and they may benefit from niPGT-A. Recurrent miscarriage is an indication for PGT-A because of the higher risk of aneuploid embryos in these patients. Sato et al. showed that PGT-A reduced pregnancy loss in patients with repeated pregnancy loss^34^. Therefore, niPGT-A may confer similar advantages to those with PGT-A by prioritizing euploid embryo transfers. In addition, although the effects of niPGT-A on clinical outcomes were consistent across the age and number of previously failed transfer subgroups, we observed a reduction in the early miscarriage rate, specifically in patients aged 36–37 years, and a tendency for a reduction in those aged 38–39 years (Fig. 2). For patients aged ≥40 years, we did not observe any differences, which may be attributed to the fact that embryo quality is only one of the factors affecting pregnancy outcomes. These patients are more likely to have additional factors, such as endometrial receptivity, contributing to pregnancy failure^35^. Patients with 2 or more previous failed transfer cycles showed no significant differences in clinical outcomes between the two groups, suggesting that their implantation failure involves more complex reasons beyond embryo selection. Notably, because these are exploratory findings, all results need to be confirmed in future trials with a larger sample size.

In this study, we observed a lower rate of early miscarriage in the niPGT-A group. Given that embryo aneuploidy is the main factor related to early miscarriage. A reduction in the early miscarriage rate in the niPGT-A group could be attributed to niPGT’s efficacy in identifying aneuploidy. Our results demonstrated superiority in reducing the early miscarriage rate but no statistically significant differences in primary outcomes. This discrepancy may be attributable to potential selection bias. Additionally, the denominator of the miscarriage rate was defined as the number of clinical pregnancies, which might have influenced the interpretation of the observed results. Moving forward, further research should both expand the sample size and focus on identifying which subgroup of patients will derive the greatest benefit from niPGT-A, thereby providing more targeted clinical guidance for its application in reproductive medicine.

The strengths of our RCT are that we had a large population size, a double-blind design, and a relatively small proportion of participants were lost to follow-up. In addition, there were only minor changes in the study protocol, including the addition of five reproductive centers and the cancellation of the time limit for the planned first embryo transfer. Although the primary outcome is negative, it is of great importance and will be critical for guiding the clinical application of niPGT-A.

This RCT also has some limitations. First, we included only women with two blastocysts and excluded those without a blastocyst or those with only one blastocyst. The participants had a relatively good prognosis. Therefore, the results may not be generalizable to women with a poor prognosis. Second, cumulative transfer data were not included. The cumulative pregnancy rate, which encompasses the outcomes of all embryo transfers in a single ovarian stimulation cycle, provides a more comprehensive measure of treatment success. Without these data, our study may not have fully determined the long-term efficacy of niPGT-A. Additionally, the time for SCM collection was not fixed. In this RCT, SCM collection was from day 4 to the day that the embryo developed to the blastocyst freezing standard. This time frame does not interfere with the embryos’ process of IVF because the development speed of blastocysts is not completely consistent. We previously found a correlation between cfDNA concentrations, the SCM collection date, and clinical outcomes^27^. Finally, in this study, the ongoing pregnancy rate was selected as the primary endpoint instead of the live birth rate. This decision was based on niPGT-A screens for aneuploid embryos, a leading cause of early miscarriage. The live birth rate was assessed as a secondary endpoint in the study, and the result was similar to the ongoing pregnancy.

In conclusion, this study shows that niPGT-A does not improve the ongoing pregnancy rate compared with that by morphological evaluation in women aged 35–42 years. The results indicate there was insufficient proof to establish a statistically significant difference between the ongoing pregnancy rates within the two treatment arms. At present, the findings do not support routine use of niPGT-A in this population with a good prognosis. Further research is required to improve the accuracy of niPGT-A and to identify what types of populations are more likely to benefit from niPGT-A.

## Methods

### Study design and setting

This study was an investigator-initiated, multicenter, double-blind RCT conducted across 13 reproductive centers in China. The research was performed in accordance with all relevant guidelines and regulations for the protection of human study participants. Written informed consent was obtained from all participants. The trial protocol and statistical analysis plan (SAP) received approval from the ethics committees of all sites. Lead approval was granted by the Medical Science Research Ethics Committee of Peking University Third Hospital (Ethics approval number: [2020] Medical Ethics Review No. 031-04). Ethical approval was obtained from the committees of all participating sites (including the Ethics Committee of Nanjing Jinling Hospital, the Medical Ethics Committee of Hebei Maternity Hospital, the Ethics Committee of Northwest Women’s and Children’s Hospital, the Ethics Committee of Chinese PLA General Hospital, the Ethics Committee of Shengjing Hospital of China Medical University, the Ethics Committee of the First Affiliated Hospital of Sun Yat-Sen University; the Ethical committee of the CITIC-Xiangya Reproductive & Genetic Hospital, the Ethics Committee of Peking University Shenzhen Hospital, the Ethics Committee of The Second Hospital of Hebei Medical University, the Ethics Committee of West China Second Hospital of Sichuan University, the Ethics Committee of the Second Affiliated Hospital of Wenzhou Medical University, the Ethics Committee of The Third Affiliated Hospital of Zhengzhou University). A data and safety monitoring board was established to oversee the trial. All data management and analyses were conducted at Peking University Third Hospital, which served as the data-coordinating center. The data in this trial were collected using a Web-based data management system. The trial protocol has been published previously^25^. The study protocol and SAP are available in the Supplementary Information (Supplementary Notes 2 and 3). The trial was registered at ClinicalTrials.gov, NCT04339166 (https://clinicaltrials.gov/study/NCT04339166).

### Study participants

We enrolled women with infertility who met the following inclusion criteria: (1) women aged 35–42 years; (2) body mass index (BMI) ranging from 18 to 30 kg/m²; (3) undergoing intracytoplasmic sperm injection (ICSI) procedures for an assisted reproduction cycle; (4) having retrieved at least 6 oocytes; and (5) having at least 2 blastocysts with a morphology score of >4BC or 4CB.

Women were excluded if they met any of the following conditions: (1) they had a contraindication for IVF or ICSI; (2) they underwent PGT cycles; (3) they had pathologies or malformations that affect pregnancy outcomes, such as genital malformations, untreated hydrosalpinx, untreated intrauterine infections, myoma of the uterus >4 cm, a benign tumor of the pelvic or abdominal cavity >4 cm, endometrial thickness <8 mm, pituitary tumors, and malignant tumors; (4) they had untreated hyperprolactinemia, thyroid disease, or adrenal disease; (5) they had endometrial polyps before the thawing transfer cycle.

Couples were counseled by local investigators at the office visit at the time of their decision to undergo ICSI treatment. The women were informed of the trial when their stimulation cycles were initiated. Written informed consent was obtained from each eligible participant before enrolment in the study. Randomization was implemented only after oocyte retrieval, specifically on day 5 or 6, upon confirmation of the availability of two or more transferable blastocysts. The study enrolled participants between 16 April 2020 and 25 June 2023. According to the study protocol, the planned overall study endpoint is set at 17 months post-oocyte retrieval of the last randomized subject. The trial completion endpoint for the entire clinical study was set for November, 2024. Actually, the last patient was randomized on June 25, 2023, and underwent the planned transfer on November 21, 2023. Follow-up for the final randomized patient and other participants’ delivery data was completed on August 6, 2024.

### Randomization and blinding

Eligible patients were randomized in a 1:1 ratio to the niPGT-A or morphology group. The randomization sequence was computer-generated. Blocked randomization (size 4 or 6) was implemented and stratified according to the trial center. Randomization was performed using an online trial management system. When eligible patients had 2 or more blastocysts, the laboratory staff performed multicenter randomization using the system and assigned random numbers accordingly. Researchers at each center conducted clinical treatment observations according to the assisted reproduction program linked to these random numbers. The random number served as the participant’s identifier throughout the clinical observation process, thus protecting the participant’s identity in reports of adverse events and other trial-related data.

This was a double-blind trial in which participants, clinicians, and nurses performing embryo transfers or follow-ups, as well as the investigators and assessors, were blinded to participants’ allocation until the primary outcome occurred. Only embryologists knew the allocation because they ranked the thawing blastocysts. Additionally, the staff at Yikon Genomics were blinded to the study.

### Procedures

Controlled ovarian hyperstimulation treatment was performed using standard routine procedures at each center. When at least two follicles reached a diameter of at least 18 mm, 250 µg human chorionic gonadotropin (hCG) was injected. Oocyte retrieval was arranged for 36 h ( ± 2) after hCG injection.

All participants underwent ICSI. The integrity and maturity of the denuded oocytes were examined. Enzymatic removal was performed during oocyte preparation. Only metaphase II oocytes were used for injection. Zygotes were cultured until day 3. The number and size of the blastomeres and anucleate fragmentation were scored. All centers used a sequential culture medium for blastocyst culture on day 3.

On the 3rd day, if cumulus cells still adhered to the oocytes, these residual cells were gently removed with an appropriate stripper before blastocyst culture. On the 4th day, embryos were individually cultured in a new blastocyst culture medium, with a volume of approximately 25 µl for each droplet. These procedures can effectively remove exogenous cell-free DNA (cfDNA) contamination and ensure the accuracy of the niPGT-A results. The blastocyst was vitrified when an embryo developed to the blastocyst freezing standard. All of the blastocysts were subjected to morphological assessment in accordance with the Gardner classification^36^. SCM was collected after removing the blastocyst from the culture drop to perform the vitrification procedure, and ~20 µl SCM from each corresponding blastocyst was collected through an RNase/DNase-free PCR tube. Moreover, 20 µL of media without any embryos (blank droplets) was incubated under the same conditions and collected and served as a negative control to evaluate the presence of any exogenous DNA contamination^25^. The embryologists involved in the study were trained in a unified SCM sampling method before the study began. All 13 centers adhered to the same sampling protocol to minimize potential biases related to sampling procedures.

SCM of all embryos in the two groups was collected and sent to Yikon Genomics (Suzhou, China) for testing using non-invasive chromosome screening (NICS) technology^26,37^. Whole-genome amplification and next-generation sequencing library preparation were performed on the collected SCMs using the NICSInst™ library kit (Xukang Medical Technology Co., Ltd., China). Pooled libraries were sequenced on the Illumina platform (Illumina Inc., USA), and approximately 2 M sequencing reads were obtained for each library. Copy number variations (CNVs) were performed according to established methodologies^25^, with a minimum resolution of 10 Mb. Briefly, high-quality reads were aligned to the genome in 1 Mb bins, followed by GC correction and normalization against control datasets. The circular binary segmentation (CBS) algorithm was then applied to detect CNV fragments. NICS was considered non-informative CNV when the sequencing data did not yield a reliable CNV profile, typically due to insufficient cfDNA, whole-genome amplification failure, or poor read quality. As described previously, we developed a machine learning-based embryo grading system employing a random forest algorithm^26^. This predictive model was trained using whole-embryo CNV profiles as the gold standard. Blastocysts were classified into three grades (A, B, and C) according to NICS results, with euploidy probabilities ≥0.94 for grade A, 0.7 to 0.94 for grade B, and ≤0.7 for grade C^25,26^. Moreover, in embryos with amplification failure or non-informative CNV results, the NICS grading system displayed null (N/A) results, which were assigned a grade of B in this study. All data were analyzed using ChromGo™ bioinformatics software (Xukang Medical Technology (Suzhou) Co., Ltd).

All participants underwent frozen–thawed embryo transfer. The intervention involved different embryo selection methods. In the morphological group, the embryo with the highest morphological score was selected. Embryo morphology ranking was as follows: 5AA > 5AB > 5BA > 4AA > 4AB > 4BA > 6AA > 6AB > 6BA > 5BB > 4BB > 6BB > 5AC > 5BC > 4AC > 4BC > 6AC > 6BC > 5CA > 5CB > 4CA > 4CB > 6CA > 6CB; and Day 5 >Day 6 >Day 7. This ranking was the consensus among the 13 centers participating in this trial. In the niPGT-A group, a single blastocyst was thawed and transferred in the order of A > B > C (NICS results) for the first transfer cycle. When blastocysts had the same NICS grade, those with a higher morphological grade were preferentially transferred.

Embryo transfer was performed by synchronization with the day 5 endometrium of a natural or hormonal replacement treatment cycle. Luteal phase support was provided according to the standards of each center. In this study, we focused on the first blastocyst thawing cycle. We followed up all conceptions until the end of pregnancy and obtained pregnancy and neonatal outcomes from obstetric and neonatal medical records.

### Outcomes

The primary outcome was ongoing pregnancy per the first planned blastocyst transfer, which was defined as a fetal heartbeat occurring 12 weeks after the blastocyst transfer. The ongoing pregnancy rate is defined as the number of women with an ongoing pregnancy divided by the number of women randomized to each group. Secondary outcomes included clinical pregnancy, miscarriage, and live birth. A clinical pregnancy was defined as the identification of an intrauterine gestational sac via ultrasound. Miscarriage was defined as a pregnancy loss that occurred before 28 weeks. Early miscarriage was defined as the spontaneous loss of an intrauterine pregnancy before 12 weeks. The miscarriage rate was defined as the number of women with miscarriage divided by the number of clinical pregnancy women. Live birth was defined as the delivery of any viable neonate. All serious adverse events and protocol violations were reported according to standard procedures. The primary outcome and all secondary outcomes were observed for the first embryo transfer. The collection of maternal and neonatal information was completed within 2 weeks of delivery.

### Sample size calculation

This is a superiority trial, aiming to find a 10% higher ongoing pregnancy rate in the niPGT-A arm. Assuming a 38.8% in the morphology group, a 90% power, and a two-sided significance level of 0.05, a minimum sample of 516 patients in each treatment group is required. We estimated a group sample size of 574 participants per arm to account for a dropout rate of 10%.

### Statistical analysis

The primary and secondary outcomes analysis was conducted according to the modified intention-to-treat (mITT) principle. The modified intention-to-treat population referred to all randomly assigned participants who experienced at least one intervention and had post-intervention evaluation data, except for those who were ineligible or withdrew consent. We compared the primary outcome and secondary outcomes between groups using the Pearson *χ*2 test or Fisher’s exact test as appropriate. The relative risk (RR) and 95% confidence interval (CI) were determined. Outcomes between groups were also compared using Poisson regression with robust SEs, adjusting for the center, and presented as adjusted RRs and corresponding 95% CIs according to the revised protocol (Version 2.0). If the data were overdispersed, a negative binomial regression was conducted. For patients who did not undergo embryo transfer (*n* = 40), all pregnancy-related outcomes were considered negative. For patients with partial loss to follow-up after embryo transfer, missing outcomes were coded as negative (*n* = 1).

A secondary per-protocol analysis was performed in the participants who adhered to the trial protocol. Participants who had not yet undergone embryo transfer at the end of the trial, those who were grouped but did not meet the inclusion criteria, those who did not adhere to the principles of random grouping for embryo transfer, and those who chose to terminate their pregnancies for personal reasons were excluded from the per-protocol analysis.

A pre-specified sensitivity analysis was performed to evaluate the best-case and worst-case hypotheses regarding the distribution of missing values. In the best-case hypothesis, the missing outcomes of patients in the morphology group were considered negative, whereas those in the niPGT-A group were assigned as live births. In the worst-case hypothesis, the missing outcomes of patients in the morphology group were considered live births, while those in the niPGT-A group were considered negative. Furthermore, we performed a non-pre-specified sensitivity analysis by completing cases, excluding those who had not yet undergone embryo transfer at the end of the trial.

Our statistical analysis incorporated several additional exploratory analyses not pre-specified in the original protocol. Subgroup analyses were conducted according to women age, the number of previous miscarriages, and the number of prior failed embryo transfer cycles to examine whether a particular group would benefit from niPGT-A. In the subgroup analyses, we examined women age (35 years group, 36–37 years group, 38–39 years group, and 40–42 years group), the number of previous miscarriages (0, 1, and ≥2), and the number of prior failed embryo transfer cycles (0–1, 2, and ≥3). All analyses were performed using an mITT population. In addition, the core outcome set was analyzed on the basis of mITT and per-protocol analyses, according to the core outcome set for infertility research published after the start of our study^35^. The trial data were monitored by an independent data monitoring committee.

All analyses were performed using SPSS version 25.0 (IBM SPSS Inc.). R software (version 4.3.3) was used for generating forest plots. A *P* value < 0.05 was considered to indicate statistical significance.

### Reporting summary

Further information on research design is available in the Nature Portfolio Reporting Summary linked to this article.

## Supplementary information

Supplementary Information

Reporting Summary

Transparent Peer Review file

## Source data

Source Data_revised

## Data Availability

Our study is compliant with the Guidance of the Ministry of Science and Technology (MOST) of China for the Review and Approval of Human Genetic Resources. The sequencing data have been deposited in the Genome Sequence Archive (GSA) for Human under accession code HRA020349. The data are available under controlled access due to patient privacy restrictions. Access can be requested via the Data Access Committee (DAC) through the provided link. The trial’s supporting documents, including the study protocol and the statistical analysis plan, have been compiled in the supplementary information. De-identified individual data underlying the results reported in this study, including specified datasets together with a data dictionary defining each field in the set, will be made available to other researchers on request by emailing the project lead investigator (jie.qiao@263.net). To facilitate this process, a formal proposal outlining the study’s aims, the planned statistical analyses, publication plans, and distribution strategies is required. Requests will be reviewed and responded to within 8 weeks of submission. The data-sharing request will be reviewed and approved by the principal investigator, the trial’s management team, and the Medical Science Research Ethics Committee of Peking University Third Hospital, ensuring compliance with relevant laws and regulations in China. The remaining data are available within the Article, Supplementary Information, or Source Data file. Source data are provided with this paper.

## Code availability

No custom code was used for statistical analysis in this study.
