# Supplementary material for: Embryo selection by non-invasive preimplantation genetic testing for aneuploidy in women of advanced maternal age: a randomized, clinical trial
Source: Nat Commun. 2026 Sep 8;17:10634. doi: 10.1038/s41467-026-77628-4 (PMC13650144; doi:10.1038/s41467-026-77628-4)
Supplement: Supplementary file 1 [file 41467_2026_77628_MOESM1_ESM.pdf]

Supplementary Information

Embryo Selection by Non-invasive Preimplantation Genetic Testing for Aneuploidy in Women of Advanced Maternal Age: A Randomized, Clinical Trial

Jin Huang†, Rong Li†, Lin Zeng†, Liang Hu†, Liyi Cai†, Li Chen†, Juanzi Shi, Yan Wang, Yichun Guan, Yanwen Xu, Weiping Qian, Xiuxia Wang, Haitao Xi, Zhiming Zhao, Yuanqing Yao, Sijia Lu, Yaxin Yao, Jing Wang, Ping Liu*, Jie Qiao*

† These authors contributed equally: Jin Huang, Rong Li, Lin Zeng, Liang Hu, Liyi Cai, Li Chen

*Corresponding authors: Prof Jie Qiao(jie.qiao@263.net) or Ping Liu (pingliu7703@sina.com)

Supplementary Tables

Supplementary Table 1. Baseline Characteristics (Per-Protocol Analysis)

Supplementary Table 2. Primary and Secondary Outcomes after the First Planned Embryo Transfer Cycle (Per-Protocol Analysis)

Supplementary Table 3. Maternal and Foetal/Neonatal Complications after the First Planned Embryo Transfer Cycle (Per-Protocol Analysis)

Supplementary Table 4. Primary and Secondary Outcomes after the First Planned Embryo Transfer Cycle (Sensitivity Analysis-Best Case Imputation)

Supplementary Table 5. Primary and Secondary Outcomes after the First Planned Embryo Transfer Cycle (Sensitivity Analysis-Worst Case Imputation)

Supplementary Table 6. Primary and Secondary Outcomes after the First Planned Embryo Transfer Cycle (Complete Case Analysis)

Supplementary Table 7. Core Outcomes According to the Standardizing Definitions and Reporting Guidelines for the Infertility Core Outcome Set (modified Intention-to-Treat Analysis)

Supplementary Table 8. Core Outcomes According to the Standardizing Definitions and Reporting Guidelines for the Infertility Core Outcome Set (Per-Protocol Analysis

Supplementary Table 9. Stratified Analysis by NICS Results

Supplementary Table 1. Baseline Characteristics (Per-Protocol Analysis) *

Characteristics	niPGT-A group (n=531)	Morphology group (n=546)
**Age, years**		
Female	37.1± 2.0	37.1±1.9
Male	38.0±4.8	38.0±4.5
**Female Body mass index – kg/m^2**	23.2±3.0	22.9±2.9
**Duration of infertility – years**	3.0(2.0,6.0)	3.0(2.0,6.0)
**Number of previous embryo transfers, no (%) †**		
0-1	442 (83.2)	437 (80.0)
2	39 (7.3)	47 (8.6)
≥3	50 (9.4)	61 (11.2)
**Number of previous miscarriages, no (%) †**		
0	414 (78.0)	430 (78.9)
1	81 (15.3)	91 (16.7)
≥2	36 (6.8)	24 (4.4)
**Previous live birth-yes, no. (%)**	185 (34.8)	176 (32.2)
**Primary infertility, no. (%)**	193 (36.4)	219 (40.1)
**Indication for IVF, no. (%)**		
Female factor	230 (43.3)	236 (43.2)
Male factor	138 (26.0)	139 (25.5)
Couple	163 (30.7)	171 (31.3)
**Laboratory testing (Female)‡**		
Basic follicle-stimulating hormone — IU/L	5.8(3.9,7.3)	5.8(4.2-7.3)
Basic luteinizing hormone — IU/L	3.3(1.9, 5.1)	3.4(2.0-4.8)
Basic estradiol — pmol/L	128.5(83.4,171.0)	129.0(92.0, 177.3)
Basic progesterone — nmol/L	1.2(0.7,1.6)	1.2(0.7,1.8)
**Endometrial thickness — mm§**	11.0±2.4	10.9±2.1
**COH treatment**		
GnRH-a protocol, **no. (%)**	291 (54.8)	311 (57.0)
GnRH-ant protocol, **no. (%)**	183 (34.5)	169 (31.0)
Other, **no. (%)**	57 (10.7)	66 (12.1)
**No. of days of ovarian stimulation**	10.8±2.1	10.5±1.9
**The total dose of follicle-stimulating hormone - IU¶**	2475.0(1950.0,3009.4)	2381.3(1837.5,3000.0)
**Laboratory testing on the trigger day‖**		
Luteinizing hormone — IU/L	1.7(0.9,3.0)	1.6(0.9,2.9)
Estradiol — pmol/L	12372.0(7981.9,18377.5)	12560.0(8041.0,18449.1)
Progesterone — nmol/L	3.0(1.8,4.7)	3.1(1.9,4,6)
**No. of oocytes retrieved**	15.5±6.7	15.0±6.7
**No. of good-quality embryos on day 3**	6.1± 3.6	5.9± 3.6
**No. of blastocysts**	4.7±2.4	4.6 ± 2.4

* Normally distributed variables were presented as means and standard deviation (SD); non-normally distributed variables were presented as medians and interquartile ranges (IQR). In vitro fertilisation (IVF), controlled ovarian hyperstimulation (COH), gonadotropin-releasing hormone (GnRH), interquartile range (IQR)

† One patient in the morphology group had an unknown number of previous transfers and previous miscarriages.

‡ Baseline steroid hormones were measured during the early follicular phase, mostly on days 1–3 of the menstrual cycle. Data were missing for follicle-stimulating hormone in 21 women in the niPGT-A group and 29 in the morphology group; luteinizing hormone in 15 women and 18 women, respectively; regarding oestradiol in 17 women and 18 women, respectively; and progesterone in 30 women and 33 women, respectively.

§ Data on endometrial thickness were available for 529 women in the niPGT-A group and 543 women in the morphology group.

¶ Data on the total dose of follicle-stimulating hormone were available for 530 women in the niPGT-A group and 545 women in the morphology group.

‖ Steroid hormone levels on human chorionic gonadotropin trigger day: Data on luteinizing hormone, oestradiol, and progesterone in the niPGT-A group were available for 531, 529, and 531 women, respectively, and in the morphology group, 541, 540, and 539 women, respectively.

Supplementary Table 2. Primary and Secondary Outcomes after the First Planned Embryo Transfer Cycle (Per-Protocol Analysis)*

Outcome	NiPGT-A group (n=531)	Morphology group (n=546)	P-value	Relative Risk (95% CI)	Absolute difference (95% CI)	Adjusted p-value‡	Adjusted RR (95% CI)
**Primary outcome**							
Ongoing pregnancy, no. (%)	219 (41.2)	207 (37.9)	0.26	1.09(0.94,1.26)	3.3(-2.5,9.2)	0.23	1.10(0.95,1.27)
**Secondary outcomes**							
Clinical pregnancy, no. (%)	266 (50.1)	277 (50.7)	0.83	0.99(0.88,1.11)	-0.6(-6.6, 5.3)	0.97	1.00 (0.89,1.12)
Miscarriage, no./total (%)	55/266(20.7)	76/277(27.4)	0.07	0.75(0.56,1.02)	-6.7(-13.9, 0.5)	0.05	0.74(0.55,1.00)
Early miscarriages, no./total (%)	47/266 (17.7)	70/277 (25.3)	0.03	0.70(0.50,0.97)	-7.6(-14.5, -0.7)	0.02	0.68(0.49,0.94)
Live birth, no. (%) †	209(39.4)	200(36.6)	0.36	1.07(0.92,1.25)	2.8(-3.1,8.5)	0.32	1.08(0.93,1.26)

Values are presented as n (%). Absolute differences in percentages are indicated in percentage points. All *p* values are two-sided. No adjustments were made for multiple comparisons. Chi-square test was used for categorical data.

*In niPGT-A group, there were 2 pregnancy cases that were neither live birth nor miscarriage, including 1 case of lost to follow-up after 12 weeks without live birth outcome, and 1 case of stillbirth due to umbilical cord torsion at 28 weeks.

In morphology group, there was 1 case of fetal loss due to a traffic accident at 19 weeks that was neither live birth nor miscarriage.

† The numerator was the number of patients who had at least one live birth, and patients with live twins were counted as one.

‡ Calculated with a Negative binomial regression model because the data were overdispersed.

Supplementary Table 3. Maternal and Foetal/Neonatal Complications after the First Planned Embryo Transfer Cycle (Per-Protocol Analysis)

Complications	NiPGT-A group (n=531)	Morphology group (n=546)	Absolute difference (95% CI)	Relative Risk (95%CI)	P value
	No. of patients/total no. (%)		percentage points		
Maternal					
Ectopic pregnancy	1/531(0.2)	2/546(0.4)	-0.2(-1.2,0.7)	0.51(0.05-5.65)	1.00
Gestational diabetes*	27/266(10.2)	32/277(11.6)	-1.4(-6.7,3.9)	0.88(0.54-1.43)	0.60
Gestational hypertension*	15/266(5.6)	8/277(2.9)	2.7(-0.7,6.5)	1.95(0.84-4.53)	0.11
Gestational diabetes mellitus combined with hypertension*	3/266(1.1)	2/277(0.7)	0.4(-1.6,2.6)	1.56(0.26-9.27)	0.96
Severe preeclampsia	1	0			
Premature rupture of membranes*	6/266(2.3)	3/277(1.1)	1.2(-1.2,3.9)	2.08(0.53-8.24)	0.46

Cervical incompetence *	2/266(0.7)	2/277(0.7)	0.0(-1.9,2.0)	1.04(0.15-7.34)	1.00
Very preterm birth (28~34 weeks)*	8/266(3.0)	5/277(1.8)	1.2(-1.6,4.2)	1.67(0.55-5.03)	0.36
Foetal/neonatal					
Stillborn	1	0			
Congenital anomalies†:	5/209(2.4)	9/200(4.5)	-2.1(-6.2,1.6)	0.53(0.18-1.56)	0.24
Very low birth weight(<1500g)‡:	3/212(1.4)	5/201(2.5)	-1.1(-4.4,1.9)	0.57(0.14-2.35)	0.67
Macrosomia (>4000g)‡:	14/212(6.6)	16/201(8.0)	-1.4(-6.6,3.8)	0.83(0.42-1.66)	0.60

Values are presented as n (%). Absolute differences in percentages are indicated in percentage points. All *p* values are two-sided. No adjustments were made for multiple comparisons. Chi-square test was used for categorical data.
*: Among clinical pregnancies.
†: Among patients with live births.
‡: Among all live newborns.

Supplementary Table 4. Primary and Secondary Outcomes after the First Planned Embryo Transfer Cycle (Sensitivity Analysis-Best Case Imputation)

Outcome	NiPGT-A group (n=571)	Morphology group (n=581)	P-value	Relative Risk (95% CI)	Absolute difference (95% CI)
**Primary outcome**					
Ongoing pregnancy, no. (%)	242 (42.4)	214 (36.8)	0.05	1.15(1.00,1.33)	5.6(-0.1, 11.2)
**Secondary outcomes**					
Clinical pregnancy, no. (%)	293 (51.3)	290 (49.9)	0.64	1.03(0.92,1.15)	1.4 (-4.4, 7.2)
Miscarriages, no./total (%)	57/293(19.5)	80/290(27.6)	0.02	0.71(0.52,0.95)	-8.1(-14.9, -1.2)
Early miscarriages, no./total (%)	49/293(16.7)	73/290(25.2)	0.01	0.66(0.48,0.92)	-8.5(-15.0, -1.8)
Live birth, no. (%)	233(40.8)	206(35.5)	0.06	1.15(0.99,1.33)	5.4(-0.3,10.9)

Values are presented as n (%). Absolute differences in percentages are indicated in percentage points. All *p* values are two-sided. No adjustments were made for multiple comparisons. Chi-square test was used for categorical data.

Supplementary Table 5. Primary and Secondary Outcomes after the First Planned Embryo Transfer Cycle (Sensitivity Analysis-Worst Case Imputation)

Outcome	niPGT-A group (n=571)	Morphology group (n=581)	p-value	Relative Risk (95% CI)	Absolute difference (95% CI)
**Primary outcome**					
Ongoing pregnancy, no. (%)	221 (38.7)	233 (40.1)	0.63	0.97(0.84,1.11)	-1.4(-7.0, 4.2)
**Secondary outcomes**					
Clinical pregnancy, no. (%)	272 (47.6)	309 (53.2)	0.06	0.90(0.80,1.01)	-5.6 (-11.3, 0.2)
Miscarriages, no./total (%)	57/272(21.0)	80/309(25.9)	0.16	0.81(0.60,1.09)	-4.9(-11.7,2.0)
Early miscarriages, no./total (%)	49/272(18.0)	73/309(23.6)	0.10	0.76(0.55,1.05)	-5.6(-12.1,1.1)
Live birth, no. (%)	211(37.0)	225(38.7)	0.54	0.95(0.82,1.11)	-1.7(-7.4,3.8)

Values are presented as n (%). Absolute differences in percentages are indicated in percentage points. All *p* values are two-sided. No adjustments were made for multiple comparisons. Chi-square test was used for categorical data.

Supplementary Table 6. Primary and Secondary Outcomes after the First Planned Embryo Transfer Cycle (Complete Case Analysis)

Outcome	NiPGT-A group (n=550)	Morphology group （n=562）	p-value	Relative Risk (95% CI)	Absolute difference (95% CI)
**Primary outcome**					
Ongoing pregnancy, no. (%)	221 (40.2)	214 (38.1)	0.47	1.06(0.91,1.22)	2.1(-3.6, 7.8)
**Secondary outcomes**					
Clinical pregnancy, no. (%)	272 (49.5)	290 (51.6)	0.47	0.96(0.85,1.08)	-2.1(-8.0, 3.7)
Miscarriage, no./total (%)	57/272(21.0)	80/290(27.6)	0.07	0.76(0.57,1.02)	-6.6(-13.6, 0.5)
Early miscarriages, no./total (%)	49/272(18.0)	73/290(25.2)	0.04	0.72(0.52,0.99)	-7.2(-13.9, -0.4)
Live birth, no. (%)	211(38.4)	206(36.7)	0.56	1.05(0.90,1.22)	1.7(-4.0,7.4)

Values are presented as n (%). Absolute differences in percentages are indicated in percentage points. All *p* values are two-sided. No adjustments were made for multiple comparisons. Chi-square test was used for categorical data.

Supplementary Table 7. Core Outcomes According to the Standardizing Definitions and Reporting Guidelines for the Infertility Core Outcome Set (modified Intention-to-Treat Analysis)

Outcome	niPGT-A group (n=571)	Morphology group (n=581)	p-value‡	Relative Risk (95% CI)	Absolute difference (95% CI)
**Live birth event, no. (%)**	211(37.0)	206(35.5)	0.60	1.04(0.89,1.22)	1.5(-4.0,7.0)
Singleton, no. (%)	207(36.3)	205(35.3)	0.73	1.04(0.88,1.20)	1.0(-4.6,6.5)
Twin, no. (%)	4(0.7)	1(0.2)	0.36	4.07(0.46,36.30)	0.5(-0.4,1.6)
**Viable pregnancy confirmed by ultrasound, no. (%)**	272 (47.6)	290 (49.9)	0.44	0.95(0.85,1.07)	-2.3 (-8.1, 3.5)
Singleton pregnancy, no. (%)	266(46.6)	286(49.2)	0.37	0.95(0.84,1.07)	-2.6(-8.4,3.1)
Twin pregnancy, no. (%)	6(1.0)	4(0.7)	0.73	1.53(0.43,5.38)	0.3(-0.9,1.7)
**Pregnancy loss**					
Ectopic pregnancy, no.	1	2			
Miscarriage, no.	57	80			
Stillbirth, no*	1	0			
Termination of pregnancy, no.	2	4			
**Lost to follow-up**	1	0			
**Gestational age at delivery (weeks of gestation), mean (SD)**	38.1(±2.1)	38.3(±2.0)	0.19		
**Birthweight**					
Singleton, g. (mean, SD) †	3314.2(±590.3)	3353.5(±523.3)	0.48		
Twin, g. (mean, SD) †	2180.0(±424.3)	1000.0(±615.0)	0.07		
**Major congenital anomaly, no.**	5	9			

Values are presented as mean ± SD or n (%). Absolute differences in percentages are indicated in percentage points, and absolute differences in other values are indicated in units of that value.

*: One patient had stillbirth due to umbilical cord torsion at 28 weeks

†: For continuous outcomes, the p-value was reported by using a t-test. The sample size for birth weight was 215 newborns (211 couples) in the niPGT-A group and 207 newborns (206 couples) in the morphology group, including 207 newborns (207 couples) and 205 newborns (205 couples) for singleton, 8 newborns (4 couples) and 2 newborns (1 couple) for twin, respectively.

‡: All *p* values are two-sided. No adjustments were made for multiple comparisons. Two-sample t-test was used for continuous variables; Chi-square test was used for categorical data.

Supplementary Table 8. Core Outcomes According to the Standardizing Definitions and Reporting Guidelines for the Infertility Core Outcome Set (Per-Protocol Analysis)

Outcome	NiPGT-A group (n=531)	Morphology group (n=546)	p-value‡	Relative Risk (95% CI)	Absolute difference (95% CI)
**Live birth event, no. (%)**	209(39.4)	200(36.6)	0.36	1.07(0.99,1.25)	2.8(-3.1,8.5)
Singleton, no. (%)	205(38.6)	199(36.4)	0.46	1.06(0.91,1.24)	2.2(-3.6,7.9)
Twin, no. (%)	4(0.8)	1(0.2)	0.35	4.11(0.46,36.68)	0.6(-0.4,1.8)
**Viable pregnancy confirmed by ultrasound, no. (%)**	266 (50.1)	277 (50.7)	0.83	0.99(0.88,1.11)	-0.6(-6.6, 5.3)
Singleton pregnancy, no. (%)	260(49.0)	273(50.0)	0.73	0.98(0.87,1.11)	-1.0(-7.0,4.9)
Twin pregnancy, no. (%)	6(1.1)	4(0.7)	0.72	1.54(0.44,5.44)	0.4(-0.9,1.8)
**Pregnancy loss**					
Ectopic pregnancy, no.	1	2			
Miscarriage, no.	55	76			
Stillbirth, no*	1	0			
Termination of pregnancy, no.	0	1			
**Lost to follow-up**	1	0			
**Gestational age at delivery (weeks of gestation), mean (SD)**	38.1(±2.1)	38.3(±2.0)	0.19		
**Birthweight**					
Singleton, g. (mean, SD) †	3310.5(±590.2)	3355.8(±529.1)	0.42		
Twin, g. (mean, SD) †	2180.0(±424.3)	1000.0(±615.0)	0.07		
**Major congenital anomaly, no.**	5	9			

Values are presented as mean ± SD or n (%). Absolute differences in percentages are indicated in percentage points, and absolute differences in other values are indicated in units of that value.

*: One patient had stillbirth due to umbilical cord torsion at 28 weeks.

†: For continuous outcomes, *p*-value was reported using a t-test. The sample size for birth weight was 213 newborns (209 couples) in the niPGT-A group and 201 newborns (200 couples) in the morphology group, including 205 newborns (205 couples) and 199 newborns (199 couples) for singleton, 8 newborns (4 couples) and 2 newborns (1 couple) for twin, respectively.

‡: All *p* values are two-sided. No adjustments were made for multiple comparisons. Two-sample t-test was used for continuous variables; Chi-square test was used for categorical data.

Supplementary Table 9. Stratified Analysis by NICS Results

Subgroups*	Clinical pregnancy	Early miscarriage	Ongoing pregnancy	Live birth
A+B	262/506(51.8)	44/262(16.8)	216/506(42.7)	206/506(40.7)
C	10/44(22.7)	5/10(50.0)	5/44(11.4)	5/44(11.4)
Morphology	290/562(51.6)	73/290(25.2)	214/562(38.1)	206/562(36.7)
p-value (A +B vs Morphology)	0.95	0.02	0.13	0.17
p-value (C vs Morphology)	0.0002	0.16	0.0004	0.0007
p-value (A+B vs C)	0.0002	0.02	0.00005	0.0001

*According to the NICS results, blastocysts were classified into three grades: grade A (euploidy probability ≥0.94), grade B (0.70–0.94), and grade C (≤0.70). All *p* values are two-sided. No adjustments were made for multiple comparisons. Chi-square test was used for categorical data.

Supplementary Notes

Supplementary Note 1. CONSORT checklist
Supplementary Note 2. Trial protocol
Supplementary Note 3. Statistics analysis plan

CONSORT 2010 checklist of information to include when reporting a randomised trial*

Section/Topic	Item No	Checklist item	Reported on page No
**Title and abstract**			
	1a	Identification as a randomised trial in the title	1
	1b	Structured summary of trial design, methods, results, and conclusions (for specific guidance see CONSORT for abstracts)	3
**Introduction**			
Background and objectives	2a	Scientific background and explanation of rationale	4,5
	2b	Specific objectives or hypotheses	5
**Methods**			
Trial design	3a	Description of trial design (such as parallel, factorial) including allocation ratio	15,16
	3b	Important changes to methods after trial commencement (such as eligibility criteria), with reasons	No important changes to methods after trial commencement
Participants	4a	Eligibility criteria for participants	16,17
	4b	Settings and locations where the data were collected	18
Interventions	5	The interventions for each group with sufficient details to allow replication, including how and when they were actually administered	19,20
Outcomes	6a	Completely defined pre-specified primary and secondary outcome measures, including how and when they were assessed	21
	6b	Any changes to trial outcomes after the trial commenced, with reasons	No changes to trial outcomes after the trial commenced
Sample size	7a	How sample size was determined	21
	7b	When applicable, explanation of any interim analyses and stopping guidelines	not applicable
Randomisation:			17,18
Sequence generation	8a	Method used to generate the random allocation sequence	
	8b	Type of randomisation; details of any restriction (such as blocking and block size)	17,18
Allocation concealment mechanism	9	Mechanism used to implement the random allocation sequence (such as sequentially numbered containers), describing any steps taken to conceal the sequence until interventions were assigned	17,18
Implementation	10	Who generated the random allocation sequence, who enrolled participants, and who assigned participants to interventions	18
Blinding	11a	If done, who was blinded after assignment to interventions (for example, participants, care providers, those assessing	18

		outcomes) and how	
	11b	If relevant, description of the similarity of interventions	20
Statistical methods	12a	Statistical methods used to compare groups for primary and secondary outcomes	21,22
	12b	Methods for additional analyses, such as subgroup analyses and adjusted analyses	22,23
**Results**			
Participant flow (a diagram is strongly recommended)	13a	For each group, the numbers of participants who were randomly assigned, received intended treatment, and were analysed for the primary outcome	5,6
	13b	For each group, losses and exclusions after randomisation, together with reasons	5,6
Recruitment	14a	Dates defining the periods of recruitment and follow-up	16
	14b	Why the trial ended or was stopped	not applicable
Baseline data	15	A table showing baseline demographic and clinical characteristics for each group	6,29
Numbers analysed	16	For each group, number of participants (denominator) included in each analysis and whether the analysis was by original assigned groups	6
Outcomes and estimation	17a	For each primary and secondary outcome, results for each group, and the estimated effect size and its precision (such as 95% confidence interval)	6,7
	17b	For binary outcomes, presentation of both absolute and relative effect sizes is recommended	6,7
Ancillary analyses	18	Results of any other analyses performed, including subgroup analyses and adjusted analyses, distinguishing pre-specified from exploratory	8,9
Harms	19	All important harms or unintended effects in each group (for specific guidance see CONSORT for harms)	7,8
**Discussion**			
Limitations	20	Trial limitations, addressing sources of potential bias, imprecision, and, if relevant, multiplicity of analyses	13,14
Generalisability	21	Generalisability (external validity, applicability) of the trial findings	13
Interpretation	22	Interpretation consistent with results, balancing benefits and harms, and considering other relevant evidence	13
**Other information**			
Registration	23	Registration number and name of trial registry	15
Protocol	24	Where the full trial protocol can be accessed, if available	Supplementary information
Funding	25	Sources of funding and other support (such as supply of drugs), role of funders	27

Citation: Schulz KF, Altman DG, Moher D, for the CONSORT Group. CONSORT 2010 Statement: updated guidelines for reporting parallel group randomised trials. BMC Medicine. 2010;8:18.
© 2010 Schulz et al. This is an Open Access article distributed under the terms of the Creative Commons Attribution License (http://creativecommons.org/licenses/by/2.0), which permits unrestricted use, distribution, and reproduction in any medium, provided the original work is properly cited.

1. Original Protocol and Summary of Changes

2. Original Statistical Analysis Plan and Summary of Changes

Original Study Protocol

(January 8, 2020)

Feasibility Clinical Study of Embryo Optimization Using Free DNA Euploidy in Embryo Culture Media

(Multi-center, double-blind, randomized controlled trial)

Project leader: Qiao Jie

Sponsor:

Centre for Reproductive Medicine, Department of Obstetrics and Gynecology, Peking University Third Hospital, Beijing, China.

Partner:

Affiliated Jinling Hospital, Medicine School of Nanjing University, Nanjing, China

Reproductive Medical Center of Hebei Maternity Hospital, Shijiazhuang, China

Northwest women's and Children's Hospital, Xi'an, China

Department of Obstetrics and Gynecology, Chinese People's Liberation Army General Hospital, Beijing, China

Shengjing Hospital of China Medical University, Shenyang, China

Guangdong Provincial Key Laboratory of Reproductive Medicine, First Affiliated Hospital, Sun Yat-sen University, Guangzhou, China

Reproductive and Genetic Hospital of Citic-Xiangya, Changsha, China

Yikon Genomics Company, Ltd., Suzhou, China

ABSTRACT

Introduction

Morphological evaluation is widely used to select embryos for in vitro fertilization (IVF) cycles. However, it does not fully reflect the implantation potential and ploidy of the embryo. Preimplantation genetic testing for aneuploidies (PGT-A) can detect aneuploidy in embryos, but the biopsy procedure is invasive. Currently, a non-invasive PGT (ni-PGT) approach utilizing spent culture medium is being evaluated in studies. However, the clinical value of ni-PGT for evaluating embryo competence thus far has not been justified in randomized clinical trials. Hence, a multicentre randomized trial is needed to provide evidence to verify whether ni-PGT can be used as a new effective indicator for evaluating embryos.

Methods and analysis

Overall, 1148 couples aged 35~42 (female) receiving IVF-ICSI will be enrolled. Couples will be randomized according to a unique grouping number generated by random digital software into (1) ni-PGT and (2) morphology (conventional embryo morphology evaluation) groups at a 1:1 treatment ratio. This study aims to investigate whether ni-PGT can be used as a new effective indicator for evaluating embryos. The primary outcome is the ongoing pregnancy rate of the first transfer cycle within 3 months after oocyte retrieval.

Ethics and dissemination

Ethical permission was acquired from the Ethics Committee of Peking University Third Hospital Medical Science Research Ethics Committee. The results will be disseminated through publications in scientific journals and conference reports.

Trial registration number NCT04339166.

Strengths and limitations of this study

This will be the first multicentre randomized trial with a relatively large sample size to investigate the effectiveness of embryo selection by ni-PGT for the treatment of aged women in 8 centres across China.

This study will provide evidence on whether the ni-PGT results could be another criterion for embryo selection.

A cost-effectiveness analysis is not included in the study, which limits the comprehensiveness of the results.

Contents

1 Introduction 17

2 Study purpose 18

3 Study design 18

4 Study population 20

5 Trial procedure 21

6 Drug management 25

7 Trial evaluation 25

8 Adverse events 26

9 Statistical analysis 27

10 Data management 27

11 Inspection procedures 28

12 Informed consent, ethical review, and regulatory issues 29

13 Reporting and publication 30

14 Reference 30

1 Introduction

Morphological evaluation is widely used for embryo selection in IVF-ET (in vitro fertilization-embryo transfer) but is incapable of detecting chromosomal abnormalities[1-3]. Embryo chromosomal abnormalities occur due to errors during cell mitosis and meiosis [4 5]. The occurrence of chromosomal abnormalities is substantially higher in patients of advanced maternal age. The probability of chromosome aneuploidy in women between 26 and 34 years of age is approximately 20% to 31%. When the age is ≥35 years, the incidence of aneuploidy in oocytes and embryos gradually increases (34% to 75%)[6 7]. Studies have demonstrated that aneuploidies cause embryo implantation failure or embryonic development arrest[8]; therefore, the probability of successful natural and IVF pregnancy is significantly reduced in older women with increased miscarriage rates.

To improve IVF outcomes, embryos were evaluated using preimplantation genetic testing for aneuploidy (PGT-A) before implantation under specific indications, such as women of advanced age and patients with recurrent miscarriage or recurrent implantation failure. For PGT-A, embryos were biopsied and tested for chromosomal ploidy before transferring into the uterus. Clinical studies have shown that the clinical outcomes of IVF were indeed improved by PGT-A[9 10]. The problem, however, is that such a biopsy procedure is invasive and conveys unknown health risks in the long-term development of embryos[11-14]. Therefore, a less invasive or even non-invasive method that evaluates the ploidy status of embryos is highly preferred in the field of IVF. In recent years, multiple studies have been published using spent culture medium for the detection of chromosomal ploidy. Researchers have found that the spent medium of embryo cultures contains trace amounts of cell-free DNA, which may reflect the ploidy of the embryo. This approach utilizing spent culture medium samples, named niPGT-A, has been evaluated in studies[15-22]. However, the clinical value of niPGT-A as a new effective indicator to evaluate embryo competence has not been justified by randomized clinical trials thus far. The primary purpose of this project is to establish a method for evaluating embryo developmental potential in a large-scale randomized controlled clinical trial.

2 Study purpose

The primary purpose of this project is to establish a method for evaluating embryo developmental potential in a large-scale randomized controlled clinical trial.

3 Study design

3.1 Overall design

This is a multicentre randomized controlled clinical trial that is designed to compare the ongoing pregnancy rate of first embryo transfer in 1148 aged couples undergoing ICSI. Participants will be enrolled at 8 hospitals in China. This study was approved by the Ethics Committee of Peking University Third Hospital Medical Science Research Ethics Committee and the participating hospitals. Informed consent will be obtained from the enrolled couples before any study procedures are performed. This protocol was written following the Standard Protocol Items: Recommendations for Interventional Trials. The trial design is summarized in Figure 1.

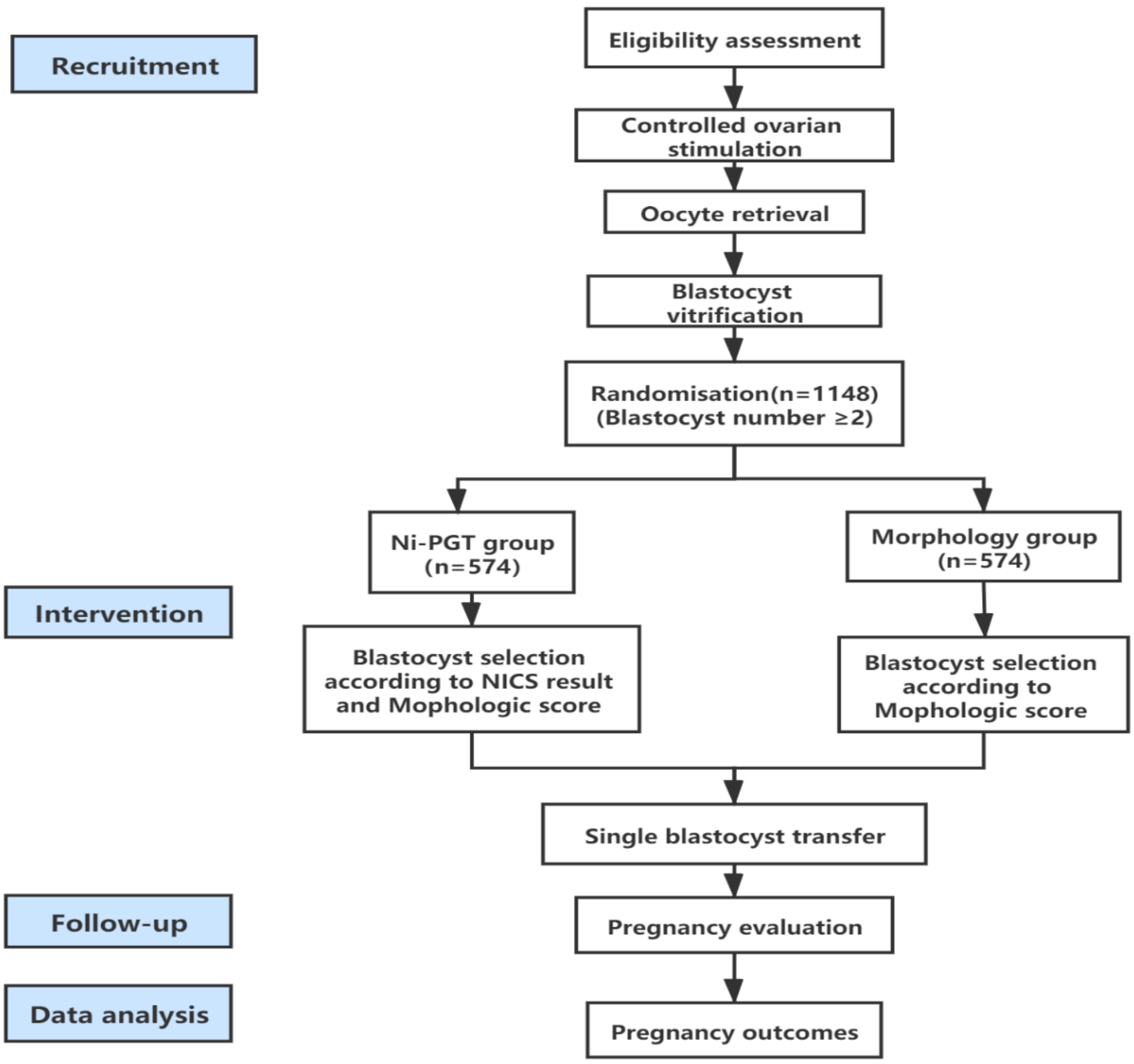

Figure 1 Flowchart of this randomised controlled trial.

3.2 Study centers

This study will recruit participants from 8 reproductive medical centres across China: Peking University Third Hospital, Affiliated Jinling Hospital, Medicine School of Nanjing University, Reproductive Medical Centre of Hebei Maternity Hospital, Northwest women's and Children's Hospital, Chinese PLA General Hospital, Shengjing Hospital of China Medical University, First Affiliated Hospital, Sun Yat-sen University, Reproductive and Genetic Hospital of Citic-Xiangya, Yikon Genomics and Xukang Medical Science & Technology (Suzhou) will provide ni-PGT testing using non-invasive chromosome screening (NICS).

3.3 Sample size calculation

The primary hypothesis of this study is that ni-PGT results will increase the ongoing pregnancy rate and decrease the miscarriage rate. According to previous work, in the 35-42 age group, the ongoing pregnancy rate of embryos selected according to "morphology" was approximately 38.8%, and it is expected that there will be a difference of 10% in the ongoing pregnancy rate between the two groups. To demonstrate this difference using a two-sided test with a 5% alpha error and 90% statistical power, at least 516 subjects will be included in each group, with a total of 1032 subjects (ni-PGT group: morphology group = 1:1). Considering a dropout of 10%, each group included 574 participants (a total of 1148 participants).

3.4 Randomization

Randomization and allocation of eligible patients to this study will be performed when the couples have ≥2 blastocysts. This procedure will be performed by administrative staff in the trial centre who are not involved in the treatment procedure using an online trial system using a computer-generated randomization list that allocates couples at a 1:1 ratio to the ni-PGT or morphology group, with a block size of four, stratified for the centre.

3.5 Blinding

This trial is designed and will be performed as a double-blind trial in which participants and clinicians/nurses who perform embryo transfer or follow-up, as well as the investigators and assessors, will be blinded to participant allocation until the primary outcome occurs. Only embryologists will know the allocation because they will rank the thawed blastocysts.

In this study, Yikon Genomics and Xukang Medical Science & Technology will also be blinded.

3.6 Time management

The primary outcome: ongoing pregnancy rate (12 weeks of gestation), the observation time point is 12 weeks after the first transplantation, within three months after oocyte retrieval.

In order to evaluate the effectiveness of this trial, the following secondary outcomes will also be evaluated: ① Clinical pregnancy rate; ② Miscarriage rate; ③ Live birth rate.

After the participants signed the informed consent form, it marked the beginning of the trial observation. When the participant is confirmed to thaw the embryo to transplant for the first time within three months after the oocyte retrieval, the follow-up after pregnancy to the delivery outcome marks the end of the trial observation; if the participant suspends the trial and withdraws from the trial, it also marks the end of the trial observation.

4 Study population

4.1 Inclusion criteria

1. Infertile couples receiving ICSI procedures for assisted reproduction cycle.
2. Female age: 35 - 42 years old with a BMI range of 18 – 30 kg/m2.
3. Women received either a gonadotrophin-releasing hormone agonist (GnRH-a) protocol or a gonadotrophin-releasing hormone antagonist (GnRH-ant) protocol as their controlled ovarian hyperstimulation (COH) treatment, and the number of oocytes was ≥6.
4. All embryos were from blastocyst cultures and were individually vitrified.
5. Single thawing blastocyst transfer was performed within 3 months after oocyte retrieval.
6. Only patients whose blastocysts are ≥2 and whose blastocyst morphology is greater than 4BC/4CB will be included.

4.2 Exclusion criteria

1. Couples with a contraindication for IVF or ICSI.
2. Those with preimplantation genetic testing cycles.
3. Women who have pathologies or malformations that affect pregnancy outcome, including genital malformations, untreated hydrosalpinx, untreated intrauterine infection, myoma of uterus greater than 4 cm, benign tumour of pelvic or abdominal cavity greater than 4 cm, endometrial thickness less than 8 mm, pituitary tumours, and malignant tumours.
4. Untreated hyperprolactinemia, thyroid disease, and adrenal disease.
5. Women with endometrial polyps that were not treated before the thawing transfer cycle.

4.3 Exit criteria

-Participants are unwilling to continue the trial process, violate the trial protocol, or withdraw consent.

-Participants have the right to withdraw their consent and quit at any stage of the trial. Any of their decisions will not affect the clinical treatment.

4.4 Benefits to participants

-Benefits of participants in the study group:

① The study results are expected to increase the clinical pregnancy rate, decrease the miscarriage rate, and reduce time to pregnancy;

② After the first FET, although the study of this trial is over, the participant can still choose the embryo according to the thawing order of the NICS sort level in the subsequent FET;

③ After the thawing embryo transfer for the first time, whether pregnant or not, the NICS level can be informed to provide a reference for future diagnosis and treatment. If the patient has a clinical miscarriage after the first transfer. Free testing of the chromosomal euploidy of the aborted tissue is provided.

-Benefits of participants in the control group:

① After the first FET, if the participant has not obtained a clinical pregnancy, the participant can be informed of the NICS level of the corresponding embryo, and the possible reasons can be analyzed to provide a reference for future diagnosis and treatment;

② After the first FET, if the participant has not obtained a clinical pregnancy and there are remaining blastocysts that have been detected by NICS, the embryos with high NICS grade can be transferred preferentially;
③ If the patient has a clinical miscarriage after the first transfer. Free testing of the chromosomal euploidy of the aborted tissue is provided.

-Benefits of participants who signed informed consent but were not included in any group.
① Participants without blastocyst formation: review the participant's diagnosis and treatment process, analyze the possible factors for the formation of blastocyst, and make recommendations for subsequent diagnosis and treatment.
② Participants who have formed a blastocyst: keep the spent culture media for NICS testing. After thawing and transferring the embryo, whether pregnant or not, the NICS level can be informed to provide a reference for future diagnosis and treatment. If the patient has a clinical miscarriage after first transfer. Free testing of the chromosomal euploidy of the aborted tissue is provided.

5 Trial procedure

The trial visit form of the subject is shown in Table 5-1

Table 5-1. Schedule of trial visit form

**Content**	Study period							
	Enrolment	Pre-allocation		Allocation	Post-allocation			
	Screening and baseline assessment	Oocyte retrieval	Embryo culture	Blastocysts vitrification and randomisation	Embryo transfer	Evaluation of pregnancy	Follow-up of pregnancy	
**Time point**	T0	T1	T2	T3	T4	T5	T6	T7
	During the ovarian stimulation cycle	The day of oocyte retrieval	During embryo culture	The day of blastocyst vitrification	The day of blastocyst transfer	28-30 days after embryo transfer	12 weeks of pregnancy	Within 2 weeks after delivery
**Enrolment**								
**Eligibility screen**	×	×	×					
**Informed consent**	×	×						
**Allocation**				×				
**Interventions**								
**NICS+Mophology**				×				
**Mophology**				×				
**Assessments**								
**Baseline data**	×	×						

**Laboratory tests**	×	×			×	×	×	×
**Fertilisation**			×					
**Embryo quality**			×		×			
**Pregnancy tests**						×		
**Pregnancy outcomes**							×	×
**Fetus information**							×	×
**Neonate information**							×	×
**Safety assessment**	×	×			×	×	×	×

5.1 Recruitment and selection

Infertile couples who present to the outpatient department will be screened by a dedicated research team. Eligible couples will then, prior to oocyte retrieval, be explained the trial details by a member of the research team. After this information, couples will be offered time for consideration to decide whether to participate in the trial. Couples who agree to participate will be asked to sign the consent form at their next scheduled visit. On the day of oocyte retrieval, those who signed the consent form will be screened again for exclusion criteria. Ineligible patients will be further excluded from the trial, continuing their conventional clinical procedures instead.

5.2 Controlled ovarian hyperstimulation

All couples will receive COH treatment, which will be performed by the standard routine according to each study centre. COH treatment includes either the GnRH-a protocol or the GnRH-ant protocol, and selection of the protocol will be done by physicians. In the GnRH-ant protocol, participants will be injected with gonadotropin (Gonal-F, Pouliquen or HMG) daily from cycle day 2 or 3. When at least one follicle has reached a diameter of 12 mm or on day 6 of ovarian stimulation, GnRH-ant (Cetrotide or Ganirelix) will be administered subcutaneously until the trigger day (including on the trigger day). For the super long GnRH-a protocol, GnRH-a (alarelin or trip-torelin) will be used in the previous menstrual cycle, and gonadotropin treatment starts after 28–35 days on GnRH agonist downregulation. For the long GnRH-a protocol, pituitary downregulation will be initiated 7–10 days before the menstrual cycle with GnRH-a (alarelin or triptorelin). After 10–14 days or on day 2 of the menstrual cycle, gonadotropin treatment will start. For the short GnRH-a protocol, participants will receive alarelin or triptorelin for pituitary downregulation on day 2 or 3 of the menstrual cycle. Gonadotropin will be used at the same out. For the above treatments, the menstrual cycle of patients includes a spontaneous menstrual cycle and an irregular menstrual cycle with the use of oral contraceptives or progestins. Before gonadotrophin treatment, baseline pelvic ultrasound, as well as basic serum hormones (such as FSH, luteinising hormone, progesterone (P) and β-human chorionic gonadotropin (β-hCG)), will be measured to confirm follicle status. The initial dosage of gonadotrophin (Gonal-F or Pouliquen or HMG) is 150–300 mg/day, and the subsequent dose will be adjusted according to the individual response. Gonadotrophin treatment will be continued until the trigger day. After two or more follicles reach a diameter ≥18 mm, 250 µg of hCG (Ovitrelle, 250 µg sc) will be injected once on the trigger day.

5.3 Oocyte retrieval and preparation

Oocyte retrieval is scheduled for 36 hours (±2) after hCG injection. Routine oocyte retrieval is performed under transvaginal ultrasound guidance using a 17–18 G oocyte aspiration needle with under intravenous sedation. The retrieved cumulus oocyte complexes (COCs) will be placed in culture medium covered by lightweight paraffin oil and incubated in a humidified 37°C, 5%-6% CO2 incubator immediately after oocyte retrieval.

5.4 Sperm preparation

After male abstinence for 2-7 days, a fresh sample of ejaculated semen will be collected by masturbation on the day of oocyte retrieval. The conventional semen parameters will be tested by professional semen analysis equipment, and according to the fourth or fifth edition of the World Health Organization (WHO) semen standards assess semen quality. After ensuring that the semen meets the standard, the semen will be liquefied at room temperature for 15 minutes. Then the semen will be processed by conventional density gradient centrifugation or upstream methods.

5.5 ICSI

All oocytes in this study will undergo ICSI, which has been previously described[23]. In short, as enzymatic removal will be performed during oocyte preparation, the denuded oocytes will be examined to assess their integrity and maturity. Only oocytes that have extruded the first polar body (metaphase-II oocytes) will be microinjected.

5.6 Assessment of fertilization and embryo quality

Assessment of fertilization will be performed approximately 16–18 hours (day 1) after fertilization. Normal fertilization will be assessed by the presence of two pronuclei and a second polar body. The zygotes will be cultured in cleavage medium to day 3, and cleavage embryo quality will be observed 67–69 hours (day 3) after fertilization. The embryos will be scored according to quality, number, size of the blastomeres, and the amount of anucleate fragmentation.

According to morphological criteria, the day 3 embryos can be divided into 4 levels:

Grade I (Grade 1): Cells are uniform in size, regular in shape, zona pellucida is intact; cytoplasm is uniform and clear, no granular phenomenon, no debris.

Grade II (Grade 2): The cell size is slightly uneven, and the shape is slightly irregular; the cytoplasm may be granular; the fragments are less than 10%.

Grade III (Grade 3): The cell size is obviously uneven, and there may be obvious irregular shapes; the cytoplasm may be granular; the fragments are between 10%-30%.

Grade IV (Grade 4): The cell size is severely uneven; the cytoplasm may be severely granular; the fragment is between 30%-50%.

Uneven blastomere size: indicated by a minus sign (-).

5.7 Embryo blastocyst culture and specimen collection

Before ICSI, the oocytes will be denuded. If cumulus cells are still attached to the oocyte, the remnant can be removed later on day 3 before blastocyst culture. On day 4, the embryos will be individually transferred into a new blastocyst culture dish. The volume of each droplet of culture medium cannot exceed 25 μL. When embryos develop to the blastocyst freezing standard, the blastocyst will be vitrified, and the culture medium from each corresponding blastocyst will be collected into an RNase/DNase-free PCR tube.

5.8 Blastocyst vitrification and warming, endometrium preparation, blastocyst transfer, and luteal support

The expanded blastocysts that collapse after artificial shrinkage will be vitrified and warmed as previously reported[24]. If the blastocyst needs to shrink before freezing, it is transferred into another droplet instead of the culture dish.

The vitrification and warming procedure will be performed according to the standard of each centre. In this study, the first thawing cycle will include a single blastocyst transfer in both the ni-PGT and morphological groups. The endometrium preparation and luteal support methods will be performed according to the standard of each centre.

5.9 Random point setting:

Participants with ≥ 2 blastocysts, morphological ratings (4BC/4CB or above) will enter the randomized controlled study. They will be randomly assigned to the study group or the control group.

Process of the study group:

When cryopreserving blastocysts, code the serial numbers n1, n2, n3...; Before sampling, don't hatch the embryos.

Thawing order: ① NICS rating: A>B>C ② In the case of the same level of NICS, the order of embryo morphology see "5.10".

Process of the control group:

When cryopreserving blastocysts, code the serial numbers n1, n2, n3...; Before sampling, don't hatch the embryos.

Thawing order: when freezing, confirm the number of the first transferred embryo according to the embryo morphology; the embryo morphology sequence see "5.10".

5.10 Embryo morphology rank

Blastocyst formation time: D5>D6>D7

5AA>5AB>5BA>4AA>4AB>4BA>6AA>6AB>6BA>5BB>4BB>6BB>5AC>5BC>4AC>4BC>6AC>6BC>5CA>5CB>4CA>4CB>6CA>6CB

5.11 Pregnancy assessment

All participants will undergo measurement of the hCG in urine and blood 12 days after blastocyst transfer, in which positive results indicate biochemical pregnancy. Twenty-eight days after transfer, if the gestational sac is observed by ultrasonography, participants will be diagnosed with clinical pregnancy. Ongoing pregnancy is defined as a sac with a foetal heartbeat and occurs after 12 weeks of pregnancy.

5.12 Follow-up

We will track the delivery status of all enrolled patients. We will collect the following information within 2 weeks after delivery: prenatal testing information (pregnancy complications and foetal information), delivery information (gestational age, mode of delivery and birth complications) and new-born information (foetal sex, birth weight and birth defects).

6 Drug management

Relevant medications in the trial will be administered according to the routine clinical administration procedures of each center, and individual s will not be arranged to be administered.

7 Trial evaluation

7.1 Demographic information

After the couples sign the informed consent form, the researcher should obtain the couple's demographic information during the screening visit period. The demographic information, medical records, and vital signs information of the couples were obtained before the oocyte retrieval.

Demographic information includes: date of birth, age, height, weight, education level, occupation, etc.

The case data should focus on the history of past diseases, the history of pregnancy and childbirth, history of drug allergy, history of surgery, history of IVF and/or ICSI assisted pregnancy, history of PGS and medication, history of personal adverse habits, etc.

Vital signs include: body temperature, pulse, breathing, blood pressure, etc.

7.2 Laboratory project

Perform necessary laboratory inspections on participants during the screening period, during the trial, and after the end of the trial, to ensure that participants meet the inclusion criteria of this trial. It is helpful to simultaneously observe and judge the effectiveness and safety of patients participating in this trial. The laboratory examination items of the entire clinical trial include six serum sex hormones, basic antral follicle count, semen routine examination, and pregnancy test (see Table 7-1 for details).

Table 7-1 Check item content list

Number	Project name	Specific testing items	Specimen source	Requirement
1	Six serum sex hormones	Estradiol (E2)	Serum	Days 2-5 of the menstrual cycle
		Luteinising hormone (LH)		
		Follicle-stimulating hormone （FSH）		
		Prolactin (PRL)		
		progesterone (P)		
		Testosterone (T)		
2	Basic antral follicle count	Antral follicle counting (AFC)	Vagina ultrasound	Non-ovulatory period
3	Semen routine	Semen characteristics	Semen(masturbation)	Abstinence

	examination	Total sperm count		for 2-7 days
		Sperm density		
		Sperm motility rate(a+b+c)		
		Sperm motility (a or a+b)		

Other laboratory items must be checked during the ART ovarian stimulation cycle, oocyte retrieval and fresh embryo transfer cycle. The laboratory items, including blood routine, urine routine, blood coagulation function, eight immune items, thyroid function, liver and kidney function, cervical cytology smear, leucorrhea routines, etc., are not considered as observation items in this trial. However, the clinicians should conduct these examinations according to clinical routines after the trial starts.

7.3 Trial outcome:

The primary outcome: ongoing pregnancy rate (12 weeks of gestation), the observation time point is 12 weeks after the first transplantation within three months after oocyte retrieval.

In order to evaluate the effectiveness of this trial, the following secondary outcomes will also be evaluated: ① Clinical pregnancy rate; ② Miscarriage rate; ③ Live birth rate.

7.4 Study Endpoint:

Fourteen months after the oocyte retrieval. The live birth of the first embryo transfer within three months after the oocyte retrieval was observed for the first time.

8 Adverse events

8.1 Definition of adverse events

Adverse events: adverse events refer to any adverse medical condition or deterioration of the existing medical condition that occurs during ovarian stimulation and thawing transplantation, regardless of whether it is causally related to the treatment. The adverse medical condition can be symptoms (such as nausea, chest pain), physical signs (such as tachycardia, hepatomegaly), or abnormal test results (such as laboratory tests, electrocardiogram). In clinical research, adverse events refer to adverse medical conditions that occur at any time (even including the period that participants haven't received treatment but have signed the informed consent form).

Serious adverse events: refer to adverse events that meet one or more of the following criteria that occur at any stage of the study (such as the screening period, the baseline period, and the follow-up period): death, immediate life-threatening, hospitalization or extension of current hospitalization time, permanent or obvious disability/dysfunction, or seriously affect normal life ability, congenital malformations or other events of birth defects.

8.2 Collection of adverse events

The investigator must monitor the condition of the participants during the entire trial period from the time of obtaining informed consent to the end of the trial visit. Collected from the following

aspects: the participants' answers to questions about their health, the symptoms spontaneously reported by the participants, the findings in laboratory tests and examinations that were evaluated as clinically significant changes or abnormalities by the investigator, other information about the participants' health that the investigator knows.

8.3 Records of adverse events

The investigator must record all adverse events in the adverse event record provided in the case report form of each subject, including the following information: adverse event name, start date and time, intensity, measures taken, date and time of outcome, outcome, severity, and relevance to trial intervention.

9 Statistical analysis

The result will be analyzed according to the intention-to-treat (ITT) principle. Pre-protocol (PP) analysis will also be conducted as a sensitivity analysis. Baseline characteristics will be described by descriptive analysis. For continuous variables, the normality distribution will be estimated by using frequency histograms and the Shapiro test initially. If the parameters are normally distributed, they will be presented as means with standard deviations (SDs). If the parameters are non-normally distributed, their medians and inter-quantile ranges (IQRs) will be reported. For categorical variables, we will present the proportion between each group. In addition, the recruitment numbers, those participants lost to follow-up, protocol violations, and other relevant data will also be reported. Comparison between groups will be performed using the independent sample t test, Mann-Whitney U test, Pearson chi-square test, or Fisher's exact test as appropriate. The primary outcome, ongoing pregnancy, will be compared using Pearson's chi-square test or Fisher's exact test as appropriate. Categorical secondary outcomes will be compared between the two groups using a similar approach to the primary outcome. Student's t test or Wilcoxon test will be used as appropriate for continuous secondary outcomes, such as birthweight, etc. The absolute rate differences (ARD) and their 95% confidence interval (CI) between the two groups will be calculated. And the 95% CI of the ARDs will be used to evaluate if the experimental group (ni-PGT) is noninferior to the control group (Morphological).

The missing data will be treated as missing at random and will be imputed using the last observation carried forward (LOCF) method. For the missing values, a sensitivity analysis will be done under the hypothesis of the worst and the best outcomes for each missing individual. Therefore, all secondary outcomes will be considered exploratory.

All statistical analyses will be done using the statistical package SPSS, version 25.0 (SPSS Inc., Chicago, IL). Statistical significance is defined as $p < 0.05$ with two-sided testing, except for the non-inferiority test for the primary outcome.

10 Data management

10.1 Randomization

After completing the blastocyst culture and forming ≥2 blastocysts, patients who meet all the inclusion criteria without any exclusion criteria will be randomly assigned.

The embryo laboratory personnel are responsible for randomization and numbering. During clinical implementation, each center assigns screening numbers according to the clinical order of participants who meet the inclusion criteria, and uploads them to the electronic randomization platform. Laboratory personnel perform multi-center randomization through the platform and determine the random number. Researchers in each center conduct clinical treatment observations according to the assisted reproduction program corresponding to the random number. The study number (random number) will appear as the participant's identity information throughout the clinical observation process, thereby protecting the participant's identity when reporting adverse events and/or other trial-related data.

10.2 Data management

The data entry of this study will be carried out simultaneously in the clinical data collection process. Questions found in the data entry process will be notified in writing to the researcher in the form of a questionnaire, which will be reviewed by the researcher, and the response to the question will be fed back to the data entry/review staff in writing as soon as possible. The written feedback record will be saved with the case report form as the basis for data modification. For all participants who have passed the screening, entered the trial, and signed the informed consent form, all items in the case report form must be recorded carefully and in detail, and no omissions should be made. The completed case report form shall not be provided to a third party in any form without the written consent of the sponsor.

10.3 Data Lock

After reviewing and confirming that the established database is correct, the main research unit, the cooperative research unit, and the statistician will jointly lock the database. The locked data file is not allowed to be changed. The statistical plan must be completed and approved by the main research unit and the cooperative research unit before the database is locked.

11 Inspection procedures

11.1 Periodic audit

The supervision of this study consists of two parts: self-examination by each research center and CRA supervision by a third-party clinical supervision team.

Each center will set up special personnel as data management personnel to timely monitor the data of the research center to ensure compliance with the trial protocol, standard operating procedures and relevant laws and regulations, to ensure the retention of test-related source records, and to ensure the completeness, accuracy and verifiability of the comparison between the electronic medical record form and the source data.

The relevant personnel of the Clinical Epidemiology Center of Peking University Third Hospital will conduct data monitoring work on the data of each research center as a third party, and will regularly contact and visit researchers according to the monitoring plan to ensure compliance with the trial protocol, standard operating procedures, and relevant laws and regulations. It is required to

ensure the retention of trial-related source records and the completeness, accuracy and verifiability of the comparison between the electronic medical record form and the source data. The researcher should allow the inspector to directly access all source data, including electronic medical records and/or documents, to facilitate data verification. Researchers and monitors work together to ensure that any discernible differences are resolved. The investigator is expected to be able to meet with the monitor during these visits.

11.2 Audit and inspection

The main purpose of the audit or inspection is to assess whether the trial is complied with the protocol, the relevant principles and regulations. The research unit should be able to provide all trial-related source data and records to quality assurance inspectors appointed by third-party agencies, or domestic/foreign regulatory inspectors, or representatives of ethics committees who can conduct audit/inspections of trials.

Researchers must inform participants that representatives appointed by third-party agencies, regulatory agencies and ethics committees may wish to view their medical records. This content needs to be written in the informed consent form. During the inspection/inspection, the inspector can copy the relevant part of the medical record.

11.3 Confidentiality of participants' data

The research unit will ensure the confidentiality of the participants' data. In electronic medical records or any other documents, participants will not be identified by their names, but by an identification system consisting of designated numbers in the trial. The participants' identification code and signed informed consent form will be kept strictly confidential by the investigator.

12 Informed consent, ethical review, and regulatory issues

12.1 Informed Consent

Ensure that participants fully understand the necessary information related to this trial, including the nature, purpose, methods, procedures, time limit, inspection items and frequency, total number of specimens, etc., and fully understand the risks and benefits of participating in this trial.
Answer any questions the participants met during the trial, and promptly convey to the participants any new information that may affect whether they are willing to continue participating in the trial or not.

Before the participants enter the trial, the researchers use the informed consent form to explain the risks and benefits of participating in the trial in plain, simple, and easy-to-understand language, and record that the participants are satisfied with these risks and benefits. They understand and are willing to participate in the experiment. The participants should be informed that they can voluntarily choose to participate in the trial and withdraw from the research in the middle of the trial. The personal data participating in the trial is confidential, and the records related to the participants' identification should be kept confidential. If the trial results are published publicly, the identities of the participants will also be kept confidential. However, the ethics committee, the drug and food supervision and management department, and the research sponsor can consult the participants' data in the trial as required by the work.

If necessary, the investigator can suspend the participants to continue the trial without the participants' consent. The investigator is responsible for ensuring that each participant or legal representative agrees to the trial, including obtaining a signed and dated informed consent form before starting the steps of the research protocol and taking the experimental drugs.

12.2 The ethical requirements of the trial and the approval of the ethics committee

This trial will be conducted in accordance with all current regulations, including the "Declaration of Helsinki" in October 1996. The main research unit is responsible for ensuring that this trial protocol, the informed consent form used by the research center, and any other materials that inform the participants (such as advertisements or other related materials) are reviewed and approved by the local ethics committee. The ethics committee must be established in accordance with the requirements of all current registration regulations. Before the start of this trial, each cooperative research unit must obtain a copy of the approval of the ethics committee, the approved informed consent, and all other information approved by the ethics committee that should be notified to the participants.

13 Reporting and publication

13.1 Clinical trial report

The data and information collected during this trial will be reported in the clinical trial report prepared by Peking University Third Hospital. The report will be submitted to each center for comments and approval.

13.2 Data storage

In order to ensure the evaluation and supervision of clinical trials by relevant institutions and units, the research unit should agree to retain all trial-related data, including the original medical records of the participants, the original signed informed consent form, CRF, other detailed records, etc. The preservation time is 5 years after the end of the clinical trial.

13.3 Paper publication

As a result of the research, the relevant information obtained in this trial is considered confidential. After each research unit has completed the analysis and the statistical analyst has reviewed the data analysis, the research results can be published and demonstrated under the authorization and consent of the main research unit. However, the confidential or proprietary information cannot be disclosed.

14 Reference

1. Alfarawati S, Fragouli E, Colls P, et al. The relationship between blastocyst morphology, chromosomal abnormality, and embryo gender. Fertil Steril 2011;95(2):520-4.

2. Fragouli E, Alfarawati S, Spath K, et al. Morphological and cytogenetic assessment of cleavage

and blastocyst stage embryos. Mol Hum Reprod 2014;20(2):117-26.
3. Capalbo A, Rienzi L, Cimadomo D, et al. Correlation between standard blastocyst morphology, euploidy and implantation: an observational study in two centers involving 956 screened blastocysts. Hum Reprod 2014;29(6):1173-81.
4. McCoy RC, Demko ZP, Ryan A, et al. Evidence of Selection against Complex Mitotic-Origin Aneuploidy during Preimplantation Development. PLoS Genet 2015;11(10):e1005601.
5. Webster A, Schuh M. Mechanisms of Aneuploidy in Human Eggs. Trends Cell Biol 2017;27(1):55-68.
6. Yan J, Wu K, Tang R, et al. Effect of maternal age on the outcomes of in vitro fertilization and embryo transfer (IVF-ET). Sci China Life Sci 2012;55(8):694-8.
7. Franasiak JM, Forman EJ, Hong KH, et al. The nature of aneuploidy with increasing age of the female partner: a review of 15,169 consecutive trophectoderm biopsies evaluated with comprehensive chromosomal screening. Fertil Steril 2014;101(3):656-63 e1.
8. Scott RT, Jr., Ferry K, Su J, et al. Comprehensive chromosome screening is highly predictive of the reproductive potential of human embryos: a prospective, blinded, nonselection study. Fertil Steril 2012;97(4):870-5.
9. Chen M, Wei S, Hu J, et al. Can Comprehensive Chromosome Screening Technology Improve IVF/ICSI Outcomes? A Meta-Analysis. PLoS One 2015;10(10):e0140779.
10. Rubio C, Bellver J, Rodrigo L, et al. In vitro fertilization with preimplantation genetic diagnosis for aneuploidies in advanced maternal age: a randomized, controlled study. Fertil Steril 2017;107(5):1122-29.
11. Zhang S, Luo K, Cheng D, et al. Number of biopsied trophectoderm cells is likely to affect the implantation potential of blastocysts with poor trophectoderm quality. Fertil Steril 2016;105(5):1222-27 e4.
12. Guzman L, Nunez D, Lopez R, et al. The number of biopsied trophectoderm cells may affect pregnancy outcomes. J Assist Reprod Genet 2019;36(1):145-51.
13. Middelburg KJ, Heineman MJ, Haadsma ML, et al. Neurological condition of infants born after in vitro fertilization with preimplantation genetic screening. Pediatr Res 2010;67(4):430-4.
14. Middelburg KJ, van der Heide M, Houtzager B, et al. Mental, psychomotor, neurologic, and behavioral outcomes of 2-year-old children born after preimplantation genetic screening: follow-up of a randomized controlled trial. Fertil Steril 2011;96(1):165-9.
15. Stigliani S, Anserini P, Venturini PL, et al. Mitochondrial DNA content in embryo culture medium is significantly associated with human embryo fragmentation. Hum Reprod 2013;28(10):2652-60.
16. Rubio C, Navarro-Sanchez L, Garcia-Pascual CM, et al. Multicenter prospective study of concordance between embryonic cell-free DNA and trophectoderm biopsies from 1301 human blastocysts. Am J Obstet Gynecol 2020;223(5):751 e1-51 e13.
17. Rubio C, Rienzi L, Navarro-Sanchez L, et al. Embryonic cell-free DNA versus trophectoderm biopsy for aneuploidy testing: concordance rate and clinical implications. Fertil Steril 2019;112(3):510-19.
18. Yeung QSY, Zhang YX, Chung JPW, et al. A prospective study of non-invasive preimplantation genetic testing for aneuploidies (NiPGT-A) using next-generation sequencing (NGS) on spent culture media (SCM). J Assist Reprod Genet 2019;36(8):1609-21.
19. Kuznyetsov V, Madjunkova S, Antes R, et al. Evaluation of a novel non-invasive

preimplantation genetic screening approach. PLoS One 2018;13(5):e0197262.
20. Huang L, Bogale B, Tang Y, et al. Noninvasive preimplantation genetic testing for aneuploidy in spent medium may be more reliable than trophectoderm biopsy. Proc Natl Acad Sci U S A 2019;116(28):14105-12.
21. Jiao J, Shi B, Sagnelli M, et al. Minimally invasive preimplantation genetic testing using blastocyst culture medium. Hum Reprod 2019;34(7):1369-79.
22. Leaver M, Wells D. Non-invasive preimplantation genetic testing (niPGT): the next revolution in reproductive genetics? Hum Reprod Update 2020;26(1):16-42.
23. Oehninger S, Veeck L, Lanzendorf S, et al. Intracytoplasmic sperm injection: achievement of high pregnancy rates in couples with severe male factor infertility is dependent primarily upon female and not male factors. Fertil Steril 1995;64(5):977-81.
24. Chen Y, Zheng X, Yan J, et al. Neonatal outcomes after the transfer of vitrified blastocysts: closed versus open vitrification system. Reprod Biol Endocrinol 2013;11:107.

Summary of changes to the study protocol

Versions 1.0-3.0

Protocol version 1.0 (January 8, 2020)

Protocol version 2.0 (November 6, 2020)

Changes in Version 2 included:

1. **Update to research sites:**

   Added: "Peking University Shenzhen Hospital";
   "The Second Hospital of Hebei Medical University";
   "West China Second Hospital of Sichuan University";
   "The Second Affiliated Hospital of Wenzhou Medical University";
   "The Third Affiliated Hospital of Zhengzhou University"

2. **Study Endpoint:**

   The time for the first embryo transfer was changed from three months to six months after oocyte retrieval. This change aims to reduce the dropout rate of patients who are unable to return to the clinic on time due to COVID-19 pandemic.

The Version 2.0 has been published previously.

Huang J, Li R, Zeng L, Hu L, Shi J, Cai L, Yao B, Wang XX, Xu Y, Yao Y, Wang Y, Zhao J, Guan Y, Qian W, Hao G, Lu S, Liu P, Qiao J. Embryo selection through non-invasive preimplantation genetic testing with cell-free DNA in spent culture media: a protocol for a multicentre, double-blind, randomised controlled trial. BMJ Open. 2022 Jul 27;12(7): e057254.

Protocol version 3.0(June 2, 2023)

In this updated version, we modified the study endpoint:

1. **There will no longer be a time limit for the first embryo transfer.** The previous protocol required the completion of the first embryo transfer within six months of oocyte retrieval.
2. **The overall study endpoint is set at 17 months post-oocyte retrieval of the last randomized subject.** The last enrolled subject was allowed to complete the first thawed embryo transfer within six months of oocyte retrieval.

Because of the COVID-19, many participants were unable to return to the hospital for blastocyst transfer. This change aims to reduce the dropout rate of patients who are unable to return to the clinic on time.

Embryo Selection by Non-invasive Preimplantation Genetic Testing in Advance Aged Women: A Randomized, Clinical Trial

Statistical Analysis Plan

(Version 1.0; 06.21, 2021)

Approved by

Prof. Jie Qiao (Project Leader)

Signed: ____________________

Date: _____________________

Dr. Lin Zeng (Statistician)

Signed: ____________________

Date: _____________________

Contents

1. Administrative information 36
1.1 Study identifiers 36
1.2 Contributors to the statistical analysis plan 36
2. Introduction 37
2.1 Study synopsis 37
2.2 Study population 37
2.2.1 Inclusion criteria 37
2.2.2 Exclusion criteria 37
2.3 Study interventions 38
2.3.1 Randomization and masking 38
2.3.2 Study treatment 38
2.4 Outcomes 38
2.4.1 Primary outcome 38
2.4.2 Secondary outcomes 38
2.5 Sample size 39
3. Statistical analysis 39
3.1 Baseline characteristics 39
3.2 Primary outcome 40
3.3 Secondary outcomes 40
3.4 Missing data imputation 40
3.5 Sensitivity analyses 40
3.6 Adverse events analysis 40
4. Dummy tables and figures 41
Figure 1: Flowchart of the clinical trial 41
Table 1. Baseline characteristics 41
Table 2. Primary and secondary outcomes after the first embryo transfer cycle (intention-to-treat) 42
Table 3. Maternal and fetal/neonatal complications after the first embryo transfer cycle (intention-to-treat) 43
5. References 43

Members of Data and Safety Monitoring Board (DSMB)

Name	Specialty	Affiliation	Role in DSMB
Prof. TC Li	Reproductive Medicine	Reproductive Medicine and Surgery Unit, Prince of Wales Hospital, Chinese University of Hong Kong	Chair of DSMB
Prof. Hongtian Li	Epidemiology	Institute of Reproductive and Child Health, Peking University Health Science Centre	Voting member
Prof. Yunjun Zhang	Statistics	Department of Biostatistics, Peking University School of Public Health	Voting member

1. Administrative information

1.1 Study identifiers

- **Study title: Embryo Selection through Non-invasive Preimplantation Genetic Testing in Women of Advanced Age: A Multicentre, Double-blind, Randomized Clinical Trial**
- **Protocol number: Version 2.0, Date: June 05, 2024**
- **ClinicalTrials.gov register number: NCT04339166**

1.2 Contributors to the statistical analysis plan

Name	Affiliation	SAP contribution
Jie Qiao	Center for Reproductive Medicine, Peking University Third Hospital	Led, reviewed and revised every version
Ping Liu	Center for Reproductive Medicine, Peking University Third Hospital	Co-led, reviewed and revised every version
Lin Zeng	Clinical Epidemiology Research Center, Peking University Third Hospital	Co-led, reviewed and revised every version
Rong Li	Center for Reproductive Medicine, Peking University Third Hospital	Reviewed, and revised every version
Jin Huang	Center for Reproductive Medicine, Peking University Third Hospital	Prepared initial draft and revisions

2. Introduction

2.1 Study synopsis

This ESNI-PGT study is an investigator-initiated, multi-center, parallel, double-blind, randomized controlled clinical trial that compares outcomes after embryo transfers selected by non-invasive preimplantation genetic testing (niPGT) with those chosen by conventional morphological evaluation. The primary outcome is ongoing pregnancy after the first embryo transfer.

2.2 Study population

This study will be conducted in thirteen reproductive medical centers across China: Peking University Third Hospital, Reproductive and Genetic Hospital of Citic-Xiangya, Reproductive Medical Center of Hebei Maternity Hospital, Affiliated Jinling Hospital, Medicine School of Nanjing University, Northwest Women's and Children's Hospital, West China Second University Hospital, The Third Affiliated Hospital of Zhengzhou University, First Affiliated Hospital, Sun Yat-sen University, Peking University Shenzhen Hospital, Shengjing Hospital of China Medical University, The Second Affiliated Hospital of Wenzhou Medical University, The Second Hospital of Hebei Medical University, Chinese People's Liberation Army General Hospital.

2.2.1 Inclusion criteria

- Infertile couples receiving ICSI procedures for assisted reproduction cycle.
- Female age: 35 - 42 with a BMI range of 18 – 30 kg/m^2.
- Women received either a gonadotrophin-releasing hormone agonist (GnRH-a) protocol or a gonadotrophin-releasing hormone antagonist (GnRH-ant) protocol for their controlled ovarian hyperstimulation (COH) treatment, and the number of oocytes was ≥6.
- All embryos were derived from blastocyst cultures and individually vitrified.
- Only patients with at least two blastocysts and a blastocyst morphology greater than 4BC/4CB will be included.
- Informed consent was obtained.

2.2.2 Exclusion criteria

- Couples with a contraindication for IVF or ICSI.
- Those undergoing preimplantation genetic testing cycles.
- Women with pathologies or malformations that affect pregnancy outcome, including genital malformations, untreated hydrosalpinx, untreated intrauterine infections, myoma of uterus greater than 4 cm, benign tumour of pelvic or abdominal cavity greater than 4

cm, endometrial thickness less than 8 mm, pituitary tumours, and malignant tumours.

- Untreated hyperprolactinemia, thyroid disease, or adrenal disease.
- Women with endometrial polyps before the thawing transfer cycle.

2.3 Study interventions

2.3.1 Randomization and masking

Eligible patients with at least two blastocysts will be randomized 1:1 ratio to either the ni-PGT or the morphology group. This procedure will be performed by administrative staff not involved in the treatment procedure, using an online trial system with a computer-generated randomization list. This trial is designed as a double-blind trial, where participants, clinicians/nurses performing embryo transfers or follow-ups, the investigators, and assessors will be blinded to participant allocation until the primary outcome occurs. Only embryologists will know the allocation because they will rank the thawing blastocysts. Additionally, Yikon Genomics and Xukang Medical Science & Technology will also be blinded in this study.

2.3.2 Study treatment

The intervention is different embryo selection methods. In the niPGT group, the spent culture media will be collected and analyzed using non-invasive chromosome screening (NICS)[1]. Blastocysts will be classified into three grades (A to C) based on the NICS results, and a single blastocyst will be thawed and transferred in the preference order of A>B>C. In the morphological group, the embryo with the highest morphological score will be selected for transfer. Embryo morphology rank is 5AA>5AB>5BA>4AA>4AB>4BA>6AA>6AB>6BA>5BB>4BB>6BB> 5AC>5BC>4AC>4BC>6AC>6BC>5CA>5CB>4CA>4CB>6CA>6CB, which is the consensus of the 13 centers participating in this trial.

2.4 Outcomes

2.4.1 Primary outcome

Ongoing pregnancy after the first embryo transfer is defined as a viable pregnancy at 12 weeks gestation. The ongoing pregnancy rate is defined as the number of women with an ongoing pregnancy divided by the number of women randomized to each group.

2.4.2 Secondary outcomes

- Clinical pregnancy: defined as one or more observed gestational sacs or definitive clinical

signs of pregnancy under ultrasonography at 7 weeks of gestation. The clinical pregnancy rate is defined as the number of women with clinical pregnancies after the first transfer /number of women randomized to the specific group.

- Miscarriage: defined as a pregnancy that resulted in spontaneous abortion or therapeutic abortion that occurred throughout pregnancy before 28 weeks. First trimester miscarriage (early miscarriage) is defined as the spontaneous loss of an intrauterine pregnancy before 12 completed weeks of gestational age. The miscarriage rate is defined as the number of pregnancy losses /number of clinical pregnancies after the first transfer.

- Live birth: defined as a delivery of one or more living infants[2]. The live birth rate is defined as the number of women with live births after the first transfer /number of women randomized to the specific group.

2.5 Sample size

The primary hypothesis of this study posits that ni-PGT will increase the ongoing pregnancy rate. Our previous research indicates that in the 35-42 age group, the ongoing pregnancy rate for embryos selected based on morphology was approximately 38.8%, and we anticipate a 10% difference in the ongoing pregnancy rate between the two groups. To demonstrate this difference using a two-sided test with a 5% type 1 error rate and 90% statistical power, at least 516 subjects will be included in each group, with a total of 1032 subjects (ni-PGT group: morphology group = 1:1). Considering a dropout of 10%, each group will be included 574 participants, resulting in a total of 1148 participants.

3. Statistical analysis

All statistical analyses will be conducted using the statistical package SPSS, version 25.0 (SPSS Inc., Chicago, IL). Statistical significance is defined as $p < 0.05$ with two-sided testing.

3.1 Baseline characteristics

The baseline characteristics include female age (years), male age (years), female body-mass index (kg/m^2), duration of infertility (years), number of previous IVF/ICSI cycles, primary infertility, previous live birth(Yes or No), indication for IVF, endometrial thickness(mm), basic follicle-stimulating hormone (IU/L), basic luteinizing hormone (IU/L), basic estradiol (pmol/L), Basic progesterone (nmol/L), sperm volume (mL), COH treatment, number of days of ovarian stimulation, the total dose of follicle-stimulating hormone; levels of luteinizing hormone (IU/L), estradiol (pg/mL), progesterone (nmol/L), and endometrial thickness (mm) on hCG trigger day; the number of oocytes retrieved, number of good-quality embryos on day 3, number of blastocysts and number of patients undergoing embryo transfer. Continuous variables in each group will be

reported as means (±SD) or medians (with interquartile ranges). Categorical variables in each group will be reported as counts (with percentages).

3.2 Primary outcome

The intention-to-treat analysis will be the primary analysis for all primary and secondary outcomes. The ongoing pregnancy rate after the first embryo transfer in each group will be described using counts (with percentages). The difference between the two groups will be tested by Person chi-square analyses. The absolute difference and 95% CI, as well as relative risk (RR) and 95% CI will also be calculated.

3.3 Secondary outcomes

All secondary outcomes in each group will be described using counts (with percentages). The difference between the two groups will be tested by Person chi-square analyses. The absolute difference and 95% CI, as well as the relative risk and 95% CI will also be calculated. Categorical secondary outcomes will be compared between groups using aRRs and associated 95% CI.

3.4 Missing data imputation

The missing data will be considered as missing at random. The last-observation-carried-forward method will impute missing data. Missing data for the primary outcome (ongoing pregnancy after the first embryo transfer) will be imputed as "negative" irrespective of the reason why data are not recorded (e.g., lost to follow-up, no embryo transfer). Missing data for the secondary outcomes (clinical pregnancy) will be imputed based on the results observed at a later pregnancy assessment. Missing data for the secondary outcome of live birth will be imputed as "negative". For adverse events (AEs) and serious adverse events (SAEs), missing values will be treated as "negative" because all AEs and SAEs in this trial are self-reported by each site.

3.5 Sensitivity analyses

A per-protocol analysis will be conducted for the primary outcome and all secondary outcomes, excluding participants who did not adhere to the protocol. Besides, a sensitivity analysis will test best-case (all interventions considered positive and control negative) and worst-case hypotheses (all interventions considered negative and control positive) of the distribution of the missing values.

3.6 Adverse events analyses

The definition for adverse events (AEs) or serious adverse events (SAEs) is defined in the

protocol. All AEs and SAEs in this trial are self-reported by each site. The proportion and percentage of patients experiencing any AE or SAE will be presented by group.

4. Dummy tables and figures

Figure 1: Flowchart of the clinical trial

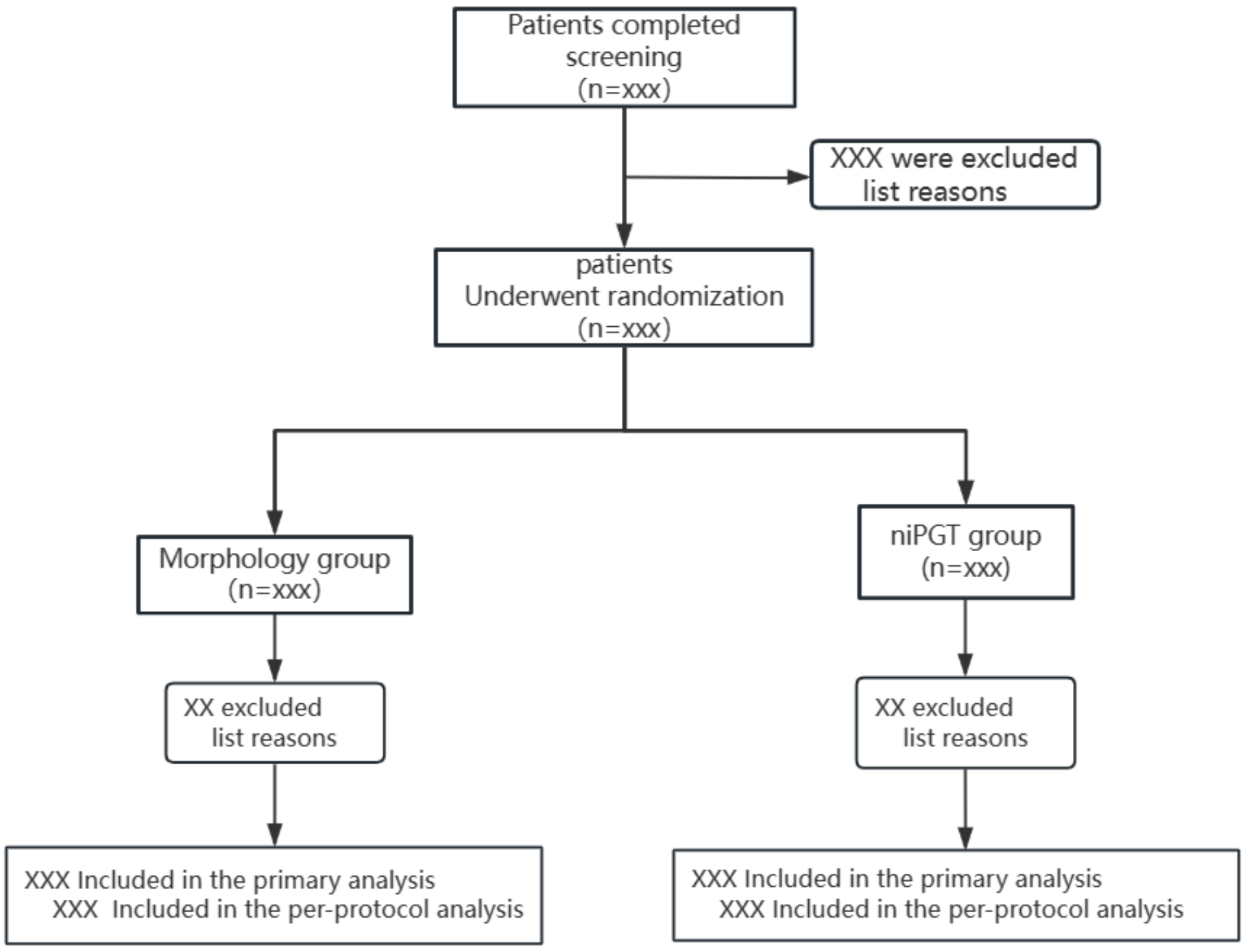

Table 1. Baseline characteristics

**Characteristics**	**niPGT group （n=XXX）**	**Morphology group （n=XXX）**
**Age-years**		
Female		
Male		
**Female BMI – kg/m2**		
**Duration of infertility - years**		
**Number of previous embryo transfers**		
0-1		
2		
≥3		

**Number of previous miscarriages**		
0		
1		
≥2		
**Previous live birth-yes no. (%)**		
**Primary infertility – no. (%)**		
**Indication for IVF**		
Female factor		
Male factor		
Couple		
**Laboratory testing (Female)**		
Basic follicle-stimulating hormone —IU/L		
Basic luteinizing hormone — IU/L		
Basic estradiol — pmol/L		
Basic progesterone— nmol/L		
**Endometrial thickness — mm**		
**COH treatment**		
GnRH-a protocol — no. (%)		
GnRH-ant protocol — no. (%)		
Other		
**No. of days of ovarian stimulation**		
**The total dose of follicle-stimulating hormone - IU**		
**Laboratory testing on the trigger day**		
Luteinizing hormone — IU/L		
Estradiol — pmol/L		
Progesterone — nmol/L		
**No. of oocytes retrieved**		
**No. of good-quality embryos on day 3**		
**No. of blastocysts**		
**Undergo embryo transfer, No. (%)**		
**Did not undergo embryo transfer, No. (%)**		

Table 2. Primary and secondary outcomes after the first embryo transfer cycle (intention-to-treat)

Outcome	Ni-PGT group(n=)	Morphology group(n=)	P value	Absolute difference (95%CI), %	Rate Ratio (95% CI)
**Primary outcome**					
Ongoing pregnancy					

rate					
**Secondary outcomes**					
Clinical pregnancy rate					
Early miscarriages					
Late miscarriages					
Live birth rate					

Table 3. Maternal and foetal/neonatal complications after the first embryo transfer cycle (intention-to-treat)

**Complication**	**Ni-PGT group(n=)**	**Morphology group(n=)**	**Absolute difference (95% CI)**	**Rate Ratio (95% CI)**	**P value**
**Maternal**					
Ectopic pregnancy					
Gestational diabetes					
Gestational hypertension					
Gestational diabetes mellitus combined hypertension					
Severe preeclampsia					
Premature rupture of membrane					
Cervical incompetence					
Very preterm birth (28~32 weeks)					
**Foetal/neonatal**					
Stillborn					
Congenital anomalies					
Very low birth weight(<1500g)					
Macrosomia (>4000g)					

5. References

1. Chen L, Li W, Liu Y, et al. Non-invasive embryo selection strategy for clinical IVF to avoid wastage of potentially competent embryos. Reprod Biomed Online 2022;45:26-34.
2. Zegers-Hochschild F, Adamson GD, Dyer S, et al. The International Glossary on Infertility and Fertility Care, 2017. Fertil Steril 2017;108:393-406.

Summary of changes to the Statistical Analysis Plan

Versions 1.0-2.0

Statistical Analysis Plan: Version 1.0 (June 21, 2021**)**

Statistical Analysis Plan: Version 2.0 (June 05, 2024**)**

Version 2.0 included additional analytical content:

1. **Primary and secondary outcomes between groups were compared using Poisson regression with robust SEs or a negative binomial regression model, adjusting for the centre, and presented as adjusted RRs and corresponding 95% CIs.**

2. **Subgroup analysis**
   Subgroup analyses will be performed according to female age(35, 36-37, 38-39 and 40-42 years old), the number of previous miscarriages(0, 1, ≥2), and the number of prior failed embryo transfer cycles(0-1, 2, ≥3).

3. **Results for the infertility core outcome set:**
   Standardizing definitions and reporting guidelines for the infertility core outcome set was published in December 2020[1], while our study started in April 2020. Therefore, we will present a supplementary table listing all core outcomes based on both intention-to-treat and per-protocol analyses.

References

1. Duffy JMN, AlAhwany H, Bhattacharya S, et al. Developing a core outcome set for future infertility research: an international consensus development study† ‡. Hum Reprod 2020;35:2725-34
