# Supplementary material for: Embryo selection by non-invasive preimplantation genetic testing for aneuploidy in women of advanced maternal age: a randomized, clinical trial
Source: Nat Commun. 2026 Sep 8;17:10634. doi: 10.1038/s41467-026-77628-4 (PMC13650144; doi:10.1038/s41467-026-77628-4)
Supplement: Supplementary file 2 [file 41467_2026_77628_MOESM2_ESM.pdf]

nature portfolio

Corresponding author(s): Jie Qiao and Ping Liu

Last updated by author(s): Jul 21, 2026

Reporting Summary

Nature Portfolio wishes to improve the reproducibility of the work that we publish. This form provides structure for consistency and transparency in reporting. For further information on Nature Portfolio policies, see our Editorial Policies and the Editorial Policy Checklist.

Statistics

For all statistical analyses, confirm that the following items are present in the figure legend, table legend, main text, or Methods section.

n/a	Confirmed	
☐	☒	The exact sample size (n) for each experimental group/condition, given as a discrete number and unit of measurement
☐	☒	A statement on whether measurements were taken from distinct samples or whether the same sample was measured repeatedly
☐	☒	The statistical test(s) used AND whether they are one- or two-sided<br>*Only common tests should be described solely by name; describe more complex techniques in the Methods section.*
☐	☒	A description of all covariates tested
☐	☒	A description of any assumptions or corrections, such as tests of normality and adjustment for multiple comparisons
☐	☒	A full description of the statistical parameters including central tendency (e.g. means) or other basic estimates (e.g. regression coefficient) AND variation (e.g. standard deviation) or associated estimates of uncertainty (e.g. confidence intervals)
☐	☒	For null hypothesis testing, the test statistic (e.g. F, t, r) with confidence intervals, effect sizes, degrees of freedom and P value noted<br>*Give P values as exact values whenever suitable.*
☒	☐	For Bayesian analysis, information on the choice of priors and Markov chain Monte Carlo settings
☒	☐	For hierarchical and complex designs, identification of the appropriate level for tests and full reporting of outcomes
☒	☐	Estimates of effect sizes (e.g. Cohen's d, Pearson's r), indicating how they were calculated

Our web collection on statistics for biologists contains articles on many of the points above.

Software and code

Policy information about availability of computer code

Data collection	All study data were collected and managed via a secure, web-based Electronic Data Capture (EDC) system(https://www.trialos.com.cn/). Spent culture medium(SCM) of all embryos in the 2 groups was collected for testing using non-invasive chromosome screening (NICS) technology. Whole-genome amplification and next-generation sequencing library preparation were performed on the collected SCMs using the NICSInst™ library kit (Xukang Medical Technology Co., Ltd., China). Pooled libraries were sequenced on the Illumina platform (Illumina Inc., USA), and approximately 2 M sequencing reads were obtained for each library.
Data analysis	Data were analyzed using ChromGo™ bioinformatics software (Xukang Medical Technology (suzhou) Co., Ltd).All statistical analyses were performed using SPSS version 25.0 (IBM SPSS Inc.). R software (version 4.3.3) was used for generating forest plots.

For manuscripts utilizing custom algorithms or software that are central to the research but not yet described in published literature, software must be made available to editors and reviewers. We strongly encourage code deposition in a community repository (e.g. GitHub). See the Nature Portfolio guidelines for submitting code & software for further information.

Data

Policy information about availability of data

All manuscripts must include a data availability statement. This statement should provide the following information, where applicable:

- Accession codes, unique identifiers, or web links for publicly available datasets
- A description of any restrictions on data availability
- For clinical datasets or third party data, please ensure that the statement adheres to our policy

The raw sequencing data generated in this study were stored at the National Genomics Data Center. Data are not publicly available owing to patient privacy and applicable data protection regulations but may be made available from the corresponding author (jie.qiao@263.net) upon reasonable request.
Individual de-identified participant data underlying the results reported in this study may be made available upon reasonable request by emailing the project lead investigator(jie.qiao@263.net). To facilitate this process, a formal proposal outlining the study's aims, the planned statistical analyses, publication plans, and distribution strategies is required.
Requests will be reviewed and responded to within 8 weeks of submission. The data-sharing request will be reviewed and approved by the principal investigator, the trial's management team, and the Medical Science Research Ethics Committee of Peking University Third Hospital, ensuring compliance with relevant laws and regulations in China.
Secondary users must agree not to attempt re-identification of participants and must cite the original trial (ClinicalTrials.gov: NCT04339166) and specify how their analyses differ from previously reported findings.
The trial's supporting documents, including the study protocol and the statistical analysis plan, have been compiled in the supplementary material.

Research involving human participants, their data, or biological material

Policy information about studies with human participants or human data. See also policy information about sex, gender (identity/presentation), and sexual orientation and race, ethnicity and racism.

Reporting on sex and gender	The study did not report on sex and gender.Our study exclusively involved female participants of advanced reproductive age (35-42years old)who underwent IVF treatment. Therefore, the findings apply to one sex (female), which is indicated in the title and abstract. Regarding embryo sex: Our niPGT-A assay detects copy number variations (CNVs) across all autosomes and sex chromosomes. However, in accordance with local regulations and clinical practice guidelines, we do not disclose embryonic sex information in our clinical reports. Instead, embryo ploidy status is reported "A.B.C" that does not reveal sex.No post hoc sex- or gender-based analyses were conducted, as the study was not designed or powered for such comparisons.
Reporting on race, ethnicity, or other socially relevant groupings	The study did not report on race, ethnicity,or other socially relevant groupings.
Population characteristics	We enrolled infertile couples who met the following characteisitics: (1) women aged 35-42 years; (2) women body mass index (BMI) ranging from 18 to 30 kg/m²; (3) having retrieved at least 6 oocytes; and (4) having at least 2 blastocysts with a morphology score of >4BC or 4CB.
Recruitment	Participants were consecutively screened by local investigators based on inclusion/exclusion criteria at participating IVF centers between April 2020 and June 2023. All eligible women were invited to participate during their routine consultation, and written informed consent was obtained from both partners. The final enrollment, however, was objectively determined by the availability of at least two blastocysts on day 5/6 with a morphology score of >4BC or 4CB., a biological criterion beyond the control of patients or investigators. Randomization was then performed only after this criterion was met. The voluntary nature of participation may introduce self-selection bias, as women who consented might differ from those who declined in terms of health-seeking behavior or willingness to participate in research. This could affect the generalizability of our findings to the broader infertile population. However, we believe the impact on the internal validity of the treatment comparison is minimal, because randomization was performed after oocyte retrieval and embryo confirmation, ensuring that both measured and unmeasured baseline characteristics were balanced between groups. This is supported by the well-balanced baseline characteristics shown in Table 1.
Ethics oversight	Ethical approval was obtained from the committees of all participating sites (including the Medical Science Research Ethics Committee of Peking University Third Hospital, the Ethics Committee of Nanjing Jinling Hospital, the Medical Ethics Committee of Hebei Maternity Hospital; Ethics Committee of Northwest Women's and Children's Hospital; Ethics Committee of Chinese PLA General Hospital; Ethics Committee of Shengjing Hospital of China Medical University; Ethics Committee of the First Affiliated Hospital of Sun Yet-Sen University; the ethical committee of the CITIC-Xiangya Reproductive & Genetic Hospital; the Ethics Committee of Peking University Shenzhen Hospital; the Ethics Committee of The Second Hospital of Hebei Medical University; the Ethics Committee of West China Second Hospital of Sichuan University the Ethics Committee of the Second Affiliated Hospital of Wenzhou Medical University; Ethics Committee of The Third Affiliated Hospital of Zhengzhou University).

Note that full information on the approval of the study protocol must also be provided in the manuscript.

Field-specific reporting

Please select the one below that is the best fit for your research. If you are not sure, read the appropriate sections before making your selection.

☒ Life sciences ☐ Behavioural & social sciences ☐ Ecological, evolutionary & environmental sciences

For a reference copy of the document with all sections, see nature.com/documents/nr-reporting-summary-flat.pdf

Life sciences study design

All studies must disclose on these points even when the disclosure is negative.

Sample size	This study was designed to achieve 90% power to detect an absolute difference of 10% in the ongoing pregnancy rate between the 2 groups, 38.8% in the morphology group, and a two-sided significance level of 0.05, requiring a minimum sample of 516 patients in each treatment group. We estimated a group sample size of 574 participants per arm to account for a dropout rate of 10%.
Data exclusions	The primary and secondary outcomes analysis was conducted according to the intention-to-treat principle. The primary statistical analysis was performed in a intention-to-treat population, containing all randomly assigned participants who experienced at least one intervention and had post-intervention evaluation data. We excluded couples who were found to be ineligible after randomization. These included: 11 participants who had only blastocyst and were mistakenly assigned , with no follow-up data available; 24 participants who withdrew consent and requested the transfer of two blastocysts; and 2 patients transferred embryos from other cycles and the embryo culture medium was not collected.
Replication	This is a prospectively designed randomized clinical trial. The methods of the trial have been described in detail in the manuscript and study protocol for replication.
Randomization	Eligible patients were randomized in a 1:1 ratio to the niPGT or morphology group.Randomization was performed using an online trial management system. When eligible patients had 2 or more blastocysts, the laboratory staff performed multicenter randomization using the system and assigned random numbers accordingly.
Blinding	This was a double-blind trial in which participants, clinicians, and nurses performing embryo transfers or follow-ups, as well as the investigators and assessors, were blinded to participants' allocation until the primary outcome occurred.

Reporting for specific materials, systems and methods

We require information from authors about some types of materials, experimental systems and methods used in many studies. Here, indicate whether each material, system or method listed is relevant to your study. If you are not sure if a list item applies to your research, read the appropriate section before selecting a response.

Materials & experimental systems

n/a	Involved in the study	
☐	☐	Antibodies
☐	☐	Eukaryotic cell lines
☐	☐	Palaeontology and archaeology
☐	☐	Animals and other organisms
☐	☒	Clinical data
☐	☐	Dual use research of concern
☐	☐	Plants

Methods

n/a	Involved in the study	
☐	☐	ChIP-seq
☐	☐	Flow cytometry
☐	☐	MRI-based neuroimaging

Antibodies

Antibodies used	*Describe all antibodies used in the study; as applicable, provide supplier name, catalog number, clone name, and lot number.*
Validation	*Describe the validation of each primary antibody for the species and application, noting any validation statements on the manufacturer's website, relevant citations, antibody profiles in online databases, or data provided in the manuscript.*

Eukaryotic cell lines

Policy information about cell lines and Sex and Gender in Research

Cell line source(s)	*State the source of each cell line used and the sex of all primary cell lines and cells derived from human participants or vertebrate models.*
Authentication	*Describe the authentication procedures for each cell line used OR declare that none of the cell lines used were authenticated.*
Mycoplasma contamination	*Confirm that all cell lines tested negative for mycoplasma contamination OR describe the results of the testing for mycoplasma contamination OR declare that the cell lines were not tested for mycoplasma contamination.*
Commonly misidentified lines (See ICLAC register)	*Name any commonly misidentified cell lines used in the study and provide a rationale for their use.*

Palaeontology and Archaeology

Specimen provenance	*Provide provenance information for specimens and describe permits that were obtained for the work (including the name of the issuing authority, the date of issue, and any identifying information). Permits should encompass collection and, where applicable, export.*
Specimen deposition	*Indicate where the specimens have been deposited to permit free access by other researchers.*
Dating methods	*If new dates are provided, describe how they were obtained (e.g. collection, storage, sample pretreatment and measurement), where they were obtained (i.e. lab name), the calibration program and the protocol for quality assurance OR state that no new dates are provided.*

☐ Tick this box to confirm that the raw and calibrated dates are available in the paper or in Supplementary Information.

Ethics oversight	*Identify the organization(s) that approved or provided guidance on the study protocol, OR state that no ethical approval or guidance was required and explain why not.*

Note that full information on the approval of the study protocol must also be provided in the manuscript.

Animals and other research organisms

Policy information about studies involving animals; ARRIVE guidelines recommended for reporting animal research, and Sex and Gender in Research

Laboratory animals	*For laboratory animals, report species, strain and age OR state that the study did not involve laboratory animals.*
Wild animals	*Provide details on animals observed in or captured in the field; report species and age where possible. Describe how animals were caught and transported and what happened to captive animals after the study (if killed, explain why and describe method; if released, say where and when) OR state that the study did not involve wild animals.*
Reporting on sex	*Indicate if findings apply to only one sex; describe whether sex was considered in study design, methods used for assigning sex. Provide data disaggregated for sex where this information has been collected in the source data as appropriate; provide overall numbers in this Reporting Summary. Please state if this information has not been collected. Report sex-based analyses where performed, justify reasons for lack of sex-based analysis.*
Field-collected samples	*For laboratory work with field-collected samples, describe all relevant parameters such as housing, maintenance, temperature, photoperiod and end-of-experiment protocol OR state that the study did not involve samples collected from the field.*
Ethics oversight	*Identify the organization(s) that approved or provided guidance on the study protocol, OR state that no ethical approval or guidance was required and explain why not.*

Note that full information on the approval of the study protocol must also be provided in the manuscript.

Clinical data

Policy information about clinical studies

All manuscripts should comply with the ICMJE guidelines for publication of clinical research and a completed CONSORT checklist must be included with all submissions.

Clinical trial registration	NCT04339166
Study protocol	The study protocol, including statistical analysis plan, is available in the Supplementary materials
Data collection	The data in this trial were collected using a Web-based data management system(https://www.trialos.com.cn/login/).The study

Data collection	enrolled participants between 16 April 2020 and 25 June 2023. Follow-up for the final randomized patient and other participants' delivery data was completed on August 6, 2024.
Outcomes	The primary outcome was ongoing pregnancy per the first planned blastocyst transfer, which was defined as a fetal heartbeat occurring 12 weeks after the blastocyst transfer. The ongoing pregnancy rate is defined as the number of women with an ongoing pregnancy divided by the number of women randomized to each group.<br>Secondary outcomes included clinical pregnancy, miscarriage, early miscarriage, and live birth. clinical pregnancy was defined as one or more observed gestational sacs or definitive clinical signs of pregnancy under ultrasonography at 7 weeks of gestation. Miscarriage was defined as a pregnancy that resulted in spontaneous abortion or therapeutic abortion that occurred throughout pregnancy before 28 weeks. First trimester miscarriage (early miscarriage) is defined as the spontaneous loss of an intrauterine pregnancy before 12 completed weeks of gestational age. Live birth was defined as the delivery of any viable neonate.

Dual use research of concern

Policy information about dual use research of concern

Hazards

Could the accidental, deliberate or reckless misuse of agents or technologies generated in the work, or the application of information presented in the manuscript, pose a threat to:

No	Yes	
☒	☐	Public health
☒	☐	National security
☒	☐	Crops and/or livestock
☒	☐	Ecosystems
☒	☐	Any other significant area

Experiments of concern

Does the work involve any of these experiments of concern:

No	Yes	
☒	☐	Demonstrate how to render a vaccine ineffective
☒	☐	Confer resistance to therapeutically useful antibiotics or antiviral agents
☒	☐	Enhance the virulence of a pathogen or render a nonpathogen virulent
☒	☐	Increase transmissibility of a pathogen
☒	☐	Alter the host range of a pathogen
☒	☐	Enable evasion of diagnostic/detection modalities
☒	☐	Enable the weaponization of a biological agent or toxin
☒	☐	Any other potentially harmful combination of experiments and agents

Plants

Seed stocks	*Report on the source of all seed stocks or other plant material used. If applicable, state the seed stock centre and catalogue number. If plant specimens were collected from the field, describe the collection location, date and sampling procedures.*
Novel plant genotypes	*Describe the methods by which all novel plant genotypes were produced. This includes those generated by transgenic approaches, gene editing, chemical/radiation-based mutagenesis and hybridization. For transgenic lines, describe the transformation method, the number of independent lines analyzed and the generation upon which experiments were performed. For gene-edited lines, describe the editor used, the endogenous sequence targeted for editing, the targeting guide RNA sequence (if applicable) and how the editor was applied.*
Authentication	*Describe any authentication procedures for each seed stock used or novel genotype generated. Describe any experiments used to assess the effect of a mutation and, where applicable, how potential secondary effects (e.g. second site T-DNA insertions, mosiacism, off-target gene editing) were examined.*

ChIP-seq

Data deposition

☐ Confirm that both raw and final processed data have been deposited in a public database such as GEO.

☐ Confirm that you have deposited or provided access to graph files (e.g. BED files) for the called peaks.

Data access links<br>*May remain private before publication.*	*For "Initial submission" or "Revised version" documents, provide reviewer access links. For your "Final submission" document, provide a link to the deposited data.*
Files in database submission	*Provide a list of all files available in the database submission.*
Genome browser session<br>(e.g. UCSC)	*Provide a link to an anonymized genome browser session for "Initial submission" and "Revised version" documents only, to enable peer review. Write "no longer applicable" for "Final submission" documents.*

Methodology

Replicates	*Describe the experimental replicates, specifying number, type and replicate agreement.*
Sequencing depth	*Describe the sequencing depth for each experiment, providing the total number of reads, uniquely mapped reads, length of reads and whether they were paired- or single-end.*
Antibodies	*Describe the antibodies used for the ChIP-seq experiments; as applicable, provide supplier name, catalog number, clone name, and lot number.*
Peak calling parameters	*Specify the command line program and parameters used for read mapping and peak calling, including the ChIP, control and index files used.*
Data quality	*Describe the methods used to ensure data quality in full detail, including how many peaks are at FDR 5% and above 5-fold enrichment.*
Software	*Describe the software used to collect and analyze the ChIP-seq data. For custom code that has been deposited into a community repository, provide accession details.*

Flow Cytometry

Plots

Confirm that:

☐ The axis labels state the marker and fluorochrome used (e.g. CD4-FITC).

☐ The axis scales are clearly visible. Include numbers along axes only for bottom left plot of group (a 'group' is an analysis of identical markers).

☐ All plots are contour plots with outliers or pseudocolor plots.

☐ A numerical value for number of cells or percentage (with statistics) is provided.

Methodology

Sample preparation	*Describe the sample preparation, detailing the biological source of the cells and any tissue processing steps used.*
Instrument	*Identify the instrument used for data collection, specifying make and model number.*
Software	*Describe the software used to collect and analyze the flow cytometry data. For custom code that has been deposited into a community repository, provide accession details.*
Cell population abundance	*Describe the abundance of the relevant cell populations within post-sort fractions, providing details on the purity of the samples and how it was determined.*
Gating strategy	*Describe the gating strategy used for all relevant experiments, specifying the preliminary FSC/SSC gates of the starting cell population, indicating where boundaries between "positive" and "negative" staining cell populations are defined.*

☐ Tick this box to confirm that a figure exemplifying the gating strategy is provided in the Supplementary Information.

Magnetic resonance imaging

Experimental design

Design type	*Indicate task or resting state; event-related or block design.*
Design specifications	*Specify the number of blocks, trials or experimental units per session and/or subject, and specify the length of each trial or block (if trials are blocked) and interval between trials.*
Behavioral performance measures	*State number and/or type of variables recorded (e.g. correct button press, response time) and what statistics were used to establish that the subjects were performing the task as expected (e.g. mean, range, and/or standard deviation across subjects).*

Acquisition

Imaging type(s) | Specify: functional, structural, diffusion, perfusion.

Field strength | Specify in Tesla

Sequence & imaging parameters | Specify the pulse sequence type (gradient echo, spin echo, etc.), imaging type (EPI, spiral, etc.), field of view, matrix size, slice thickness, orientation and TE/TR/flip angle.

Area of acquisition | State whether a whole brain scan was used OR define the area of acquisition, describing how the region was determined.

Diffusion MRI ☐ Used ☐ Not used

Preprocessing

Preprocessing software | Provide detail on software version and revision number and on specific parameters (model/functions, brain extraction, segmentation, smoothing kernel size, etc.).

Normalization | If data were normalized/standardized, describe the approach(es): specify linear or non-linear and define image types used for transformation OR indicate that data were not normalized and explain rationale for lack of normalization.

Normalization template | Describe the template used for normalization/transformation, specifying subject space or group standardized space (e.g. original Talairach, MNI305, ICBM152) OR indicate that the data were not normalized.

Noise and artifact removal | Describe your procedure(s) for artifact and structured noise removal, specifying motion parameters, tissue signals and physiological signals (heart rate, respiration).

Volume censoring | Define your software and/or method and criteria for volume censoring, and state the extent of such censoring.

Statistical modeling & inference

Model type and settings | Specify type (mass univariate, multivariate, RSA, predictive, etc.) and describe essential details of the model at the first and second levels (e.g. fixed, random or mixed effects; drift or auto-correlation).

Effect(s) tested | Define precise effect in terms of the task or stimulus conditions instead of psychological concepts and indicate whether ANOVA or factorial designs were used.

Specify type of analysis: ☐ Whole brain ☐ ROI-based ☐ Both

Statistic type for inference | Specify voxel-wise or cluster-wise and report all relevant parameters for cluster-wise methods.

(See Eklund et al. 2016)

Correction | Describe the type of correction and how it is obtained for multiple comparisons (e.g. FWE, FDR, permutation or Monte Carlo).

Models & analysis

n/a	Involved in the study
☐	☐ Functional and/or effective connectivity
☐	☐ Graph analysis
☐	☐ Multivariate modeling or predictive analysis

Functional and/or effective connectivity | Report the measures of dependence used and the model details (e.g. Pearson correlation, partial correlation, mutual information).

Graph analysis | Report the dependent variable and connectivity measure, specifying weighted graph or binarized graph, subject- or group-level, and the global and/or node summaries used (e.g. clustering coefficient, efficiency, etc.).

Multivariate modeling and predictive analysis | Specify independent variables, features extraction and dimension reduction, model, training and evaluation metrics.
