# Supplementary material for: Embryo selection by non-invasive preimplantation genetic testing for aneuploidy in women of advanced maternal age: a randomized, clinical trial
Source: Nat Commun. 2026 Sep 8;17:10634. doi: 10.1038/s41467-026-77628-4 (PMC13650144; doi:10.1038/s41467-026-77628-4)
Supplement: Supplementary file 3 [file 41467_2026_77628_MOESM3_ESM.pdf]

nature portfolio

Peer Review File

Embryo Selection by Non-invasive Preimplantation Genetic Testing for Aneuploidy in Women of Advanced Maternal Age: A Randomized, Clinical Trial

Corresponding Author: Professor Jie Qiao

This file contains all reviewer reports in order by version, followed by all author rebuttals in order by version.

Version 0:

Reviewer comments:

Reviewer #1

(Remarks to the Author)
This is a multicenter RCT on non invasive PGT-A vs morphology assessment in IVF. And in women with advanced maternal age, 35-42 years of age and using ranking of embryos instead of discarding. As such it is a very relevant trial. Primary outcome is ongoing pregnancy per randomized women. The paper is generally well written but there are certain confusing parts that need to be clarified. I have the following comments.
1. What is the primary outcome? In ms it is said to be ongoing pregnancy (defined as ongoing pregnancy at 12 gestational wks) per first embryo transfer. However, it should be ongoing pregnancy per first planned embryo transfer (or if you express it as per randomized woman). See Wilkinson Jack, Fertil Steril 2023. And so it is calculated in Results and in Table 2. The definition of primary outcome is more correctly given in the statistical protocol. However, at one place in the statistical protocol primary outcome is defined as “ongoing pregnancy leading to live birth” which is understood as live birth . This is extremely important to clarify.
2. In Introduction I think the Munne trial is incompletely cited. There was no significant difference in ongoing pregnancy rate in older women in the ITT analysis. The analysis per ET is of no or minimal use (as you see in Wilkinson paper) and this should be more clearly stated both in Introduction and Discussion.
3. You claim that your primary analysis is ITT, meaning including all randomized patients in the analysis, still 11 patients wishing double embryo transfer were randomized but excluded from ITT. Also 26 patients withdrawing informed consent were excluded. In my view your analysis is more a FAS analysis (full analysis set= an analysis where you have at least one outcome) which is ok for an RCT. In some way, you should explain this, eg. call it a modified ITT.
4. Live birth as primary outcome would have been superior to ongoing pregnancy rate. And should be stated as a limitation.
5. The time period is unclear to me. You state that the period patient were informed of the study was 25 April 2020 to 30 June 2023. Which period were recruitment/randomization period? Further you state in Statistical plan that the period for frozen embryo transfer was prolonged to 6 months past oocyte retrieval. And study ends 17 months after OPU. Anyhow, time has passed and by now all first embryo transfers ought to have been performed and it should be known if an ongoing pregnancy has occurred. Thus, possibility to update your results. If so, the best and worst scenarios are not relevant any longer.
6. Miscarriage is a complicated outcome since it can’t be evaluated without live birth. The (non) value of a decreased miscarriage rate should be discussed in view of no difference in live birth rate. Not referring to this trial, miscarriage rate in general could in fact be lower in the intervention group but at the same time the live birth rate could also be lower. Thus, showing superiority for miscarriage but inferiority for main outcome. I miss this discussion concerning miscarriage.
7. Another issue about miscarriage. Miscarriage can not be analyzed per ITT, since the denominator is clinical pregnancy. Just take out ITT here.
8. I understand and appreciate your ranking of embryos, instead of discarding but a few percent in both groups did not receive any embryo transfer. What were the exact reasons for no ET? Were also total aneuploid embryos ranked and not discarded?
9. Table 2. The title should be per planned embryo transfer. Which is correct and the way you have calculated your results. Both for ongoing pregnancy, clinical pregnancy and live birth.
10. I can’t find Figures in the files, particularly miss flow chart.

Reviewer #2

(Remarks to the Author)
the authors are to be congratulated on the effort of putting together a multi center study of amazing size involving 13 infertility clinics. Unfortunately, however, the - in doing so - have been ignoring some basic , by now indisputable facts, about preimplantation genetic diagnosis for aneuploidy (PGT-A). Let us start with what is likely the most important issue and that is that PGT-A simply does not work. As the ASRM in its most recent (September 2024) cCmmitteee Opinion very clearly concluded, PGT-A - in over 20 years of clinical use under different names- has been unable to establish even a single utility. Fairly, this judgment was reached based on invasive PGT-A through trophectoderm biopsy; but there is not a single study in the literature that has not shown superiority of non-invasive PGT-A over invasive PGT-A. To the contrary, almost every study on the subject demonstrated that non-invasive PGT-A did even worse than invasive PGT-A.
One, therefore, must question the whole purpose of the in this paper reported study because there is, simply no reason to expect that non-invasive PGT-A would improve IVF outcomes in comparison to non-PGT-A cycles (morphology only cycles).

There are, of course, additional questions concerning this study, such as differences in patient characteristics, stimulation protocols between patients and participating IV clinics, - but, once the basic design of a study makes no sense, everything else is, of course less important.

Reviewer #3

(Remarks to the Author)
The manuscript describes a randomized control trial examining the efficacy of embryo selection via non-invasive preimplantation genetic testing (niPGT) compared to selection using morphology grade alone among advanced aged patients in the setting of IVF. Patients were randomly assigned to embryo selection using either niPGT or morphology/control, then compared based on the primary outcome of ongoing pregnancy. Several other outcomes are considered as secondary. The trial used a multicenter, double-blind randomization design with two comparison arms. The study is rigorous and well-designed overall.
My comments and questions for the authors are listed below:
1. In the abstract, results, and (especially) conclusion sections there is a consistent overinterpretation of the study's null result found in the primary analysis. The study was designed and powered using a superiority hypothesis (i.e. testing if the clinical pregnancy rate with niPGT is higher (or different) than in the control/morphology alone), not a non-inferiority hypothesis (testing if niPGT is at least as effective compared as the control) or an equivalence hypothesis (testing if niPGT and the control have a negligible difference in efficacy). The interpretation of the null results, however, is written more as if this study used a non-inferiority or equivalence hypothesis because the lack of a statistically significant difference between niPGT and the control is interpreted as definitive proof that there is no difference. This is not consistent with the hypothesis that the study was powered for, and tested, and should be corrected to instead interpret the results as "insufficient proof to establish a statistically significant difference between the clinical pregnancy rates within the two treatment arms".
2. The results comparing the niPGT vs control arms do not show statistically significant differences in outcomes, but one way to additionally compare these two methods would be to examine how often the two methods would have selected the same embryo. This is possible because patients in the niPGT group also received morphology rankings, so it is theoretically possible to compare the proportion of cases where the two methods selected the same embryo. If niPGT and morphology are consistently suggesting the same embryo selection decisions this provides further evidence that they are functionally similar. If the two methods select the same embryo, the majority of the time, this also gives insight into why the study failed to detect a difference in clinical pregnancy between the 2 groups.
3. Not directly describing the analytic methodology used within the paper caused some confusion. It would perhaps be helpful to at least briefly clarify what analyses were implemented and how they were designed within the paper itself to make the results section easier to follow. Some aspects that were particularly hard to follow without an in-text methods description include: identifying which covariates have been included in adjusted analyses, sensitivity analyses, post-hoc analyses, distinction between intent-to-treat vs per-protocol analyses, distinction between pre-planned vs post-hoc analyses. Please consider what information in the results needs further detail given that the methods are described only in the supplemental data section.
4. The online methods section specifies that "When blastocysts had the same NICS grade, those with a higher morphological grade were preferentially transferred" it might be relevant to clarify how often this occurred, since if it is a frequent occurrence the differentiation between treatment groups could become less definitive. This also relates to item #2 above.
5. Minor point: in the abstract (line 80) there is a typo in the sentence "No statistically differences were observed…" I believe this should instead read "No statistically significant differences were observed…"
6. Both the results figures (labeled as Figure 1 and Figure 2) appear to have been omitted for some reason, although the figure legends are included within the manuscript PDF.

Reviewer #4

(Remarks to the Author)
This is a prospective double-blind, randomized controlled trial to evaluate the value of niPGT aneuploidy screening to improve the ongoing pregnancy rate and live birth rates in women aged 35-42. niPGT is rather novel and the clinical validity and utility of the method remains still questionable. In addition, there is an ongoing debate about the clinical validity and utility of PGT-A in general, independent of whether the methods used are invasive or non-invasive. Hence studies about this topic are timely and required. This study is at scale, from a clinical study perspective well executed, and hence provides valuable data for the field. Nevertheless, there remains ambiguity about the overall outcome and broader value of the study

(see major comments below):
Major comments:

- The authors write in the discussion line 244: “The SCM cfDNA content is extremely low and the components are complex. Therefore, at present, niPGT has not yet reached an accurate level.” This statement begs the question: how good is the technical accuracy of niPGT of this team: In the absence of a good estimate of the technical accuracy in this specific study, the clinical outcomes may not be generalized. It is possible that the technology used by this team does not capture all aneuploidies and hence results in equal implantation and baby take home rates in both legs? If so, the study merely shows that niPGT (as performed by this consortium) does not allow for stratification of embryos but does not address the broader question to what extent aneuploidy screening is or is not valuable to improve ongoing pregnancy rate and live birth rates.
- The results of the aneuploidy screening are not shown. It is not mentioned how many embryos were selected against and what the incidence of aneuploidy detected in this study was. It would be valuable to see the distribution of the aneuploidies detected and selected against. In addition, the authors refer to a classification in A, B and C embryos. It remains unclear how many were classified as A, B and C and how many A or B (which can be also no-result in the niPGT group) were transferred. It would also be valuable to see those results/age group. Those values are essential to (1) evaluate the accuracy of the niPGT based on comparison with epidemiological/reference data, (2) to understand the selection and potential tranfer biases. Specifically required are (a) Embryo classification by preimplantation genetic screening, number of euploid/aneuploid embryos, number of monosomies, trisomies, mosaics, segmental and potential more complex outcomes and classifications (A,B,C).
- I am confused about the outcome: On the one hand, most major intention to treat parameters did not show a significant change: ongoing pregnancy rate, clinical pregnancy, late miscarriage and baby take home rate. However, early miscarriage rate did show a significant difference: in the niPGT group there were significant less miscarriages. It is surprising that this difference does not percolate into the live birth rates. In light of this question, it would be valuable to see the breakdown numbers of miscarriages in the different age groups.
- I cannot find the two figures referred to in the text and figure legends.
Minor comments
- The overall success rate for the morphology graded group seems higher than expected based on epidemiologically evaluation. Especially for the >38 years of age group, this number is likely lower. It would be good to provide those breakdown numbers.
- Discussion line 260:”In our study, patients with 2 or more failed transfer cycles showed no significant differences in clinical outcomes between the 2 groups, suggesting that their implantation failure involves more complex reasons beyond embryo selection.” I am confused since in the results only the outcome after the first embryo transfer are discussed. Where is this statement coming from?
- In introduction and discussion: the potential detrimental effect of embryo biopsy is over represented. There are other studies suggesting that biopsy is not detrimental or only when not applied properly. Those studies could also be referred to.
- Title: ‘Non-invasive Preimplantation Genetic Testing’ is too broadly scoped: This study is not about all forms of NIPT, but specifically about aneuploidy screening. The title should be reworded to ‘non-invasive PGT for aneuploidy detection’.
- The authors often use the term ploidy (e.g. first sentce of abstract). The term ploidy is specifically used to iondicate the number of genomes, e.g. haploid, diploid, triploid, tetraploid while here aneuploidies, chromomal copy number changes are investigated. Change ‘embryos’ ploidy’ to ‘chromosomal aneuploidy in embryos’.

Reviewer #5

(Remarks to the Author)
I co-reviewed this manuscript with one of the reviewers who provided the listed reports. This is part of the Nature Communications initiative to facilitate training in peer review and to provide appropriate recognition for Early Career Researchers who co-review manuscripts.

Version 1:

Reviewer comments:

Reviewer #1

(Remarks to the Author)
The paper has improved and most questions have been answered in a satisfactory way. However there are some parts which are not correct or confusing. I list them here:
1.In Introduction, the following part is not correct:
"For example, a study by Rubio et al7. showed that in women between 38 and 41 years, the delivery rate after the first transfer attempt was significantly higher in the PGT-A group per transfer (52.9% vs 24.2%) and per patient (36.0% vs. 21.9%). In a trial by Munne et al., a post hoc analysis indicated a higher ongoing pregnancy rate per embryo transfer in women aged 35–40 years with PGT-A, although no significant difference was found in the primary intention-to-treat analysis for this subgroup 9".

The Rubio trial is very difficult to read and digest and important parts are more or less "hidden". There is no significant improvement in live birth in the PGT-A arm in the ITT analysis (only given in text, not Tables). The Sacchi paper 2019 is a retrospective study and only reporting results per embryo transfer. However, here are three published RCT in older women, not showing any improvement in live birth by PGT-A (Rubio-2017, Munne-2019, Verpoest-2018). This part of your ms should be revised.

2.Line 178. I presume you mean embryo transfer? you write transplantation?
3.Line 352. I presume you mean PGT-SR and PGT-M. Please clarify.
4.Line 444. Add that this is a superiority trial, aiming to find a 10% higher ong PR in the PGT-A. arm.
5. I am still confused about the Flow chart and how 38 and 32 patients in the the mid box are managed. They are all included in the modified ITT (primary analysis). 21+19 did not receive embryo transfer. Were these women then calculated as negative pregnancy? or ? All other women in these boxed received embryo transfer I presume? and you include the results from these transfers in your primary analysis. Please clarify this part as being an extremely important question.

Reviewer #2

(Remarks to the Author)
None

Reviewer #3

(Remarks to the Author)
The Authors have addressed my concerns adequately.

Reviewer #4

(Remarks to the Author)
The authors addressed most of my comments. However:
1. The authors should add the analysis of their abstract at the ESHRE 2025 (Poster number:P-094: https://doi.org/10.1093/humrep/deaf097.403) into the manuscript. This analysis provides interesting insights into the potential value of PGT-A and ways forward for potential future studies. I see no reason not to incorporate those findings into the current manuscript.
2. The results of the aneuploidy screening are not shown. A. It is not mentioned how many embryos were selected against and what the incidence of aneuploidy detected in this study was. It would be valuable to see the distribution of the aneuploidies detected
and selected against. The authors did not yet answer this question. The answer to this question allows to assess the accuracy of the method, by comparing the epidemiological data with known/published incidences.
3. B. It remains unclear how many were classified as A, B and C and how many A
or B (which can be also no-result in the niPGT group) were transferred. The authors write that in the manuscript, only the results of the first planned embryo were focused. Moreover, they state that they examined the SCM samples of all the blastocysts and anticipate that more findings will be presented in further paper. I do not understand why they cannot bring those results into this article. I do believe that this information would strengthen the article and provide a more comprehensive view of the results.

Reviewer #5

(Remarks to the Author)
I co-reviewed this manuscript with one of the reviewers who provided the listed reports. This is part of the Nature Communications initiative to facilitate training in peer review and to provide appropriate recognition for Early Career Researchers who co-review manuscripts.

Version 2:

Reviewer comments:

Reviewer #1

(Remarks to the Author)
I am satisfied with the answers from the authors. On page 452 you should however change to embryo transfer (not transplantation).
I miss the revised flow chart.

Reviewer #4

(Remarks to the Author)
The authors addressed my comments.

Open Access This Peer Review File is licensed under a Creative Commons Attribution 4.0 International License, which permits use, sharing, adaptation, distribution and reproduction in any medium or format, as long as you give appropriate credit to the original author(s) and the source, provide a link to the Creative Commons license, and indicate if changes were made.
In cases where reviewers are anonymous, credit should be given to 'Anonymous Referee' and the source.
The images or other third party material in this Peer Review File are included in the article's Creative Commons license, unless indicated otherwise in a credit line to the material. If material is not included in the article's Creative Commons license and your intended use is not permitted by statutory regulation or exceeds the permitted use, you will need to obtain permission directly from the copyright holder.
To view a copy of this license, visit https://creativecommons.org/licenses/by/4.0/

Title: Embryo Selection by Non-invasive Preimplantation Genetic Testing for Aneuploidy in Advanced Aged Women: A Randomized, Clinical Trial

Submission ID: NCOMMS-25-75661-T

We gratefully appreciate the comments from all Reviewers. In response, we have made extensive modifications to the manuscript, which are marked in red in the resubmitted files. Additionally, we have provided detailed, point-by-point responses to each of the reviewers' comments. You can find these responses below, and the revisions are included in the resubmitted files.

Response to Reviewer's Comments:

Reviewer #1 (Remarks to the Author):

This is a multicenter RCT on non-invasive PGT-A vs morphology assessment in IVF. And in women with advanced maternal age, 35-42 years of age and using ranking of embryos instead of discarding. As such it is a very relevant trial. Primary outcome is ongoing pregnancy per randomized women. The paper is generally well written but there are certain confusing parts that need to be clarified. I have the following comments.

Response: Thank you very much for your positive comments. According to your nice suggestions, we have made the responses and revisions. Shown below.

1. What is the primary outcome? In ms it is said to be ongoing pregnancy (defined as ongoing pregnancy at 12 gestational wks) per first embryo transfer. However, it should be ongoing pregnancy per first planned embryo transfer (or if you express it as per randomized woman). See Wilkinson Jack, Fertil Steril 2023. And so it is calculated in Results and in Table 2. The definition of primary outcome is more correctly given in the statistical protocol. However, at one place in the statistical protocol primary outcome is defined as "ongoing pregnancy leading to live birth" which is understood as live birth. This is extremely important to clarify.

Response: Thank you for your comment. I am sorry that this part was not clear in the original manuscript. We have revised the description of the primary outcome to be "ongoing pregnancy per the first planned embryo transfer." (highlighted revision, Page 6, lines 141-142.)

We have carefully reviewed the statistical analysis plan. To avoid ambiguity, we

have revised the description of "ongoing pregnancy leading to live birth" to "The primary outcome, ongoing pregnancy" (Supplemetary Study Protocol: P15, highlighted in red) to ensure it is fully consistent with the definition in the main text.

2. In Introduction I think the Munne trial is incompletely cited. There was no significant difference in ongoing pregnancy rate in older women in the ITT analysis. The analysis per ET is of no or minimal use (as you see in Wilkinson paper) and this should be more clearly stated both in Introduction and Discussion.

Response: We have revised the relevant sentence in the Introduction sections to provide an accurate representation of the Munne et al. trial findings. On Page 4, lines 99-101, highlighted revision. We have carefully reviewed all content in the Discussion section to ensure such errors are avoided.

3. You claim that your primary analysis is ITT, meaning including all randomized patients in the analysis, still 11 patients wishing double embryo transfer were randomized but excluded from ITT. Also 26 patients withdrawing informed consent were excluded. In my view your analysis is more a FAS analysis (full analysis set= an analysis where you have at least one outcome) which is ok for an RCT. In some way, you should explain this, eg. call it a modified ITT.

Response: We agree that our primary analysis more closely aligns with the definition of a "full analysis set" or a "modified intention-to-treat analysis." We have revised the "Study Participants" (highlighted revision, Page 6, line 133) and "Statistical Analysis" (highlighted revision, Page 20, line 451-454) section of the manuscript to describe our primary analysis accurately.

4. Live birth as primary outcome would have been superior to ongoing pregnancy rate. And should be stated as a limitation.

Response: Thank you for your suggestion. We have added a statement to the Limitations section. (highlighted revision, Page 14, line 315-318).

5. The time period is unclear to me. You state that the period patient were informed of the study was 25 April 2020 to 30 June 2023. Which period were recruitment/randomization period? Further you state in Statistical plan that the period for frozen embryo transfer was prolonged to 6 months past oocyte retrieval. And study ends 17 months after OPU. Anyhow, time has passed and by now all first embryo transfers

ought to have been performed and it should be known if an ongoing pregnancy has occurred. Thus, possibility to update your results. If so, the best and worst scenarios are not relevant any longer.

Response: I am sorry that this part was not clear in the original manuscript. We have added the descriptions of the relevant timelines in the Study Participants and Methods section（highlighted revision, Page 5, lines 127-128; Page16, lines 361-367）.

This study was initiated with recruitment on April 16, 2020, with the first patient randomized on April 25, 2020, and the last patient randomized on June 25, 2023. According to the study protocol, the planned endpoint was defined as 17 months after the oocyte retrieval of the last randomized patient. The trial completion endpoint for the entire clinical study was set for November 25, 2024. Actually, the last randomized patient completed the first embryo transfer on November 21, 2023, and follow-up of delivery data for all randomized patients was completed on August 6, 2024, marking the completion of the entire clinical trial.

At trial completion, a total of 40 patients had not undergone their first planned transfer in this oocyte retrieval cycle. As you know, the COVID-19 pandemic occurred during the study period, with multiple factors affecting patients' choices of their strategies

The reasons are detailed in the table below. Given that the vast majority of patients will not undergo transplantation, we currently plan to discontinue follow-up updates on patient outcomes.

Reasons	Patients Number	
	NiPGT-A group	Morphology group
Spontaneous pregnancy	3	3
Divorce	1	1
The patient made the decision to forgo the embryo transfer.	0	1
Withdrew： Performed PGT-A	2	2
Subject requests to withdraw from the study	4	2
Lost to follow-up	8	6
The physical condition is not suitable for embryo transfer： Hypertension or Hypotension	1	2
Did not perform embryo transfer before the deadline	2	2
Total	21	19

6. Miscarriage is a complicated outcome since it can't be evaluated without live birth. The (non) value of a decreased miscarriage rate should be discussed in view of no difference in live birth rate. Not referring to this trial, miscarriage rate in general could in fact be lower in the intervention group but at the same time the live birth rate could also be lower. Thus, showing superiority for miscarriage but inferiority for main outcome. I miss this discussion concerning miscarriage.

Response: Thank you for this very insightful comment. We agreed with your opinion.

As you know, the denominator of the live birth rate is number of randomized women, while that of the miscarriage rate is number of clinical pregnant.

Our results showed superiority in reducing early miscarriage rate but no differences in primary outcomes. This discrepancy may be attributable to the limited sample size and potential selection bias.

Further research should both expand the sample size and focus on identifying which subgroup of patients will benefit most from niPGT-A, to provide more targeted clinical guidance.

We reviesed it in the Discussion, highlighted revision, Page 13, lines 292-298.

7. Another issue about miscarriage. Miscarriage can not be analyzed per ITT, since the denominator is clinical pregnancy. Just take out ITT here.

Response: We are very sorry for this error and have corrected it in the article. (highlighted revision, Page 7, lines 153-157).

8. I understand and appreciate your ranking of embryos, instead of discarding but a few percent in both groups did not receive any embryo transfer. What were the exact reasons for no ET? Were also total aneuploid embryos ranked and not discarded?

Response: Thank you for this comment. At trial completion, a total of 40 patients had not undergone their first planned transfer in this oocyte retrieval cycle. The reasons are detailed in the table below. In this study, we used niPGT-A as the screening technique to rank embryos based on their probability of being euploid. All embryos, including those detected as aneuploid (NICS result is grade C), had the opportunity to be transferred.

Reasons	Patients Number	
	NiPGT-A group	Morphology group
Spontaneous pregnancy	3	3
Divorce	1	1
The patient made the decision to forgo the	0	1

embryo transfer.		
Withdrew： Performed PGT-A	2	2
Subject requests to withdraw from the study	4	2
Lost to follow-up	8	6
The physical condition is not suitable for embryo transfer： Hypertension or Hypotension	1	2
Did not perform embryo transfer before the deadline	2	2
Total	21	19

9. Table 2. The title should be per planned embryo transfer. Which is correct and the way you have calculated your results. Both for ongoing pregnancy, clinical pregnancy and live birth.

Response: Thank you for this comment. We agree with your point and have updated all the corresponding description in the title, including the supplementary table titles.

10. I can't find Figures in the files, particularly miss flow chart.

Response: We apologize for the inconvenience caused by the missing figure files. We have now uploaded the complete set of figures (including the flow chart) to the submission system.

Reviewer #2 (Remarks to the Author):

the authors are to be congratulated on the effort of putting together a multi center study of amazing size involving 13 infertility clinics. Unfortunately, however, the - in doing so - have been ignoring some basic, by now indisputable facts, about preimplantation genetic diagnosis for aneuploidy (PGT-A). Let us start with what is likely the most important issue and that is that PGT-A simply does not work. As the ASRM in its most recent (September 2024) Committee Opinion very clearly concluded, PGT-A - in over 20 years of clinical use under different names- has been unable to establish even a single utility. Fairly, this judgment was reached based on invasive PGT-A through trophectoderm biopsy; but there is not a single study in the literature that has not shown superiority of non-invasive PGT-A over invasive PGT-A. To the contrary, almost every study on the subject demonstrated that non-invasive PGT-A did even worse than invasive PGT-A.
One, therefore, must question the whole purpose of the in this paper reported study because there is, simply no reason to expect that non-invasive PGT-A would improve IVF

outcomes in comparison to non-PGT-A cycles (morphology only cycles).

There are, of course, additional questions concerning this study, such as differences in patient characteristics, stimulation protocols between patients and participating IV clinics, - but, once the basic design of a study makes no sense, everything else is, of course less important.

Response: Thank you very much for reviewing our manuscript and providing such valuable and constructive feedback.

PGT has a 30-year development history. While numerous researches including many randomized controlled trials (RCTs) have been conducted to evaluate its clinical efficacy, the technology remains a contentious topic to date.

NiPGT has a development history of less than a decade and is already recognized as one of the most impactful emerging technologies in ART. However, the evidence for its clinical efficacy is insufficient.

This is the first RCT to examine whether niPGT is an effective tool for evaluating embryos. The negative result from this study is very important, and will be critical for providing guidance for the use of niPGT-A. We are confident that our trial will lay a solid foundation for the long-term, healthy development of this technology. Moreover, we introduced a novel strategy for ranking embryos rather than discarding the aneuploidy embryos.

Reviewer #3 (Remarks to the Author):

The manuscript describes a randomized control trial examining the efficacy of embryo selection via non-invasive preimplantation genetic testing (niPGT) compared to selection using morphology grade alone among advanced aged patients in the setting of IVF. Patients were randomly assigned to embryo selection using either niPGT or morphology/control, then compared based on the primary outcome of ongoing pregnancy. Several other outcomes are considered as secondary. The trial used a multicenter, double-blind randomization design with two comparison arms. The study is rigorous and well-designed overall.

Response: Thank you for your kind feedback. We have carefully reviewed your suggestions and made the corresponding revisions, as detailed below.

My comments and questions for the authors are listed below:

1. In the abstract, results, and (especially) conclusion sections there is a consistent overinterpretation of the study's null result found in the primary analysis. The study was designed and powered using a superiority hypothesis (i.e. testing if the clinical pregnancy

rate with niPGT is higher (or different) than in the control/morphology alone), not a non-inferiority hypothesis (testing if niPGT is at least as effective compared as the control) or an equivalence hypothesis (testing if niPGT and the control have a negligible difference in efficacy). The interpretation of the null results, however, is written more as if this study used a non-inferiority or equivalence hypothesis because the lack of a statistically significant difference between niPGT and the control is interpreted as definitive proof that there is no difference. This is not consistent with the hypothesis that the study was powered for, and tested, and should be corrected to instead interpret the results as "insufficient proof to establish a statistically significant difference between the clinical pregnancy rates within the two treatment arms".

Response: Thank you for this comment. We have revised and updated the paragraphs in the abstract, results, and conclusion sections, with the modified portions highlighted in red, highlighted revision, on pages 3, lines 78-80；Page 6, lines 144-146； Page 14, lines 323.

2. The results comparing the niPGT vs control arms do not show statistically significant differences in outcomes, but one way to additionally compare these two methods would be to examine how often the two methods would have selected the same embryo. This is possible because patients in the niPGT group also received morphology rankings, so it is theoretically possible to compare the proportion of cases where the two methods selected the same embryo. If niPGT and morphology are consistently suggesting the same embryo selection decisions this provides further evidence that they are functionally similar. If the two methods select the same embryo, the majority of the time, this also gives insight into why the study failed to detect a difference in clinical pregnancy between the 2 groups.

Response: Thank you for your valuable comment.

At your suggestion, we added 2 paragraphs as follow:

Another explanation for the negative outcomes between the two groups may be that the two methods would have selected embryos of the same quality. In the niPGT-A group, the embryo scheduled for transfer might have been the one with both the optimal NICS result and superior morphological grade. In the morphology group, the embryo planned for transfer was selected based on morphological assessment, which might have been an euploid embryo. (highlighted revision, Page 12, lines 269-273)

When stratified analysis in the niPGT-A group, a total of 550 blastocysts were

transferred, including 92% of grade A, B blastocysts (506/550) and only 8% of grade C blastocysts (44/550). Statistically significant differences were observed between the blastocyst subgroup with NICS grades A+B and that with NICS grade C regarding clinical pregnancy rate, early miscarriage rate, ongoing pregnancy rate and live birth rate (all p<0.05). (Supplementary Table 9). (highlighted revision, Page 9, lines 201-205)

We fully agree with your perspective that analyzing the concordance between selection methods is crucial for interpreting the trial results. To explore this further, we conducted an additional study. This work was presented in an abstract at the ESHRE 2025 (Poster number:P-094: https://doi.org/10.1093/humrep/deaf097.403).

In the subgroup of patients with only poor-quality morphology embryos, the differences were more pronounced: the niPGT group showed a 27.5% reduction in early miscarriage rate(p=0.025) and a 9.8% increase in ongoing pregnancy rate(p=0.185). The latter result, possibly due to the small sample size, did not reach statistical significance (Table 1).

Among the patients who had moderate- or low-grade morphology embryos, the miscarriage rate was significantly lower in the niPGT group (19.0% vs 29.5%, p=0.037), the ongoing pregnancy rate was higher(39.4% vs 31.8%, p=0.049)(Table 2).

Among the patients with good-grade morphology blastocysts, there were no significant differences between the niPGT and morphology groups(Table 3)

Table 1 Comparison outcomes of first embryo transfer in patients with only low-grade embryos

Outcomes	NICS: A+B	Morphology group	Absolute difference	p-value
Clinical pregnancy rate	36.9% (24/65)	37.5% (30/80)	-0.6%	0.943
Early miscarriage rate	12.5% (3/24)	40.0% (12/30)	**-27.5%**	**0.025**
Ongoing pregnancy rate	32.3% (21/65)	22.5% (18/80)	9.80%	0.185
Live birth rate	32.3% (21/65)	22.5% (18/80)	9.80%	0.185

Table 2 Comparison outcomes of first embryo transfer in patients with moderate- and low-embryos (20.2% +41.8 %).

Outcomes	NICS: A+B	Morphology group	Absolute difference	p-value
Clinical pregnancy rate	48.6% (142/292)	45.2% (149/330)	3.4%	0.386
Early miscarriage rate	19.0% (27/142)	29.5% (44/149)	**-10.5%**	**0.037**
Ongoing pregnancy rate	39.4% (115/292)	31.8% (105/330)	**7.6%**	**0.049**
Live birth rate	37.3% (109/292)	31.2% (103/330)	6.1%	0.108

Table 3 Comparison outcomes of first embryo transfer in patients with ≥1 good-embryos (22.2%+15.3%)

Outcomes	NICS: A+B	Morphology group	Absolute difference	p-value
Clinical pregnancy rate	55.6% (119/214)	59.4% (139/234)	-3.8%	0.417
Early miscarriage rate	14.3% (17/119)	20.9% (29/139)	-6.6%	0.169
Ongoing pregnancy rate	47.7% (102/214)	47.0% (110/234)	0.7%	0.890
Live birth rate	45.8% (98/214)	44.4% (104/234)	1.3%	0.774

3. Not directly describing the analytic methodology used within the paper caused some confusion. It would perhaps be helpful to at least briefly clarify what analyses were implemented and how they were designed within the paper itself **to make the results section easier to follow.** Some aspects that were particularly hard to follow without an in-text methods description include: identifying which covariates have been included in adjusted analyses, sensitivity analyses, post-hoc analyses, distinction between intent-to-treat vs per-protocol analyses, distinction between pre-planned vs post-hoc analyses. Please consider what information in the results needs further detail given that the methods are described only in the supplemental data section.

Response: I am sorry that this part was not clear in the original manuscript. We have updated the Results section in response to your valuable feedback. When reporting specific results thereafter, we have included inline notes to immediately indicate the analytical methods and adjustments applied to each finding, ensuring that readers can clearly follow the analytical logic and interpretation of the results.

4. The online methods section specifies that "When blastocysts had the same NICS grade, those with a higher morphological grade were preferentially transferred" it might be relevant to clarify how often this occurred, since if it is a frequent occurrence the differentiation between treatment groups could become less definitive. This also relates to item #2 above.

Response: We thank you for pointing this out. We added 2 paragraphs in the revision as answered above.

In real clinical practice, morphological evaluation is widely used. There is a possibility that an embryo with a higher morphological grade may itself be an embryo with a higher NICS grade.

5. Minor point: in the abstract (line 80) there is a typo in the sentence "No statistically differences were observed…" I believe this should instead read "No statistically significant differences were observed…"

Response: We apologize for this error and have corrected it in the article. (highlighted revision, Page 3, lines 79-80).

6. Both the results figures (labeled as Figure 1 and Figure 2) appear to have been omitted for some reason, although the figure legends are included within the manuscript PDF.

Response: We apologize for the inconvenience caused by the missing figure files. The figures have now been uploaded to the submission system as supplementary material.

Reviewer #4 (Remarks to the Author):

This is a prospective double-blind, randomized controlled trial to evaluate the value of niPGT aneuploidy screening to improve the ongoing pregnancy rate and live birth rates in women aged 35-42. niPGT is rather novel and the clinical validity and utility of the

method remains still questionable. In addition, there is an ongoing debate about the clinical validity and utility of PGT-A in general, independent of whether the methods used are invasive or non-invasive. Hence studies about this topic are timely and required. This study is at scale, from a clinical study perspective well executed, and hence provides valuable data for the field. Nevertheless, there remains ambiguity about the overall outcome and broader value of the study (see major comments below):

Response: Thank you very much for your positive comments. According to your nice suggestions, we have made the responses and revisions, and shown below.

Major comments:

- The authors write in the discussion line 244: "The SCM cfDNA content is extremely low and the components are complex. Therefore, at present, niPGT has not yet reached an accurate level." This statement begs the question: how good is the technical accuracy of niPGT of this team: In the absence of a good estimate of the technical accuracy in this specific study, the clinical outcomes may not be generalized. It is possible that the technology used by this team does not capture all aneuploidies and hence results in equal implantation and baby take-home rates in both legs? If so, the study merely shows that niPGT (as performed by this consortium) does not allow for stratification of embryos but does not address the broader question to what extent aneuploidy screening is or is not valuable to improve ongoing pregnancy rate and live birth rates.

Response: Thank you for this very insightful comment. We fully agree that the technical accuracy of the specific niPGT platform used in any study is paramount and directly impacts the generalizability of clinical outcomes. We appreciate the opportunity to clarify this crucial point.

The niPGT-A platform (NICS) employed in our study is a well-validated screening tool. We have conducted a lot of researches using this technology platform, such as Ref. 25, 26, 32, 36 and so on. In this study, aneuploidy screening was detected by this technology platform. As we mentioned in the manuscript, Page 9，lines 201-205. "When stratified analysis in the niPGT-A group, a total of 550 blastocysts were transferred, including 92% of grade A, B blastocysts (506/550) and only 8% of grade C blastocysts (44/550). Statistically significant differences were observed between the blastocyst subgroup with NICS grades A+B and that with NICS grade C regarding clinical pregnancy rate, early miscarriage rate, ongoing pregnancy rate and live birth rate (all p<0.05). (Supplementary Table 9)."

NiPGT-A has not yet reached an accurate level at present. In this trial, we introduced a novel strategy for ranking embryos rather than discarding the aneuploidy embryos. In the manuscript，we also mentioned that future research

should focus on the accuracy of the SCM cfDNA detection technology. However, it is not our main aim of this trial.

- The results of the aneuploidy screening are not shown. It is not mentioned how many embryos were selected against and what the incidence of aneuploidy detected in this study was. It would be valuable to see the distribution of the aneuploidies detected and selected against. In addition, the authors refer to a classification in A, B and C embryos. It remains unclear how many were classified as A, B and C and how many A or B (which can be also no-result in the niPGT group) were transferred. It would also be valuable to see those results/age group. Those values are essential to (1) evaluate the accuracy of the niPGT based on comparison with epidemiological/reference data, (2) to understand the selection and potential transfer biases. Specifically required are (a) Embryo classification by preimplantation genetic screening, number of euploid/aneuploid embryos, number of monosomies, trisomies, mosaics, segmental and potential more complex outcomes and classifications (A,B,C).

Response: Thank you for the comment.

The aim of this study is to evaluate the clinical effective of niPGT-A, so we mentioned only a small section in the manuscript, Page 9， lines 201-205. "When stratified analysis in the niPGT-A group, a total of 550 blastocysts were transferred, including 92% of grade A, B blastocysts (506/550) and only 8% of grade C blastocysts (44/550). Statistically significant differences were observed between the blastocyst subgroup with NICS grades A+B and that with NICS grade C regarding clinical pregnancy rate, early miscarriage rate, ongoing pregnancy rate and live birth rate (all $p<0.05$). (Supplementary Table 9).

In the manuscript, only the results of the first planned embryo were focused. Actually, we examined the SCM samples of all the blastocysts. We anticipate that more findings will be presented in further paper. I will show you some results as below:

Female age	NICS grade A	NICS grade B	NICS grade C	Total
35-37	1244(35.4%)	1074(30.5%)	1200(34.1%)	3518
38-42	497(27.4%)	512(28.2%)	807(44.4%)	1816
Total	1741(32.6%)	1586(29.7%)	2007(37.6%)	5334

- I am confused about the outcome: On the one hand, most major intention to treat parameters did not show a significant change: ongoing pregnancy rate, clinical pregnancy, late miscarriage and baby take home rate. However, early miscarriage rate

did show a significant difference: in the niPGT group there were significant less miscarriages. It is surprising that this difference does not percolate into the live birth rates. In light of this question, it would be valuable to see the breakdown numbers of miscarriages in the different age groups.

Response: Thank you for this very insightful comment.

In the study，the early miscarriage rate is defined as the number of pregnancy losses /number of clinical pregnancies after the first planned transfer. The live birth rate is defined as the number of women with live births after the first transfer /number of women randomized to the specific group. As you know, the denominator of the live birth rate is number of randomized women, while that of the miscarriage rate is number of clinical pregnancies.

Our results showed superiority in reducing early miscarriage rate but no differences in primary outcomes. This discrepancy may be attributable to the limited sample size and potential selection bias.

Further research should both expand the sample size and focus on identifying which subgroup of patients will benefit most from niPGT-A, to provide more targeted clinical guidance.

We reviesed it in the Discussion, highlighted revision, Page 13, lines 292-298.

In the subgroup analysis we conducted, we have already performed stratified analyses according to different age groups (Figure 2).

- I cannot find the two figures referred to in the text and figure legends.

Response: We apologize for the inconvenience caused by the missing figure files. The figures have now been uploaded to the submission system as supplementary material.

Minor comments

- The overall success rate for the morphology graded group seems higher than expected based on epidemiological evaluation. Especially for the >38 years of age group, this number is likely lower. It would be good to provide those breakdown numbers.

Response: Thank you for your comment.

We have performed a subgroup analysis stratified by female age. The detailed results are presented in Figure 2 and described in the Results section.

The enrolled women had a good prognosis, which were mentioned in the manuscript. (highlighted revision, Page 15-16, lines 347-350): the inclusion criteria included: “(4)

having retrieved at least 6 oocytes; (5) having at least 2 blastocysts with a morphology score of >4BC or 4CB".

highlighted revision, Page 6, lines 136: "The mean blastocyst number was 4.6 in both groups".

- Discussion line 260: "In our study, patients with 2 or more failed transfer cycles showed no significant differences in clinical outcomes between the 2 groups, suggesting that their implantation failure involves more complex reasons beyond embryo selection." I am confused since in the results, only the outcome after the first embryo transfer is discussed. Where is this statement coming from?

Response: I am sorry that this part was not clear in the original manuscript. The term "number of failed embryo transfer cycles" in our study refers exclusively to the patient's history of previous failed cycles before entering this study. We have revised the manuscript to ensure precision: In the Results section (page 8, lines 195) and Discussion section (highlighted revision, Page 13, Lines 285).

- In introduction and discussion: the potential detrimental effect of embryo biopsy is over represented. There are other studies suggesting that biopsy is not detrimental or only when not applied properly. Those studies could also be referred to.

Response: Thank you for your comment. We have revised and updated the paragraphs in the Introduction and Discussion sections, with the modified portions highlighted in red, highlighted revision, pages 4-5, lines 108-110.

- Title: 'Non-invasive Preimplantation Genetic Testing' is too broadly scoped: This study is not about all forms of NIPT, but specifically about aneuploidy screening. The title should be reworded to 'non-invasive PGT for aneuploidy detection'.

Response: Thank you for your comment. We have revised the title and other sections.

- The authors often use the term ploidy (e.g. first sentence of abstract). The term ploidy is specifically used to indicate the number of genomes, e.g. haploid, diploid, triploid, tetraploid while here aneuploidies, chromomal copy number changes are investigated. Change 'embryos' ploidy' to 'chromosomal aneuploidy in embryos'.

Response: We are very sorry for this error and have corrected it throughout the article.

Reviewer #5 (Remarks to the Author):

I co-reviewed this manuscript with one of the reviewers who provided the listed reports. This is part of the Nature Communications initiative to facilitate training in peer review and to provide appropriate recognition for Early Career Researchers who co-review manuscripts.

Response: We sincerely thank you and the co-reviewer for your time and valuable insights. In revising the manuscript, we have carefully addressed every point raised by all reviewers, including the co-reviewer, with detailed responses and corresponding modifications.

Title: Embryo Selection by Non-invasive Preimplantation Genetic Testing for Aneuploidy in Advanced Aged Women: A Randomized, Clinical Trial

Submission ID: NCOMMS-25-75661-A

Response to Reviewer's Comments:

Reviewer #1 (Remarks to the Author):

The paper has improved, and most questions have been answered in a satisfactory way. However, there are some parts which are not correct or confusing. I list them here:

We thank you for acknowledging the improvements in our revised manuscript. We appreciate the remaining thoughtful comments, which have helped us further refine our work. We have carefully considered each remaining concern and provide below our point-by-point responses.

1. In Introduction, the following part is not correct:

"For example, a study by Rubio et al7. showed that in women between 38 and 41 years, the delivery rate after the first transfer attempt was significantly higher in the PGT-A group per transfer (52.9% vs 24.2%) and per patient (36.0% vs. 21.9%). In a trial by Munne et al., a post hoc analysis indicated a higher ongoing pregnancy rate per embryo transfer in women aged 35–40 years with PGT-A, although no significant difference was found in the primary intention-to-treat analysis for this subgroup 9".

The Rubio trial is very difficult to read and digest and important parts are more or less "hidden". There is no significant improvement in live birth in the PGT-A arm in the ITT analysis (only given in text, not Tables). The Sacchi paper 2019 is a retrospective study and only reporting results per embryo transfer. However, here are three published RCT in older women, not showing any improvement in live birth by PGT-A (Rubio-2017, Munne-2019, Verpoest-2018). This part of your ms should be revised.

Response: Thank you for this valuable clarification. We have now thoroughly revised this section in the revised manuscript. Page 4. lines 94-100, Clean Version.

Given that the current literature reports controversies regarding the application of PGT-A in patients of AMA, we only present that currently the most common indication for PGT-A was AMA, and that PGT-A remains widely used in the advanced-age population.

2. Line 178. I presume you mean embryo transfer? you write transplantation?

Response: Thank you for identifying this terminology error. We have corrected "transplantation" to "embryo transfer" throughout the manuscript (including line 171, Clean Version, and all other instances).

Line 352. I presume you mean PGT-SR and PGT-M. Please clarify.

Response: In this trial, we excluded all patients who intended to undergo PGT (including PGT-A, PGT-SR, and PGT-M) for their assisted reproduction cycle. In China, not all women of advanced maternal age (AMA) choose to undergo PGT-A; the patients enrolled in our study were those who did not undergo a PGT-A cycle. Furthermore, in this trial, only the culture media were analyzed, and no embryos were biopsied.

Line 444. Add that this is a superiority trial, aiming to find a 10% higher ong PR in the PGT-A. arm.

Response: Thanks for this suggestion. We have revised line 444 (now Page 19-20, line 435-437, Clean Version) to explicitly state the trial design.

5. I am still confused about the Flow chart and how 38 and 32 patients in the the mid box are managed. They are all included in the modified ITT (primary analysis). 21+19 did not receive embryo transfer. Were these women then calculated as negative pregnancy? or ? All other women in these boxed received embryo transfer I presume? and you include the results from these transfers in your primary analysis. Please clarify this part as being an extremely important question.

Response: We thank you for highlighting this critical point, and we apologize for the lack of clarity in the original flowchart and description. To address this, we have added a description in the Figure legend and provided a more detailed description in the Statistical Analysis section of Materials and Methods. Page 20, lines 448-450, Clean Version.

（1）Were the 21+19 patients who did not receive embryo transfer calculated as negative pregnancy?

Yes, precisely. These 40 patients (21 in the niPGT-A group and 19 in the morphology group) met the inclusion criteria and underwent niPGT-A testing but did not complete embryo transfer before the project deadline for various

reasons (see Table 1). Consistent with intention-to-treat principles, they were coded as not achieving pregnancy for all pregnancy-related outcomes, including clinical pregnancy, ongoing pregnancy, and live birth. We also prespecified the outcomes for such patients in the statistical analysis plan. Missing data for the primary outcome (ongoing pregnancy after the first embryo transfer) will be imputed as "negative" irrespective of the reason why data are not recorded (e.g., lost to follow-up, no embryo transfer).

(2) Do all other patients in these boxes receive embryo transfer and are included in the primary analysis?

Yes. All remaining patients in the flowchart (17 in the niPGT-A group and 13 in the morphology group) triggered randomization, subsequently underwent single embryo transfer, and their outcomes were included in the mITT analysis as well.

Table 1 Reasons for No Embryo Transfer in the Study Population

Reasons	Patients Number	
	NiPGT-A group	Morphology group
Spontaneous pregnancy	3	3
Divorce	1	1
The patient made the decision to forgo the embryo transfer.	0	1
Withdrew: Performed PGT-A	2	2
Subject requests to withdraw from the study	4	2
Lost to follow-up	8	6
The physical condition is not suitable for embryo transfer: Hypertension or Hypotension	1	2
Did not perform embryo transfer before the deadline	2	2
Total	21	19

Reviewer #4 (Remarks to the Author):

Thank you for your continued review and valuable feedback. We greatly appreciate your recognition that we have addressed most of your previous comments. We also realize that you want us to further revise the manuscript to strengthen the article

and provide a more comprehensive view of the results. Below, please find our responses to the remaining points.

The authors addressed most of my comments. However:

1. The authors should add the analysis of their abstract at the ESHRE 2025 (Poster number:P-094: https://doi.org/10.1093/humrep/deaf097.403) into the manuscript. This analysis provides interesting insights into the potential value of PGT-A and ways forward for potential future studies. I see no reason not to incorporate those findings into the current manuscript.

Response：Thank you for your insightful suggestion. However, incorporating the poster results into the main manuscript may lead to confusion.

First, the designs of the two studies were not exactly the same. This manuscript is a double-blind RCT, the eligible patients were randomized in a 1:1 ratio to the niPGT-A group or morphology group. While the ESHRE poster is not RCT, the patients were divided into three groups according to their blastocysts' morphology.

Second, the data reported in the poster were not completely equivalent to that in the current trial study. In the current RCT manuscript, we used a mITT set, contained 581 couples in the morphology group and 571 in the niPGT-A group, and 1,112 (96.5%) participants underwent first embryo transfer. The poster data involved 1,325 patients, not only the patients in the mITT set, but also some patients not in compliance with mITT standards, such as the recruitments which only had one blastocyst, less than 6 oocytes retrieved, and so on. (The RCT inclusion criteria include: having retrieved at least 6 oocytes; having at least 2 blastocysts with a morphology score of >4BC or 4CB.) (Table 2)

Consequently, to avoid any confusion, we consider it inappropriate to merge the poster data into the present manuscript.

Table 2 Compare the RCT manuscript and the 2025 ESHRE poster

	RCT manuscript	ESHRE poster
design	double-blind RCT	Not all randomized, the patients were divided into three groups according to their blastocysts' morphology
data	mITT set, contained 581 couples in the morphology group and 571 in the niPGT-A group, and 1112 (96.5%) participants	not only the patients in the mITT set, but also some patients not in compliance with mITT standards such as the recruitments which only had one blastocyst, less than 6

	underwent first embryo transfer	oocytes retrieved and so on,

Moreover, we used the 1,112 randomized patients who underwent embryo transfer in the mITT population, we performed the same stratification analysis as presented in the poster. The data comparing the A+B group versus the control group are shown below (Table 3). In patients lacking good-quality embryos, the early miscarriage rate significantly decreased in the niPGT-A group (p=0.043), while the ongoing pregnancy rate did not differ significantly (p = 0.068).

Table 3 Patient stratification in the mITT population based on the presence or absence of good-quality embryos (AA/BA/AB).

Group	Outcomes	niPGT-A group (A+B)	Morphology group	Absolute difference	p-value
With Excellent embryo Patients	Clinical pregnancy rate	56.07% (120/214)	59.40% (139/234)	-3.33%	0.476
	Early miscarriage rate	14.17% (17/120)	20.86% (29/139)	-6.69%	0.160
	Ongoing pregnancy rate	47.66% (102/214)	46.58% (109/234)	1.08%	0.819
	Live birth rate	45.79% (98/214)	44.02% (103/234)	1.77%	0.706
Without Excellent embryo Patients	Clinical pregnancy rate	48.63% (142/292)	46.04% (151/328)	2.59%	0.518
	Early miscarriage rate	**19.01% (27/142)**	**29.14% (44/151)**	**-10.13%**	**0.043**
	**Ongoing pregnancy rate**	**39.04% (114/292)**	**32.01% (105/328)**	**7.03%**	**0.068**
	Live birth rate	36.99% (108/292)	31.40% (103/328)	5.59%	0.143

Given that the mITT population comprised only AMA patients with a good prognosis (excluding those with a single blastocyst or fewer than six retrieved oocytes), it is not representative of the general population aged 35–42 years. Thus, for the poster data, we analyzed datas from all 1,325 screened patients. In addition to the mITT population, we included data from a small number of non-randomized patients. As presented in the poster, among patients with moderate- or low-grade embryos, the niPGT group showed a significantly lower miscarriage rate (19.0% vs. 29.5%, p = 0.037) and a higher ongoing pregnancy rate (39.4% vs. 31.8%, p = 0.049). Therefore, to prevent confusion or misinterpretation, our initial plan was to report these results separately.

2. The results of the aneuploidy screening are not shown.

A. It is not mentioned how many embryos were selected against and what the incidence of aneuploidy detected in this study was. It would be valuable to see the distribution of the aneuploidies detected and selected against. The authors did not yet answer this question. The answer to this question allows to assess the accuracy of the method, by comparing the epidemiological data with known/published incidences.

Response: Thank you for highlighting this important point. In the current RCT, where no blastocysts were discarded, the non-invasive chromosome screening (NICS) technology employed in the niPGT-A group serves as a prioritization tool that ranks embryos according to their estimated probability of euploidy.

We have now added a detailed analysis of aneuploidy screening results. As described in the previous study[1], the NICS model incorporated CNV results at different resolutions to provide a comprehensive score. The classification of CNV results in this analysis was performed according to the whole chromosome-resolution CNV result redefined by a 30% mosaicism threshold.

In this RCT study, we collected all the SCM samples of the blastocysts no matter in the niPGT-A group or the morphology group. Among a total of 5,334 embryos tested, 528 (9.9%) were N/A (amplification failure or non-informative CNV results), and 1433 were classified as euploid. The remaining abnormal embryos were further subdivided into monosomy, trisomy, mosaicism, and complex abnormalities for statistical analysis. The detailed subtypes and their respective proportions are shown in the table 5 below.

Table 5 CNV Classification at Whole-Chromosome Resolution Using a 50% Mosaicism Threshold

Category		Number(proportions)
N/A		528(9.9%)
Euploid		1433(26.9%)
Aneuploid	Monosomy	376(7.0%)
	Trisomy	384(7.2%)
	Complex	723(13.6%)
	Mosaicism	1890(35.4%)

N/A: amplification failure or non-informative CNV results.

3. B. It remains unclear how many were classified as A, B, and C and how many A

or B (which can be also no-result in the niPGT group) were transferred. The authors write that in the manuscript, only the results of the first planned embryo were focused. Moreover, they state that they examined the SCM samples of all the blastocysts and anticipate that more findings will be presented in further paper. I do not understand why they cannot bring those results into this article. I do believe that this information would strengthen the article and provide a more comprehensive view of the results.

Response: Thanks for your proposal. In this study, a total of 5,334 SCMs from randomized patients underwent NICS testing. The distribution of grades A, B, and C

across all samples is shown in the figure below. As described in the previous study[1], embryos were graded as A(0.94 or above), B(0.7–0.94) or C (0.7 or below)based on different cut-off criteria of their euploidy probability. A-grade embryos showed absolute euploidy, low mosaicism or small segmental aneuploidy; B-grade embryos showed a low rate of mosaicism; C-grade embryos had a high mosaicism proportion or a 100% aneuploid pattern. Moreover, in embryos with amplification failure or non-informative CNV results, the NICS grading system displayed null (N/A) results, which were assigned a grade of B in this study.

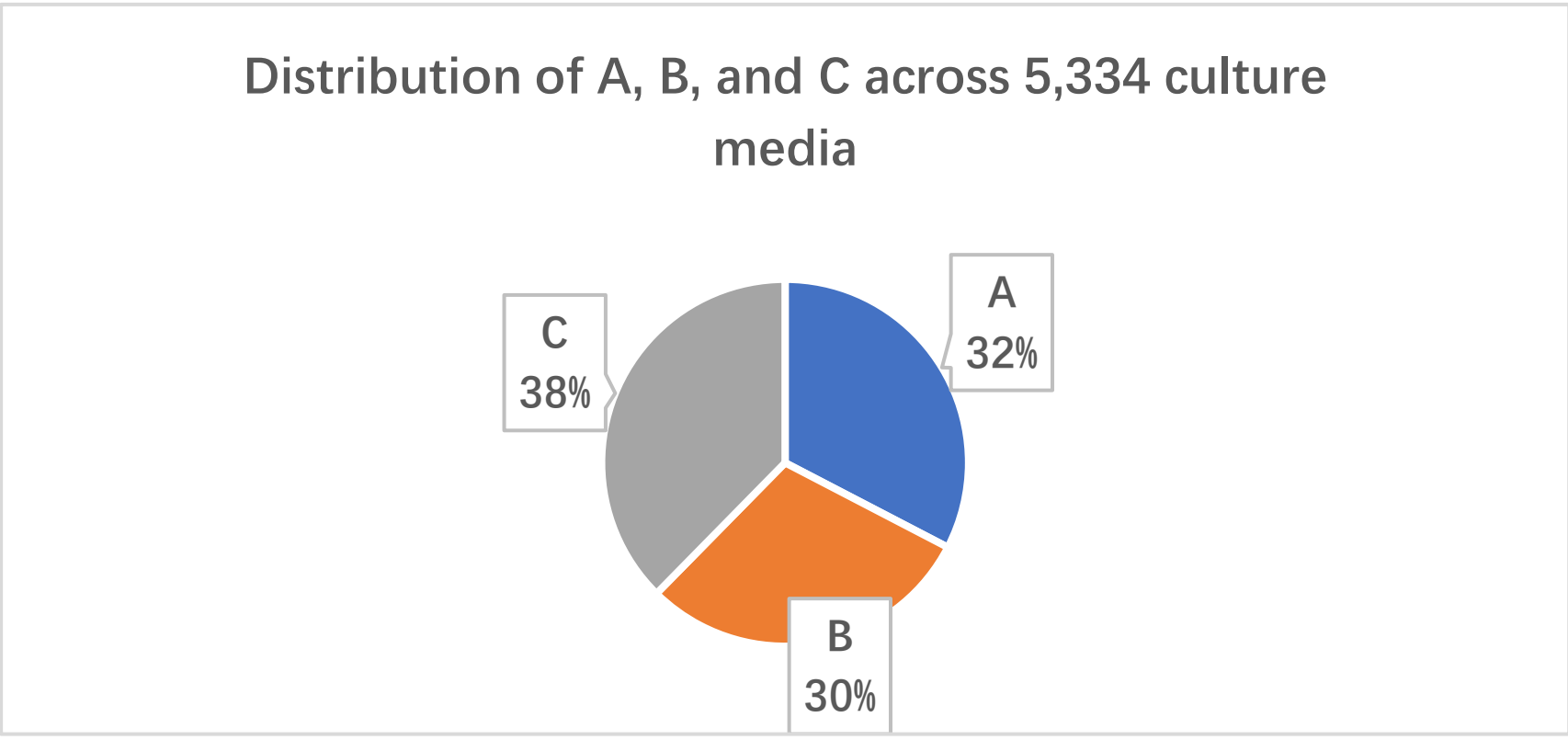

Figrue 1 NICS grade distribution

In the niPGT-A group, the number of patients(n=550) who underwent embryo transfer with grades A, B-NA, B-CNV, and C was 381, 58, 67, and 44, respectively. The clinical outcomes after embryo transfer are shown in the table below. (Table 6)

Table 6 Clinical Outcomes of Grade A/B/C embryos in the niPGT-A Group

niPGT-A results	Patients number	Clinical pregnancy		Early miscarriage		Ongoing pregnancy		Live birth	
**A**	381	193	**50.7%**	30	15.5%	161	42.3%	155	40.7%
**B-total**	125	69	**55.2%**	14	20.3%	55	44.0%	51	40.8%
**B-NA**	58	36	**62.1%**	7	19.4%	29	50.0%	27	46.6%
**B-CNV**	67	33	**49.3%**	7	21.2%	26	38.8%	24	35.8%
**C**	44	10	22.7%	5	50.0%	5	11.4%	5	11.4%

B-NA: amplification failure or non-informative CNV results, assigned a grade of B;

B-CNV: Based on the CNV results, the NICS model gave a grade of B.

Reference

1. Chen L, *et al.* Non-invasive embryo selection strategy for clinical IVF to avoid wastage of potentially competent embryos. *Reprod Biomed Online* **45**, 26-34 (2022).

Title: Embryo Selection by Non-invasive Preimplantation Genetic Testing for Aneuploidy in Women of Advanced Age: A Randomized, Clinical Trial

Submission ID: NCOMMS-25-75661-B

Reviewer #1 (Remarks to the Author):

I am satisfied with the answers from the authors. On page 452 you should however change to embryo transfer (not transplantation).

I miss the revised flow chart.

Response: We sincerely thank the Reviewer for the positive feedback and for taking the time to review our revised manuscript. I am sorry for this mistake and have changed the term from "transplantation" to "embryo transfer".

Regarding the revised flow chart, we have included the updated flowchart in the revised manuscript, which is presented as Figure 1.

Reviewer #4 (Remarks to the Author):

The authors addressed my comments.

Response: We sincerely thank the Reviewer for the positive assessment and for their valuable time spent on our manuscript. We are glad that our revisions have addressed all the concerns raised previously.
